# Utilize Metabolomics Molecular Networking to Guide the Discovery of Chemical Entities from *Cordyceps militaris* Against Blue Light Hazards

**DOI:** 10.1021/acs.jnatprod.6c00427

**Published:** 2026-07-09

**Authors:** Ho-Cheng Wu, Andrea Gu, Ya-Lin Chang, Chung-Kuang Lu, Yi-Chieh Lee, Po-Wei Yu, Tzong-Huei Lee

**Affiliations:** † School of Pharmacy, College of Pharmacy, 534964 Kaohsiung Medical University, Kaohsiung 807378, Taiwan; ‡ Department of Medical Research, Kaohsiung Medical University Hospital, Kaohsiung 807378, Taiwan; § Drug Development and Value Creation Research Center, Kaohsiung Medical University, Kaohsiung 807378, Taiwan; ∥ 33561 Institute of Fisheries Science, College of Life Science, National Taiwan University, Taipei 106319, Taiwan; ⊥ JoinCaring Co., Ltd., Taipei 100024, Taiwan; # National Research Institute of Chinese Medicine, Taipei 112026, Taiwan; ¶ Biomedical Industry Ph.D. Program, 34914 National Yang Ming Chiao Tung University, Taipei 112304, Taiwan

## Abstract

Increasing exposure to blue light has raised concern about retinal injury, particularly in retinal pigment epithelial (RPE) cells. *N*-retinylidene-*N*-retinylethanolamine (A2E), a photosensitive fluorophore associated with age-related macular degeneration, is an important mediator of this process. In this study, feature-based molecular networking guided the identification of cordylutenes A–J (**1**–**10**), a new molecular family of blue light–protective fungal pigments from *Cordyceps militaris*. These metabolites possess a previously unreported symmetric all-*E* C20 polyene dicarbonyl skeleton with carotenoid-like UV absorption. They were characterized at the molecular-family level by shared UV features and diagnostic, internally consistent MS^2^ fragmentation patterns. Cordylutenes A (**1**) and B (**2**) were isolated as representative members for structural characterization. In an A2E-mediated blue-light injury model in ARPE-19 cells, the cordylutene complex (CCL, 1 μg/mL) and cordylutene A (**1**, 0.1 μg/mL) improved cell viability to 84.78 ± 4.02% and 92.14 ± 1.88%, respectively, and reduced intracellular oxidative stress to 63.67 ± 3.33% and 43.89 ± 9.59%, respectively. These findings identify cordylutenes as a new family of fungal pigments with protective effects in an A2E-mediated photo-oxidative retinal cell injury model.

## Graphical abstract

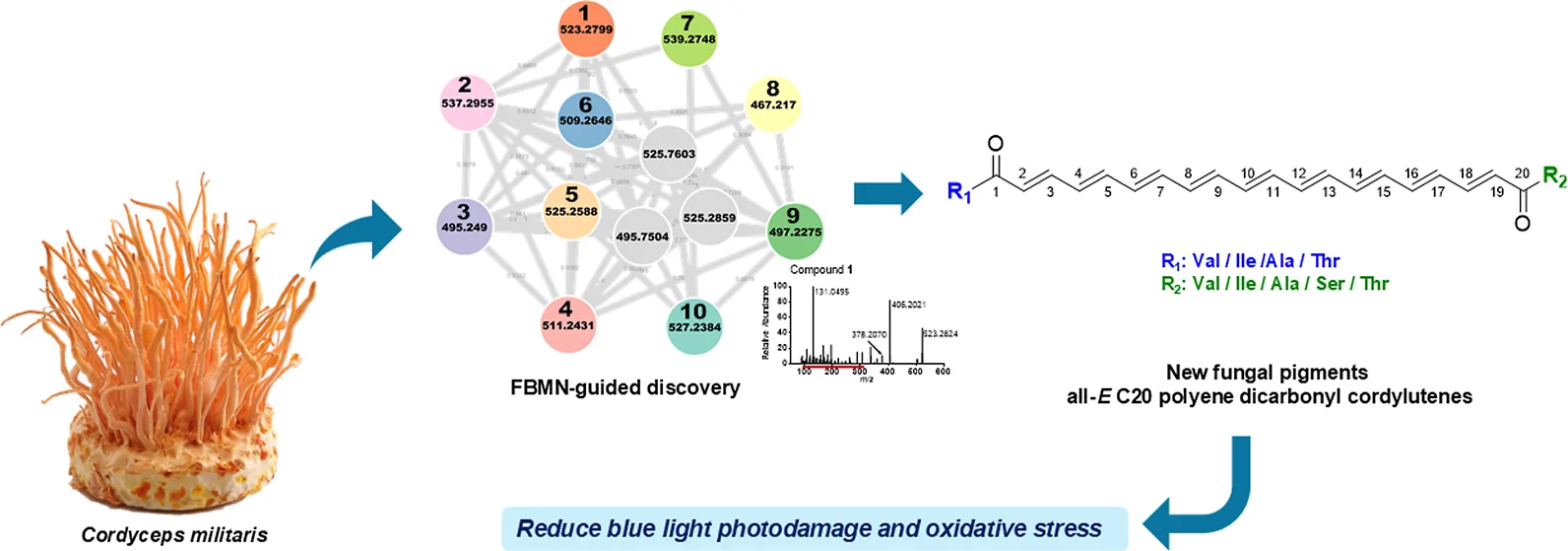

Blue light (380–500 nm), also referred to as high-energy visible (HEV) light, plays important roles in circadian regulation^1^ and visual physiology and supports multiple physiological processes.^2^ However, increasing exposure to blue-enriched light sources has raised concern regarding chronic short-wavelength irradiation.^3,4^ The retina, particularly retinal pigment epithelial (RPE) cells, is highly metabolically active and susceptible to oxidative injury induced by blue light.^5,6^ In this context, *N*-retinylidene-*N*-retinylethanolamine (A2E), a photosensitive fluorophore that accumulates in aging RPE cells and has been implicated in pathological processes associated with age-related macular degeneration, is an important mediator of blue light phototoxicity.^7,8^

Lutein was used as a protective reference compound in this investigation because of its blue light-filtering and antioxidant properties.^9–11^ Naturally enriched in the macula, lutein filters high-energy visible light and mitigates oxidative stress through its ROS-quenching activity.^9^ Its protective effects have been associated with reduced retinal thinning, photoreceptor apoptosis, and mitochondrial dysfunction under blue light exposure.^10,11^

*Cordyceps militaris* is a brightly pigmented entomopathogenic fungus recognized for its diverse bioactive constituents, including cordycepin, polysaccharides, and ergothioneine.^12,13^ Despite extensive studies on these major constituents, the pigmented metabolites of *C. militaris* remain insufficiently characterized. Recent studies have shown that the orange-red pigments of *C. militaris* exhibit absorption maxima between 422 and 478 nm and may suppress ROS generation, suggesting potential relevance to blue light protection.^14,15^ These observations prompted us to examine *C. militaris* pigments as a source of structurally distinctive metabolites with retinal protective activity.

Feature-based molecular networking (FBMN)^16^ was employed to guide the discovery of pigment-derived metabolites. FBMN is a UHPLC-MS/MS-based computational approach implemented in the Global Natural Products Social Molecular Networking (GNPS) platform^17^ for visualizing and organizing complex metabolomic data. By integrating MS precursor and fragmentation information, FBMN facilitates dereplication and structural annotation through comparison with spectral libraries.

This study aimed to isolate, structurally characterize, and evaluate the blue light protective activities of previously unreported pigments from *C. militaris*. FBMN-guided metabolite analysis and bioactivity evaluation were used to examine their chemical features and protective effects in an A2E-mediated blue light injury model.

## Results and Discussion

In the present study, FBMN analysis of *C. militaris* fermentation products revealed a cluster of blue light-absorbing metabolites corresponding to cordylutenes A–J (**1**–**10**), a new molecular family sharing a common symmetric C20 polyene dicarbonyl skeleton with different amino acid substituents. Guided by this result, representative members cordylutenes A (**1**) and B (**2**) were purified and subjected to structure elucidation, whereas the remaining analogues were assigned at the family level based on shared UV features and diagnostic MS^2^ fragmentation patterns. The cordylutene fraction (CCL) and the representative metabolite cordylutene A (**1**) were then evaluated in an A2E-mediated blue light injury model using ARPE-19 cells.

### FBMN Analysis of the *C. militaris* Extracts

The UPLC-MS/MS data of the *C. militaris* extract were used to construct a feature-based molecular network through the GNPS platform (Figure 1A), revealing a cluster of ten related metabolites (compounds **1**–**10**) representing a molecular family. Further, by analyzing their MS^2^ fragments (Figure 1B), the same base peak (*m*/*z*: 131.0494 ± 0.0001) and fragment ions (dark red line area, *m*/*z*: 97–308) indicated that the ten compounds have the same main skeleton. The recurring MS^2^ pieces (*m*/*z* above 308, including 378.1701 ± 0.0006, 394.1653, 406.2021 ± 0.0001, 408.1811 ± 0.0005, 420.2177 ± 0.0006) reveal the appearances of five different substituents. The above evidence indicates that this molecular family contains a symmetric all-*E* C20 polyene dicarbonyl skeleton with variable substituents.

**Figure 1:**
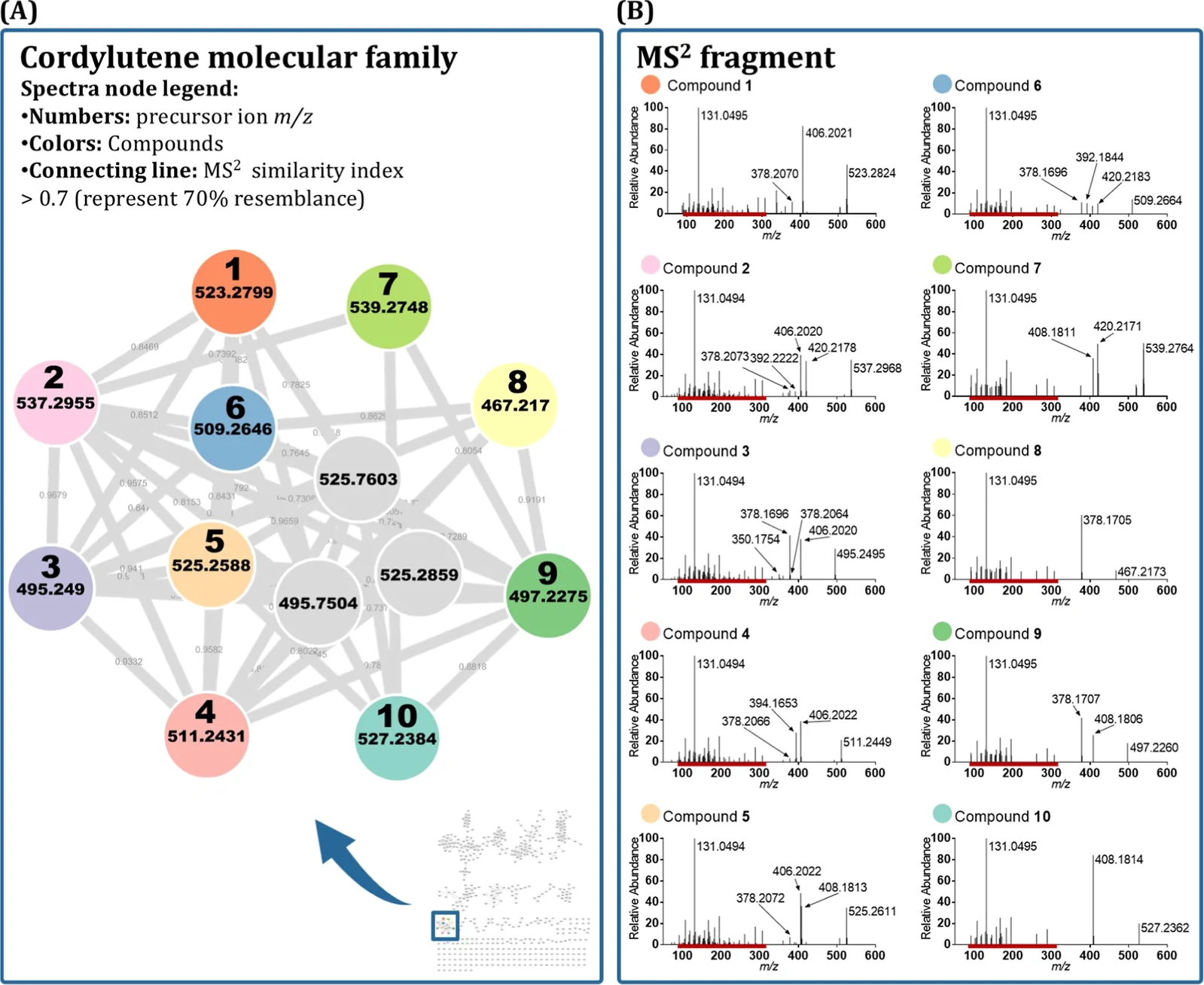
Feature-based molecular networking (FBMN) of *Cordyceps militaris* fermentation. Painted by compounds **1**–**10**. (**A**) Cluster of the cordylutenes molecular family. (**B**) MS^2^ fragments of the cordylutenes molecular family.

To further support this assignment, cordylutenes A (**1**) and B (**2**) were isolated for structure elucidation based on their NMR, UV, IR, MS, and MS^2^ data. The remaining analogues, cordylutenes C–J (**3**–**10**), were assigned by molecular networking and diagnostic MS^2^ fragmentation patterns. Together, these data defined a new family of fungal pigments bearing a symmetric all-*E* C20 polyene dicarbonyl skeleton (Figure 2).

**Figure 2:**
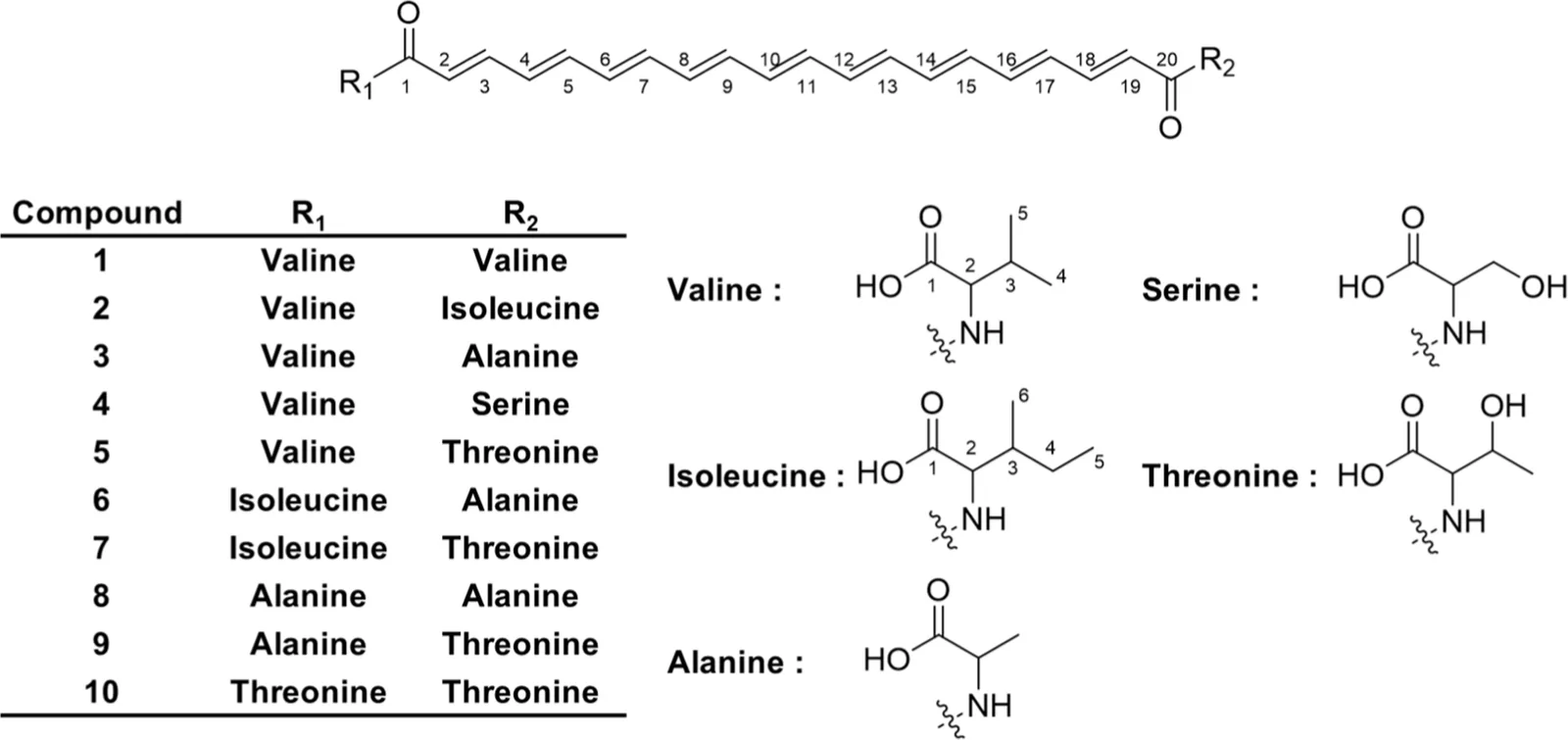
Structures of compounds **1**–**10**.

### Structure Elucidation

Compound **1** was obtained as a reddish amorphous solid. Its molecular formula was established as C_30_H_38_N_2_O_6_ from HRESIMS ([M + H]^+^ at *m*/*z* 523.2803 (calcd. 523.2803 for C_30_H_39_N_2_O_6_^+^)), implying 13 degrees of unsaturation. The IR spectrum exhibited absorption bands attributable to hydroxy (3415 cm^–1^) groups, an NH unit (3272 cm^–1^), a carbonyl group (1712 cm^–1^), C=C stretching vibrations (1640 and 1535 cm^–1^), and *trans* C=C bending (1007 cm^–1^). UV absorption maxima at 420, 444, and 472 nm indicated the presence of an extended carotenoid-like conjugated system. The MS^2^ spectrum of **1** (Figure S11) showed a neutral loss of *m*/*z* 117.0789 from the precursor ion at *m*/*z* 523.2803 to yield an ion at *m*/*z* 406.2014, corresponding to C_5_H_11_NO_2_ (calcd. 117.0790 for C_5_H_11_NO_2_), followed by a second neutral loss of *m*/*z* 117.0791 from *m*/*z* 406.2014 to *m*/*z* 289.1223, which was likewise attributed to C_5_H_11_NO_2_ (calcd. 117.0790 for C_5_H_11_NO_2_). The UV absorption and MS^2^ fragment pattern of isolated compound **1** were consistent with those of the corresponding FBMN node. The MS^2^ spectrum of **1** (Figure S11) also showed the consistent fragment region observed for the FBMN cordylutene cluster, with two dominant MS^2^ fragment ions at *m*/*z* 307.1328 and 289.1223, assigned to C_20_H_19_O_3_ (calcd. 307.1329 for C_20_H_19_O_3_^+^) and C_20_H_17_O_2_ (calcd. 289.1223 for C_20_H_17_O_2_^+^), respectively.

The planar structure of **1** was elucidated by NMR analysis (Table 1) as a symmetric all-*E* C20 polyene dicarbonyl analogue. The ^13^C NMR spectrum showed two equivalent carbonyl carbons at δ_C_ 165.3 (C-1 and C-20), together with 18 olefinic methine carbons assignable to a (2*E*,4*E*,6*E*,8*E*,10*E*,12*E*,14*E*,16*E*,18*E*)-octadeca-2,4,6,8,10,12,14,16,18-nonaene fragment (C-2 to C-19). The ^1^H NMR spectrum displayed 18 olefinic protons consistent with trisubstituted double bonds at δ_H_ 6.24 (1H, d, *J* = 13.8 Hz, H-2 and H-19), 7.08 (1H, t, *J* = 13.8 Hz, H-3 and H-18), 6.44 (1H, t, *J* = 13.8 Hz, H-4 and H-17), 6.68 (1H, t, *J* = 13.8 Hz, H-5 and H-16), 6.46 (1H, m, H-6, H-8, H-9, H-10, H-11, H-12, H-13 and H-15), and 6.52 (1H, t, *J* = 12.0 Hz, H-7 and H-14), consistent with a highly conjugated polyene chain. Correspondingly, 18 methines (C-2 to C-19) were observed in the ^13^C NMR and DEPT spectra. The COSY correlations (Figure 3) of H-2/H-3/H-4/H-5/H-6/H-7/H-8 and H-13/H-14/H-15/H-16/H-17/H-18/H-19, together with HMBC correlations (Figure 3) from H-2 to C-4, H-4 to C-6, H-5 to C-3/C-6, H-16 to C-14/C-18, H-17 to C-15, and H-19 to C-17, and H2BC correlations (Figure 3) from H-9 to C-8, H-10 to C-9, H-11 to C-10/C-12, and H-12 to C-13, established a linear polyene chain. The large coupling constants supported an all-*E* geometry for the polyene chain, which was consistent with the *trans* C=C bending absorption at 1007 cm^–1^ observed in the IR spectrum. Furthermore, HMBC correlations from H-2/H-3 to C-1 and from H-18/H-19 to C-20 located the terminal carbonyl groups at both ends of the polyene chain, thereby defining the central skeleton of **1** as a symmetric all-*E* C20 polyene dicarbonyl core.

**Table 1:** ^1^H NMR and ^13^C NMR Data of Compounds **1** in (CD_3_)_2_SO and **2** in CD_3_OD

position	**1** ^a^ ^b^		**2** ^a^	
	δ_H_, mult (*J* in Hz)	δ_C_	δ_H_, mult (*J* in Hz)	δ_C_
1	-	165.3	-	168.4 or 168.5
2	6.24, d (13.8)	124.9	6.14, d (11.2) or 6.16, d (12.0)	124.7
3	7.08, t (13.8)	139.3	7.20, m	141.8
4	6.44, t (13.8)	131.1	6.42, m	131.8
5	6.68, t (13.8)	139.0	6.65, dd (14.4, 11.2)	140.9
6	6.46, m	133.2	6.41, m	134.0
7	6.52, t (12.0)	136.2	6.51, dd (14.4, 11.2)	137.8
8	6.46, m	133.8	6.42, m	134.9
9	6.46, m	135.1	6.44, m	136.5
10	6.46, m	134.4	6.42, m	135.6
11	6.46, m	134.4	6.42, m	135.6
12	6.46, m	135.1	6.44, m	136.5
13	6.46, m	133.8	6.42, m	134.9
14	6.52, t (12.0)	136.2	6.51, dd (14.4, 11.2)	137.8
15	6.46, m	133.2	6.41, m	134.0
16	6.68, t (13.8)	139.0	6.65, dd (14.4, 11.2)	140.9
17	6.44, t (13.8)	131.1	6.42, m	131.8
18	7.08, t (13.8)	139.3	7.20, m	141.8
19	6.24, d (13.8)	124.9	6.16, d (12.0) or 6.14, d (11.2)	124.7
20	-	165.3	-	168.5 or 168.4
1′	-	173.3	-	177.1
2′	4.18, t (6.6)	57.8	4.37, d (5.6)	60.6
3′	2.06, m	30.1	2.20, m	32.6
4′	0.87, d (6.0)	19.4	0.95, d (6.4)	18.3
5′	0.87, d (6.0)	18.2	0.97, d (7.2)	20.0
NH	8.05, d (6.6)	-	-	-
1″	-	173.3	-	177.0
2″	4.18, t (6.6)	57.8	4.41, d (5.6)	60.0
3″	2.06, m	30.1	1.91, m	39.2
4″	0.87, d (6.0)	19.4	1.21, m/1.55, m	26.2
5″	0.87, d (6.0)	18.2	0.93, t (7.6)	12.1
6″	-	-	0.95, d (6.4)	16.3
NH	8.05, d (6.6)	-	-	-

a ^1^H NMR Data measured at 800 MHz.

b ^13^C NMR Data measured at 200 MHz.

**Figure 3:**
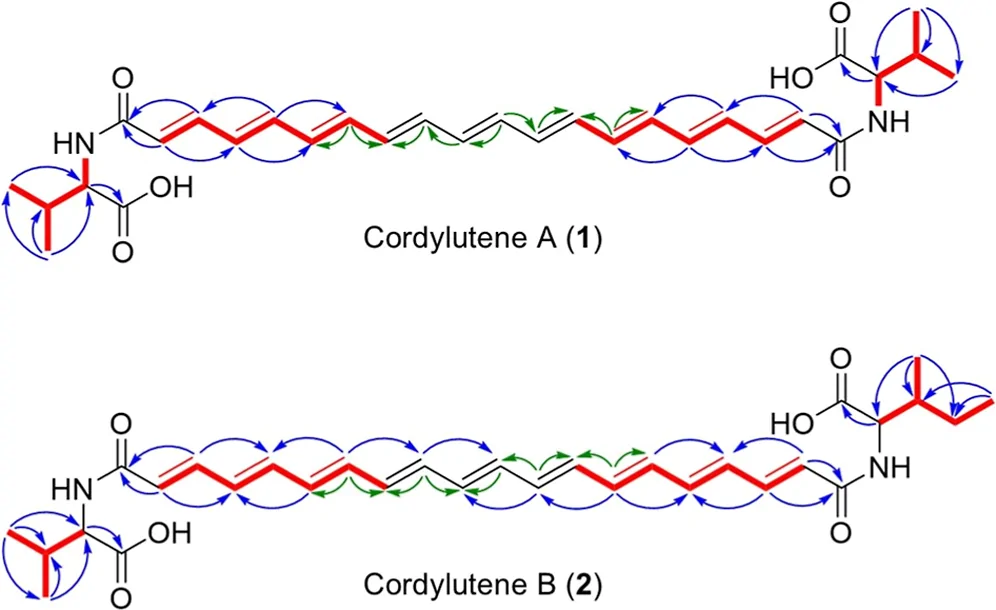
Key ^1^H–^1^H COSY (−), HMBC (→ with blue), and H2BC (→ with green) correlations of compounds **1** and **2**.

The remaining NMR signals, together with the MS^2^ neutral-loss pattern, indicated the presence of two valine (C_5_H_11_NO_2_) derived subunits. Their NMR spectroscopic features were comparable to those of valine,^18^ except for the downfield shifts of the NH-connected methine protons H-2′ and H-2″, consistent with attachment of the amino acid residues to the core through nitrogen atoms. This assignment was supported by COSY correlations (Figure 3) of NH/H-2′/H-3′/H-4′,H-5′ and NH/H-2″/H-3′′/H-4″,H-5″, together with HMBC correlations (Figure 3) from H-2′ to C-1′, and from H-4′ and H-5′ to C-2′/C-3′/C-4′, with the corresponding correlations observed for the second unit. Accordingly, the planar structure of **1** was established as a bis­(amino acid-derived) derivative of the C20 polyene dicarbonyl skeleton bearing two valine residues, and the compound was designated cordylutene A.

Compound **2** was also isolated as a reddish amorphous solid, and the molecular formula was determined to be C_31_H_40_N_2_O_6_ from HRESIMS ([M + H]^+^ at *m*/*z* 537.2966 (calcd. 537.2959 for C_31_H_41_N_2_O_6_^+^)), indicating 13 degrees of unsaturation. The IR spectrum exhibited absorption bands attributable to hydroxy (3416 cm^–1^) groups, an NH unit (3310 cm^–1^), a carbonyl group (1712 cm^–1^), C=C stretching vibrations (1636 and 1523 cm^–1^), and *trans* C=C bending (1004 cm^–1^), closely resembling those of **1**. Its UV absorption maxima at 420, 444, and 472 nm likewise resembled those of **1**, suggesting the same extended conjugated chromophore. Comparison of the NMR data of **2** with those of **1** (Table 1) indicated that both compounds shared the same symmetric all-*E* C20 polyene dicarbonyl skeleton, but one valine-derived unit in **1** was replaced by an isoleucine-derived unit in **2**. This interpretation was supported by the MS^2^ spectrum of **2** (Figure S23), which showed neutral losses of 117.0790 and 131.0952 Da, corresponding to C_5_H_11_NO_2_ (calcd 117.0790) and C_6_H_13_NO_2_ (calcd 131.0946), respectively. The NMR data of the second amino acid residue were consistent with isoleucine,^19^ and this assignment was supported by COSY correlations of H-2″/H-3′′/H-4′′/H-6″ and H-4′′/H-5″, together with HMBC correlations from H-5″ to C-3′′/C-4″, from H-6″ to C-2′′/C-3′′/C-4″, and from H-2″ to C-1′′. Thus, the planar structure of **2** was established as a valine/isoleucine analogue of **1**, and the compound was designated cordylutene B.

The absolute configurations of the amino acid substituents of **1** and **2** were determined separately after acid hydrolysis. Hydrolysis of **1** released a single amino acid residue, whereas hydrolysis of **2** released two amino acid residues. Comparison of the hydrolysates with authentic standards by HPLC established the amino acid residues in **1** as l-valine and those in **2** as l-valine and l-isoleucine (Figure 4). Thus, the stereochemical assignments were based on analysis of the liberated amino acid components rather than directly on the intact molecules, providing an independent line of evidence for the identities of the appended amino acid residues.

**Figure 4:**
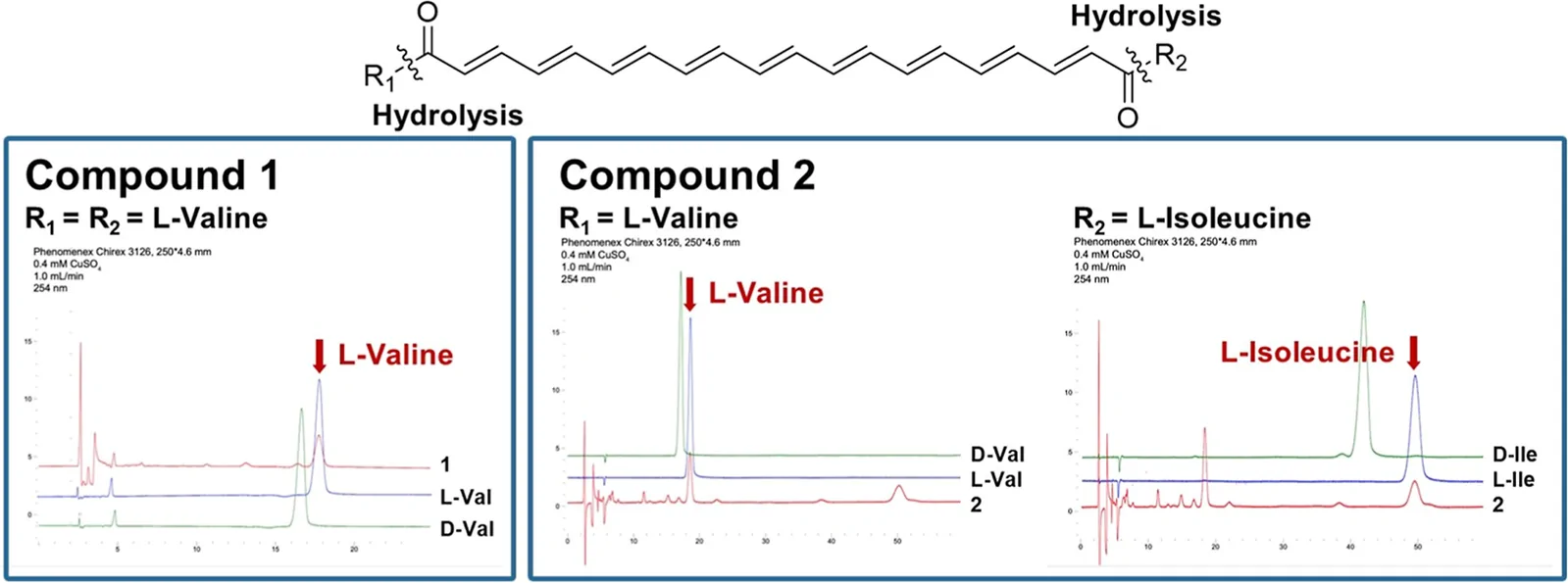
Characterization of released amino acid residues from acid hydrolysis of compounds **1** and **2**.

Having established the core skeleton and amino acid substituents in **1** and **2**, the remaining members of this molecular family were assigned based on their MS^2^ fragmentation behavior in combination with molecular networking analysis (Figures 1B, S11 and S12). As summarized in Figure 5, the cordylutene family exhibited a highly conserved fragmentation pattern characterized by diagnostic fragment ions and amino acid-specific neutral losses. Neutral-loss values of 89.0477, 105.0426, 117.0790, 119.0582, and 131.0946 Da corresponded to alanine, serine, valine, threonine, and isoleucine residues, respectively, enabling assignment of the amino acid-derived substituents at *R*_1_ and *R*_2_ (Table 2). The consistent observation of conserved fragment ions at *m*/*z* 307.1329 ([C_20_H_19_O_3_]^+^), *m*/*z* 289.1223 ([C_20_H_17_O_2_]^+^), and the common base peak at *m*/*z* 131.0491 ([C_9_H_7_O]^+^) further supported the presence of a shared C20 polyene dicarbonyl-derived core skeleton across the molecular family. On the basis of the complete NMR characterization of **1** and **2**, the HPLC-based identification of their hydrolyzed amino acid residues, and the diagnostic MS^2^ fragmentation features observed throughout the cluster, cordylutenes A–J (**1**–**10**) were characterized as members of a molecular family sharing the same core skeleton but bearing different amino acid residues at *R*_1_ and *R*_2_. Accordingly, compounds **3**–**10** were assigned as analogues of **1** and **2**.

**Figure 5:**
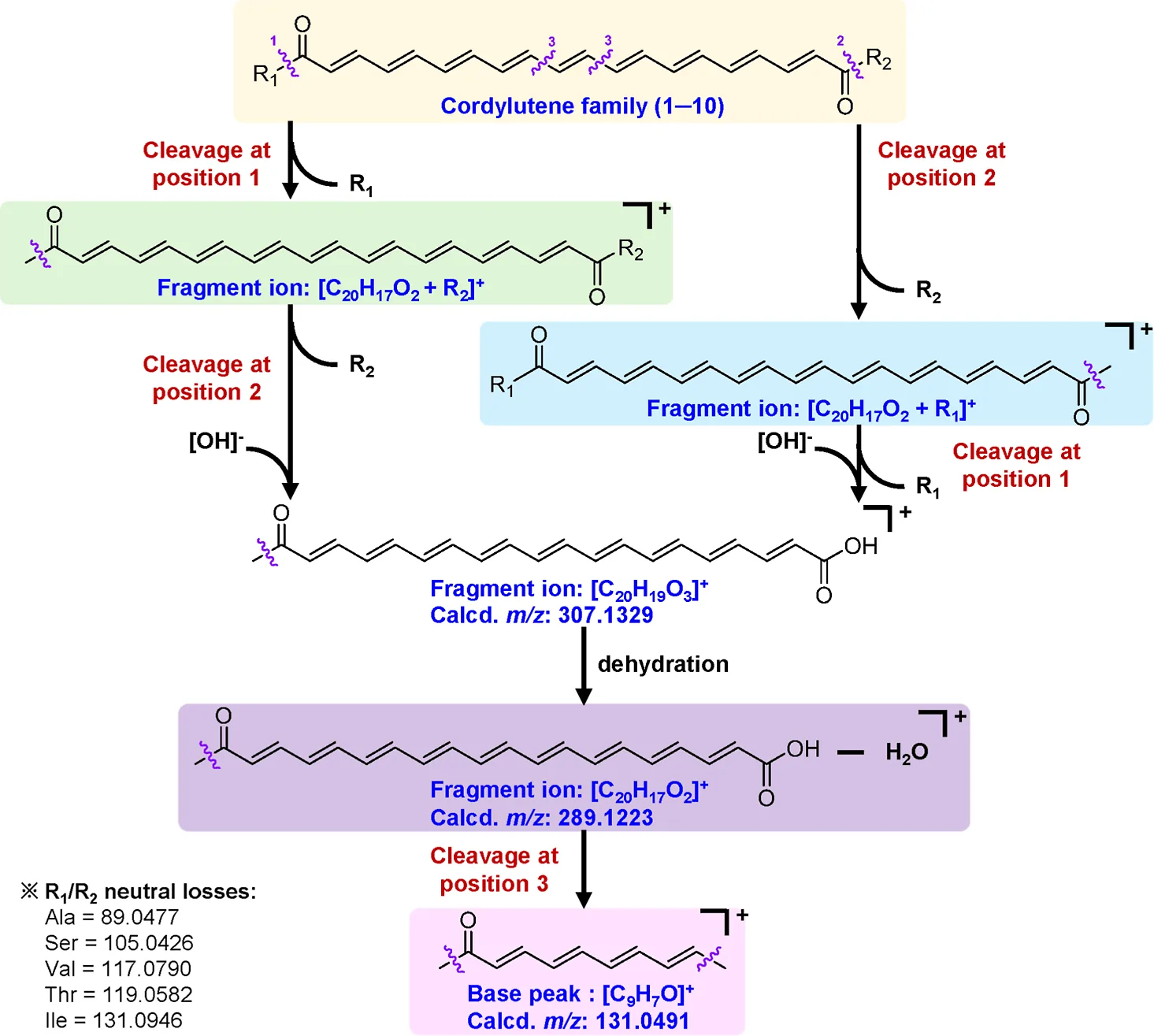
Proposed diagnostic MS^2^ fragmentation pattern of the cordylutene family (**1**–**10**). Cleavage at position 1 or 2 generates fragment ions of the type [C_20_H_17_O_2_ + *R*_2_]^+^ and [C_20_H_17_O_2_ + *R*_1_]^+^, respectively. Subsequent fragmentation affords a conserved ion at *m*/*z* 307.1329 ([C_20_H_19_O_3_]^+^), followed by dehydration to yield the shared core fragment at *m*/*z* 289.1223 ([C_20_H_17_O_2_]^+^). Further cleavage at position 3 gives the common base peak at *m*/*z* 131.0491 ([C_9_H_7_O]^+^). The neutral-loss patterns corresponding to the *R*_1_/*R*_2_ amino acid-derived substituents are 89.0477 (Ala), 105.0426 (Ser), 117.0790 (Val), 119.0582 (Thr), and 131.0946 (Ile).

**Table 2:** Key MS^2^ Fragment Pattern of the Cordylutene Molecular Family^c^

			*m*/*z*			*m*/*z*			
compound	*R*_1_	*R*_2_	precursor ion	fragment ion [C_20_H_17_O_2_ + *R*_2_]^+^ (cleavage 1)	fragment ion [C_20_H_17_O_2_ + *R*_1_]^+^ (cleavage 2)	loss pattern (cleavage 1)	loss pattern (cleavage 2)	fragment ion [C_20_H_17_O_2_]^+^ (cleavage 1 + 2)	base peak
**1** ^a^	valine	valine	523.2809	406.2014	406.2014	117.0795	117.0795	289.1223	131.0492
**2** ^a^	valine	isoleucine	537.2966	420.2176	406.2014	117.0790	131.0952	289.1224	131.0492
**1** ^b^	valine	valine	523.2799	406.2021	406.2021	117.0778	117.0778	289.1225	131.0495
**2** ^b^	valine	isoleucine	537.2955	420.2178	406.2020	117.0777	131.0935	289.1227	131.0494
**3** ^b^	valine	alanine	495.2490	378.1696	406.2020	117.0794	89.0470	289.1229	131.0494
**4** ^b^	valine	serine	511.2431	394.1653	406.2022	117.0778	105.0409	289.1229	131.0494
**5** ^b^	valine	threonine	525.2588	408.1813	406.2022	117.0775	119.0566	289.1230	131.0494
**6** ^b^	isoleucine	alanine	509.5646	378.1696	420.2183	131.0950	89.0463	289.1227	131.0495
**7** ^b^	isoleucine	threonine	539.2748	408.1811	420.2171	131.0937	119.0577	289.1218	131.0495
**8** ^b^	alanine	alanine	467.2170	378.1705	378.1705	89.0465	89.0465	289.1230	131.0495
**9** ^b^	alanine	threonine	497.2275	408.1806	378.1707	89.0469	119.0568	289.1222	131.1495
**10** ^b^	threonine	threonine	527.2384	408.1814	408.1814	119.0570	119.0570	289.1230	131.0495

a MS data and MS^2^ fragments from isolated pure compound.

b MS data and MS^2^ fragments from FBMN.

c *R*_1_ and *R*_2_ substituents molecular formula and weights: Alanine: calcd. 89.0477 for C_3_H_7_NO_2_, Serine: calcd. 105.0426 for C_3_H_7_NO_3_, Valine: calcd. 117.0790 for C_5_H_11_NO_2_, Threonine: calcd. 119.0582 for C_4_H_9_NO_3_, Isoleucine: calcd. 131.0946 for C_6_H_13_NO_2_.

These compounds underwent cleavage to yield amino acid-derived fragments at either *R*_1_ or *R*_2_, plausibly through retro-heteroene-type processes, together with conserved ions representing the common polyene core. Five R group major neutral-loss patterns, 89.0467 ± 0.0004, 105.0409, 117.0784 ± 0.0011, 119.0571 ± 0.0006, and 131.0944 ± 0.0009 Da, corresponded to alanine, serine, valine, threonine, and isoleucine residues, respectively (Table 2). In addition, a conserved fragment at *m*/*z* 289.1227 ± 0.0009 (calcd 289.1223 for C_20_H_17_O_2_^+^) was consistently observed across the cluster, supporting a shared C20 polyene dicarbonyl-derived core skeleton, while the common base peak at *m*/*z* 131.0495 ± 0.0001 (calcd 131.0491 for C_9_H_7_O^+^) provided further support for a uniform fragmentation manifold. Based on the complete NMR characterization of **1** and **2**, the HPLC-based identification of their hydrolyzed amino acid residues, and the diagnostic MS^2^ patterns observed across the cluster, cordylutenes A–J (**1**–**10**) were characterized as members of a molecular family sharing the same core skeleton but bearing different amino acid residues at *R*_1_ and *R*_2_. Accordingly, compounds **3**–**10** were assigned as analogues of **1** and **2**.

### Effect of Amino Acid Supplementation on Cordylutene Production

To further examine the relationship between amino acid availability and cordylutene production, the amounts of compounds **1**–**3**, **4**, and **10** were measured in methanol extracts of *C. militaris* cultured in the presence of an additional 10 mM glycine or one of the amino acids represented in the cordylutene substituent. Compounds **1**, **2**, **4**, and **10** increased significantly when the corresponding amino acid residue was supplemented (Table 3). For example, cordylutene A (**1**; *R*_1_ = valine, *R*_2_ = valine) increased to 292.85 ± 20.47% of the control in plate culture and 157.84 ± 2.62% in solid FB culture upon addition of 10 mM valine. Cordylutene B (**2**; *R*_1_ = valine, *R*_2_ = isoleucine) increased to 140.97 ± 1.15% in solid FB culture upon addition of 10 mM isoleucine. Cordylutene E (**4**; *R*_1_ = valine, *R*_2_ = threonine) increased to 209.05 ± 16.14% in plate culture and 188.85 ± 15.20% in solid FB culture upon the addition of 10 mM threonine, whereas cordylutene J (**10**; *R*_1_ = threonine, *R*_2_ = threonine) increased to 762.25 ± 39.88% in plate culture under the same supplementation condition. These results support a biosynthetically relevant association between amino acid composition in the culture medium and cordylutene production.

**Table 3:** Compound Contents Obtained from Plate and Solid Cultures Supplemented Additionally with Different Amino Acids^a^

culture type		cordylutene A (**1**)(*R*_1_ = valine, *R*_2_ = valine)	cordylutene B (**2**)(*R*_1_ = valine, *R*_2_ = isoleucine)	cordylutene C (**3**)(*R*_1_ = valine, *R*_2_ = alanine)	cordylutene E (**5**)(*R*_1_ = valine, *R*_2_ = threonine)	cordylutene J (**10**)(*R*_1_ = threonine, *R*_2_ = threonine)
**plate**	**control**	100 ± 4.71	100 ± 10.86	100 ± 8.06	100 ± 4.28	100 ± 9.03
	**valine**	292.85 ± 20.47 ***	74.13 ± 3.84	77.55 ± 4.27	96.61 ± 3.61	40.26 ± 3.31
	**isoleucine**	47.5 ± 1.00	119.52 ± 2.10	117.69 ± 4.39	94.93 ± 3.42	166.69 ± 11.95
	**alanine**	144.19 ± 10.14	113.32 ± 12.73	127.57 ± 9.37	147.06 ± 6.94	191.36 ± 19.74
	**serine**	124.61 ± 7.90	112.45 ± 10.22	98.21 ± 11.47	108.79 ± 7.39	98.58 ± 12.12
	**threonine**	61.73 ± 1.18	58.45 ± 5.10	116.66 ± 7.75	209.05 ± 16.14 ***	762.25 ± 39.88 ***
	**glycine**	124.01 ± 6.60	124.58 ± 8.17	123.34 ± 8.96	112.69 ± 6.00	104.03 ± 11.58
**solid**	**control**	100.00 ± 5.37	100.00 ± 0.78	100.00 ± 4.66	100.00 ± 8.15	-
	**valine**	157.84 ± 2.62 ***	56.89 ± 7.96	76.20 ± 0.92	91.01 ± 0.48	-
	**isoleucine**	80.74 ± 3.54	140.97 ± 1.15 ***	98.29 ± 7.67	95.66 ± 10.73	-
	**alanine**	93.86 ± 5.37	98.57 ± 4.44	105.00 ± 4.49	108.20 ± 3.40	-
	**serine**	73.73 ± 2.09	77.58 ± 5.95	81.04 ± 7.21	76.91 ± 4.96	-
	**threonine**	59.48 ± 2.29	59.08 ± 4.10	70.38 ± 1.57	188.85 ± 15.20 ***	-
	**glycine**	73.67 ± 0.17	74.51 ± 0.28	77.91 ± 1.35	75.45 ± 3.70	-

a Compound content ratios of control. Results are presented as mean ± SD (*n* = 3). ***p < 0.001 compared with the control group.

### Effects of ARPE-19 Cell Viability

The cordylutene fraction (CCL, cordylutene complex containing **1**–**10**) and cordylutene A (**1**) were evaluated for their effects on the viability of ARPE-19 cells. As shown in Table 4, ARPE-19 cells maintained high viability after treatment with either CCL or cordylutene A (**1**). Treatment with CCL at 1, 5, and 10 μg/mL resulted in cell viabilities of 105.92%, 110.30%, and 98.97% relative to the control, respectively, whereas treatment with cordylutene A (**1**) at the same concentrations gave cell viabilities of 101.20%, 94.50%, and 96.48%, respectively. These results indicate that neither CCL nor cordylutene A (**1**) exhibited significant cytotoxicity toward ARPE-19 cells under the tested conditions.

**Table 4:** Effects of the CCL and Cordylutene A (**1**) on ARPE-19 Cell Viability^a^

treatment	concentration (μg/mL)	cell viability (%)
Control	-	100.00 ± 3.34
Lutein	17.1	90.30 ± 6.35
CCL	10	98.97 ± 4.29
	5	110.30 ± 2.23
	1	105.92 ± 6.61
Cordylutene A (**1**)	1	96.48 ± 0.60
	0.5	94.50 ± 2.75
	0.1	101.20 ± 2.23

a Data are expressed as mean ± SD (*n* = 3). Cell viability was determined by the MTT assay after 24 h treatment and is presented relative to the untreated control group, which was set at 100%. Lutein (17.1 μg/mL) was used as a reference compound.

### Protection Against A2E-Mediated Blue Light Photodamage

The protective effects of CCL and cordylutene A (**1**) against A2E-mediated photodamage were subsequently evaluated in ARPE-19 cells (Table 5). Exposure of ARPE-19 cells to A2E followed by blue light irradiation markedly reduced cell viability, as shown by the disease model group, which retained only 24.19 ± 2.31% viability relative to the control. In contrast, lutein (17.1 μg/mL), used as a positive control, improved cell viability to 75.30 ± 5.13%. Under the same conditions, both CCL and cordylutene A (**1**) conferred significant protection. Among the tested concentrations, cordylutene A (**1**) at 0.1 μg/mL afforded the highest protective effect, restoring cell viability to 92.14 ± 1.88%. CCL also improved cell survival, with the highest viability reaching 84.78 ± 4.02% at 1 μg/mL. These results indicate that both the crude cordylutene fraction and the purified metabolite attenuated A2E-mediated blue light photodamage in ARPE-19 cells, with cordylutene A (**1**) showing the strongest protective effect under the tested conditions.

**Table 5:** Effects of the CCL and Cordylutene A (**1**) on Cell Viability and Intracellular Oxidative Stress in A2E-Mediated Blue Light-Injured ARPE-19 Cells^a^

treatment		concentration (μg/mL)	cell viability (%)	intracellular ROS Level (%)
control	-		100.00 ± 1.79^a^	56.16 ± 5.86^ac^
model (A2E + blue light)	-	-	24.19 ± 2.31^f^	79.77 ± 4.51^af^
	Lutein	17.1	75.30 ± 5.13^d^	49.36 ± 5.33^ab^
	CCL	10	66.84 ± 0.34^e^	68.20 ± 7.12^ae^
		5	80.94 ± 6.75^d^	67.51 ± 2.85^ae^
		1	84.78 ± 4.02^c^	63.67 ± 3.33^ad^
	Cordylutene A (**1**)	1	70.51 ± 7.44^e^	69.61 ± 4.36^ae^
		0.5	79.49 ± 3.59^d^	61.53 ± 6.10^ad^
		0.1	92.14 ± 1.88^b^	43.89 ± 9.59^ab^

a Data are expressed as mean ± SD (*n* = 3). Cell viability is presented relative to the untreated control group, which was set at 100%. Intracellular ROS levels were determined by the DCFDA assay and are expressed as relative fluorescence intensity. Except for the normal control group, cells were pretreated with the indicated samples, incubated with A2E (10 μM), and then exposed to blue light (420–439 nm) for 9 min. Lutein (17.1 μg/mL) was used as a positive control. CCL denotes the cordylutene-enriched fraction containing cordylutenes A–J (**1**–**10**). Within each column, values sharing the same letter are not significantly different (*p* > 0.05), whereas values with different letters are significantly different (*p* < 0.05) according to one-way ANOVA followed by Duncan’s multiple range test.

### Reduction of Intracellular Oxidative Stress

The effects of CCL and cordylutene A (**1**) on intracellular oxidative stress were further examined in the same model (Table 5). Following blue light irradiation, ARPE-19 cells exposed to A2E showed a marked increase in oxidative stress to 79.77 ± 4.51%, compared with 56.16 ± 5.86% in the control group. Lutein (17.1 μg/mL) reduced this value to 49.36 ± 5.33%, serving as a positive control. Both CCL and cordylutene A (**1**) significantly attenuated intracellular oxidative stress. Notably, cordylutene A (**1**) at 0.1 μg/mL reduced oxidative stress to 43.89 ± 9.59%, a value lower than that observed for lutein under the same assay conditions. These findings indicate that the protective effects of CCL and cordylutene A (**1**) are associated, at least in part, with suppression of intracellular oxidative stress in the A2E/blue light-challenged ARPE-19 model.

## Conclusion

In summary, chemical investigation of the pigment-producing metabolites of *C. militaris* led to the characterization of a new molecular family, cordylutenes A–J (**1**–**10**), featuring a symmetric all-*E* C20 polyene dicarbonyl skeleton appended with different amino acid residues. The structures of representative members **1** and **2** were established by detailed spectroscopic analysis, whereas the remaining analogues were assigned at the family level based on diagnostic MS^2^ fragmentation patterns in combination with molecular networking analysis. Amino acid supplementation experiments further supported a biosynthetically relevant relationship between precursor availability and cordylutene production. In the A2E-mediated blue light injury model, both the cordylutene complex and cordylutene A (**1**) showed no significant cytotoxicity toward ARPE-19 cells and exerted protective effects against photodamage and intracellular oxidative stress. Among the tested samples, cordylutene A (**1**) displayed the strongest activity, particularly at 0.1 μg/mL. Together, these findings identify cordylutenes as structurally distinctive fungal pigments and suggest that they merit further study as natural products with protective effects in a photo-oxidative retinal cell injury model.

## Experimental Section

### General Experimental Procedures

High-resolution mass spectrometry (MS) and MS/MS data were obtained using Thermo Dionex UltiMate 3000 ultrahigh-performance liquid chromatography (UHPLC) coupled to a Thermo Q-Exactive Plus Orbitrap mass spectrometer system (Thermo, Waltham, MA, USA) and Thermo Dionex UltiMate 3000 UHPLC coupled to a Thermo Q-Exactive Focus Orbitrap mass spectrometer system (Thermo, Waltham, MA, USA). MPLC was carried out with glass columns packed with RP-C18 gel (YMC ODS-A-HG, 12 nm, S-50, Kyoto, Japan), performed using a Waters 515 HPLC Pump (Waters, Milford, MA, USA). Further purification steps were performed by high-performance liquid chromatography (HPLC) using the Shimadzu SCL-40 system with an SPD-M40 photodiode array (PDA) detector (Shimadzu, Kyoto, Japan). ^1^H, ^13^C, 1D, and 2D nuclear magnetic resonance (NMR) spectra were acquired on a Bruker AVIII 800 MHz NMR with Cryoprobe (Bruker, Bremen, Germany). Ultraviolet (UV) spectra were measured on a Hitachi UHPLC ChromasterUltra Rs system with a PDA detector (Hitachi, Tokyo, Japan). Infrared (IR) spectra were recorded on a Jasco FT/IR-4600 spectrometer (Jasco, Tokyo, Japan).

### Fungus Material and Fermentation with the OSMAC Approach

*C. militaris*, *C. militaris* fermentation, and OSMAC fermentation were from JoinCaring Co., Ltd. (Taipei, Taiwan). A voucher specimen was deposited at the lab-database of Prof. Tzong-Huei Lee, Institute of Fisheries Science, College of Life Science, National Taiwan University.

### Feature-Based Molecular Networking (FBMN) of *C. militaris*

The *C. militaris* extract was dissolved in methanol, then filtered through 0.2 μm PTFE syringe filters (Pall, NY, USA) to collect MS and MS/MS data. The following parameters were used for analyzing: Thermo Hypersil GOLD RP-C18 UHPLC column, water/95% acetonitrile 95/5 to 0/100 with 0.1% formic acid and 2 mM ammonium formate, the top three precursor ions were fragmented with energies 10, 20, and 40 eV. Data converted by MSConvert software^20^ to mzML files, processed by MZmine software,^21^ exported for FBMN analysis^16^ in the GNPS platform,^17^ then output as a Cytoscape^22^ file for feature structure annotation. The visualized FBMN network was generated with both cosine scores above 0.7 in the linkages between nodes and six matched fragment ions at least (job ID: b7c70abcdb3d48369f9935236916940b).

### Extraction and Isolation

The dried *C. militaris* was extracted at room temperature with 70% methanol, centrifuged at 5000 rpm/1 min, the supernatant liquor was taken, and the precipitate was extracted following the above procedure 5 times. The supernatant was concentrated to yield *C. militaris* extract (1.15 g), then subjected to MPLC (RP-C18 gel, water/acetone 1/2, column size: 1.5 × 30 cm) to produce 13 fractions (Fr. 1–13). Fr. 1 (729.4 mg) was subjected to MPLC (RP-C18 gel; water/methanol 20/1 to 3/7, then washed with 100% acetone, column size: 1.5 × 30 cm) to afford 10 fractions (Fr. 1–1–1–10) with compound **1** (4.8 mg, as Fr.1–8), Fr.1–9 (29.7 mg) was subjected to an HPLC column (YMC semiprep RP-C18 column:10 × 250 mm I.D. S-5 μm, 12 nm, water/acetonitrile 1/1 with 0.2% formic acid) to obtain compound **2** (1.3 mg).

### Spectral Data of Isolated Previously Undescribed Compounds

Cordylutene A (**1**): Reddish amorphous solid; IR (ATR) ν_max_: 3415 (−OH), 3272 (−NH), 1712 (C=O), 1640, 1535 (C=C stretching), and 1007 (*trans* C=C bending) cm^–1^; UV λ_max_ (UHPLC-PDA): 420, 444, and 472 nm; ^1^H NMR ((CD_3_)_2_SO, 800 MHz) and ^13^C NMR data ((CD_3_)_2_SO, 200 MHz): see Table 1; HRESIMS [M + H]^+^ at *m*/*z* 523.2803 (calcd. 523.2803 for C_30_H_39_N_2_O_6_^+^).

Cordylutene B (**2**): Reddish amorphous solid; IR (ATR) ν_max_: 3416 (−OH), 3310 (−NH), 1712 (C=O), 1636, 1523 (C=C stretching), and 1004 (*trans* C=C bending) cm^–1^; UV λ_max_ (UHPLC-PDA): 420, 444, and 472 nm; ^1^H NMR (CD_3_OD, 800 MHz) and ^13^C NMR data (CD_3_OD, 200 MHz): see Table 1; HRESIMS [M + H]^+^ at *m*/*z* 537.2966 (calcd. 537.2959 for C_31_H_41_N_2_O_6_^+^).

### Cell Culture and Reagents

ARPE-19 cells (Human retinal pigment epithelial cells, BCRC 60383) were obtained from the Food Industry Research and Development Institute. Cells were maintained in a 1:1 mixture of Dulbecco’s Modified Eagle Medium (DMEM) and Ham’s F12 medium supplemented with 10% fetal bovine serum (FBS), 1.2 g/L sodium bicarbonate, 2.5 mM l-glutamine, 15 mM HEPES, and 0.5 mM sodium pyruvate. Cells were cultured in a humidified incubator at 37 °C, 5% CO_2_. The cordylutene complex (CCL, provided as Cordylutein by JoinCaring Co., Ltd., Taipei, Taiwan) was defined as a partially purified fraction enriched in cordylutenes A–J (**1**–**10**) from *C.militaris*. Test samples were dissolved in dimethyl sulfoxide (DMSO), and the final DMSO concentration in each treatment group was adjusted to the same level as that of the corresponding vehicle control.

### Cell Viability Assay

For the cell viability assay, cells were first detached using trypsin with EDTA and resuspended in culture medium, after which the cell suspension was adjusted to a concentration of 1.0 × 10^5^ cells/mL. A volume of 100 μL (1.5 × 10^4^ cells/well) was seeded into each well of a 96-well plate and cultured for 24 h. The medium was then replaced with 100 μL of the corresponding treatment solutions and incubated for another 24 h. Following treatment, the medium was removed and each well received 50 μL of medium containing 1.0 mg/mL MTT. After a 2 h incubation in the dark to allow for formazan crystal formation, the MTT-containing medium was discarded and 200 μL of DMSO was added to each well to dissolve the formazan crystals. Absorbance was measured at 570 nm using a microplate reader, and cell viability was expressed relative to the untreated control group.

### Photodamage Assay

ARPE-19 cells were seeded into 96-well plates at 1.5 × 10^4^ cells/well and incubated for 24 h. The cells were then pretreated for 24 h with vehicle, lutein (17.1 μg/mL), cordylutene A (1; 1, 0.5, and 0.1 μg/mL), or CCL (10, 5, and 1 μg/mL). The concentrations of cordylutene A (**1**) were selected from the noncytotoxic range established in the ARPE-19 viability assay and were intended to assess protective effects at low concentrations (Table S1). After pretreatment, the normal control group received medium containing vehicle only, whereas the model control, positive control, and treatment groups received medium containing 10 μM *N*-retinylidene-*N*-retinylethanolamine (A2E; MedChemExpress, Cat. No. HY-134928) for 24 h. Following A2E incubation, cells were exposed to LED blue light (420–439 nm) for 9 min to induce photodamage. The irradiation time was selected based on preliminary optimization experiments to provide a reproducible but nonmaximal injury response in A2E-loaded ARPE-19 cells (Table S2). Lutein (17.1 μg/mL) was used as a positive control according to related A2E-laden ARPE-19 blue light injury models reported in the literature.^23^ The medium was then replaced with fresh culture medium, and the cells were allowed to recover for an additional 24 h. Cell viability was subsequently evaluated by the MTT assay as described above.

### Intracellular Oxidative Stress Assay

For the oxidative stress evaluation, ARPE-19 cells were seeded into 24-well plates at 1 mL per well (9.0 × 10^4^ cells/well) and incubated for 24 h. Treatment with the treatment solutions was conducted for 24 h. The medium was then replaced, the control received medium with 0.05% DMSO, and all other groups received medium containing 10 μM A2E (with 0.05% DMSO) for 24 h. After A2E treatment, cells were irradiated with the LED blue light (420–439 nm) for 9 min, and the medium was replaced with fresh culture medium. After a further 24 h incubation, cells were collected and stained according to the DCFDA/H2DCFDA assay protocol (Abcam). The staining was carried out at 37 °C for 30 min in the dark, and the fluorescence intensity was subsequently analyzed using flow cytometry (FACSLyric Clinical Flow Cytometry, BD) to assess intracellular oxidative stress levels.

### Statistical Analysis

All data are presented as mean ± standard deviation (SD). Statistical differences among groups were determined using one-way analysis of variance (ANOVA) followed by Duncan’s multiple range test, with *p* < 0.05 considered statistically significant.

## Supplementary Material







## Data Availability

Raw NMR data for compounds **1** and **2** are provided as Supporting Information files: compound1_rawNMR.zip and compound2_rawNMR.zip.

## Notes

compound**1**_rawNMR.zip (ZIP)
compound**2**_rawNMR.zip (ZIP)
(NMR (^1^H, ^13^C, DEPT, COSY, HSQC, HMBC, and H2BC), IR, UV, HRESIMS, and HRESIMS/MS spectra of compounds **1**–**2**; UV and HRESIMS/MS spectra of **3**–**10**; preliminary concentration screening of cordylutene A in the A2E/blue light–challenged ARPE-19 model (Table S1); and preliminary optimization of blue light exposure time in A2E-loaded ARPE-19 cells (Table S2)). compound1_rawNMR.zip (Raw NMR data for compound **1**) compound2_rawNMR.zip (Raw NMR data for compound **2**) (PDF)
