# Supplementary material for: Utilize Metabolomics Molecular Networking to Guide the Discovery of Chemical Entities from Cordyceps militaris Against Blue Light Hazards
Source: J Nat Prod. 2026 Jul 9;89(8):2265–74. doi: 10.1021/acs.jnatprod.6c00427 (PMC13532171; doi:10.1021/acs.jnatprod.6c00427)
Supplement: Supplementary file 1 [file np6c00427_si_003.pdf]

Supplementary information

Utilize metabolomics molecular networking to guide the discovery of chemical entities from *Cordyceps militaris* against blue light hazards

Ho-Cheng Wu [1,2,3], Andrea Gu [1,4], Ya-Lin Chang [5], Chung-Kuang Lu [6], Yi-Chieh Lee [7], Po-Wei Yu [5,] and Tzong-Huei Lee [4,*]*

[1] School of Pharmacy, College of Pharmacy, Kaohsiung Medical University, Kaohsiung 807378, Taiwan

[2] Department of Medical Research, Kaohsiung Medical University Hospital, Kaohsiung 807378, Taiwan

[3] Drug Development and Value Creation Research Center, Kaohsiung Medical University, Kaohsiung 807378, Taiwan

[4] Institute of Fisheries Science, College of Life Science, National Taiwan University, Taipei 106319, Taiwan

[5] JoinCaring Co., Ltd., Taipei 100024, Taiwan

[6] National Research Institute of Chinese Medicine, Taipei 112026, Taiwan

[7] Biomedical Industry Ph.D. Program, National Yang Ming Chiao Tung University, Taipei 112304, Taiwan

[*] Corresponding Authors

AUTHOR INFORMATION

Corresponding Author

Tzong-Huei Lee − Institute of Fisheries Science, College of Life Science, National Taiwan University, Taipei 106319, Taiwan; Phone: +886 2 33661828; E-mail: thlee1@ntu.edu.tw

Po-Wei Yu − *JoinCaring Co., Ltd., Taipei 100024, Taiwan*; E-mail: pwy@accurome.com

Appendix A. Supplementary data:

Figure S1. ^{1}H NMR (800 MHz, DMSO-d_6) spectrum of compound **1**. 1

Figure S2. ^{13}C NMR (200 MHz, DMSO-d_6) spectrum of compound **1**. 1

Figure S3. DEPT spectrum of compound **1**............ 2

Figure S4. COSY spectrum of compound **1**............ 2

Figure S5. HSQC spectrum of compound **1**............ 3

Figure S6. HMBC spectrum of compound **1**............ 3

Figure S7. H2BC spectrum of compound **1**. 4

Figure S8. IR spectrum of compound **1**............ 4

Figure S9. UV spectrum of compound **1**............ 5

Figure S10. HRESIMS spectrum of compound **1**. 5

Figure S11. HRESIMS/MS spectrum of compound **1** (isolated). 6

Figure S12. HRESIMS/MS spectrum of compound **1**. 6

Figure S13. ^{1}H NMR (800 MHz, CD_3OD) spectrum of compound **2**............ 7

Figure S14. ^{13}C NMR (200 MHz, CD_3OD) spectrum of compound **2**............ 7

Figure S15. DEPT spectrum of compound **2**............ 8

Figure S16. COSY spectrum of compound **2**............ 8

Figure S17. HSQC spectrum of compound **2**............ 9

Figure S18. HMBC spectrum of compound **2**............ 9

Figure S19. H2BC spectrum of compound **2**. 10

Figure S20. IR spectrum of compound **2**............ 10

Figure S21. UV spectrum of compound **2**............ 11

Figure S22. HRESIMS spectrum of compound **2**. 11

Figure S23. HRESIMS/MS spectrum of compound **2** (isolated). 12

Figure S24. HRESIMS/MS spectrum of compound **2**. 12

Figure S25. UV spectrum of compound **3**............ 13

Figure S26. HRESIMS/MS spectrum of compound **3**. 13

Figure S27. UV spectrum of compound **4**............ 14

Figure S28. HRESIMS/MS spectrum of compound **4**. 14

Figure S29. UV spectrum of compound **5**............ 15

Figure S30. HRESIMS/MS spectrum of compound **5**. 15

Figure S31. UV spectrum of compound **6**. 16

Figure S32. HRESIMS/MS spectrum of compound **6**. 16

Figure S33. UV spectrum of compound **7**. 17

Figure S34. HRESIMS/MS spectrum of compound **7**. 17

Figure S35. UV spectrum of compound **8**. 18

Figure S36. HRESIMS/MS spectrum of compound **8**. 18

Figure S37. UV spectrum of compound **9**. 19

Figure S38. HRESIMS/MS spectrum of compound **9**. 19

Figure S39. UV spectrum of compound **10**. 20

Figure S40. HRESIMS/MS spectrum of compound **10**. 20

Table S1. Preliminary concentration screening of cordylutene A (**1**) in the A2E/blue light–challenged ARPE-19 model 21

Table S2. Preliminary optimization of blue light exposure time in A2E-loaded ARPE-19 cells 21

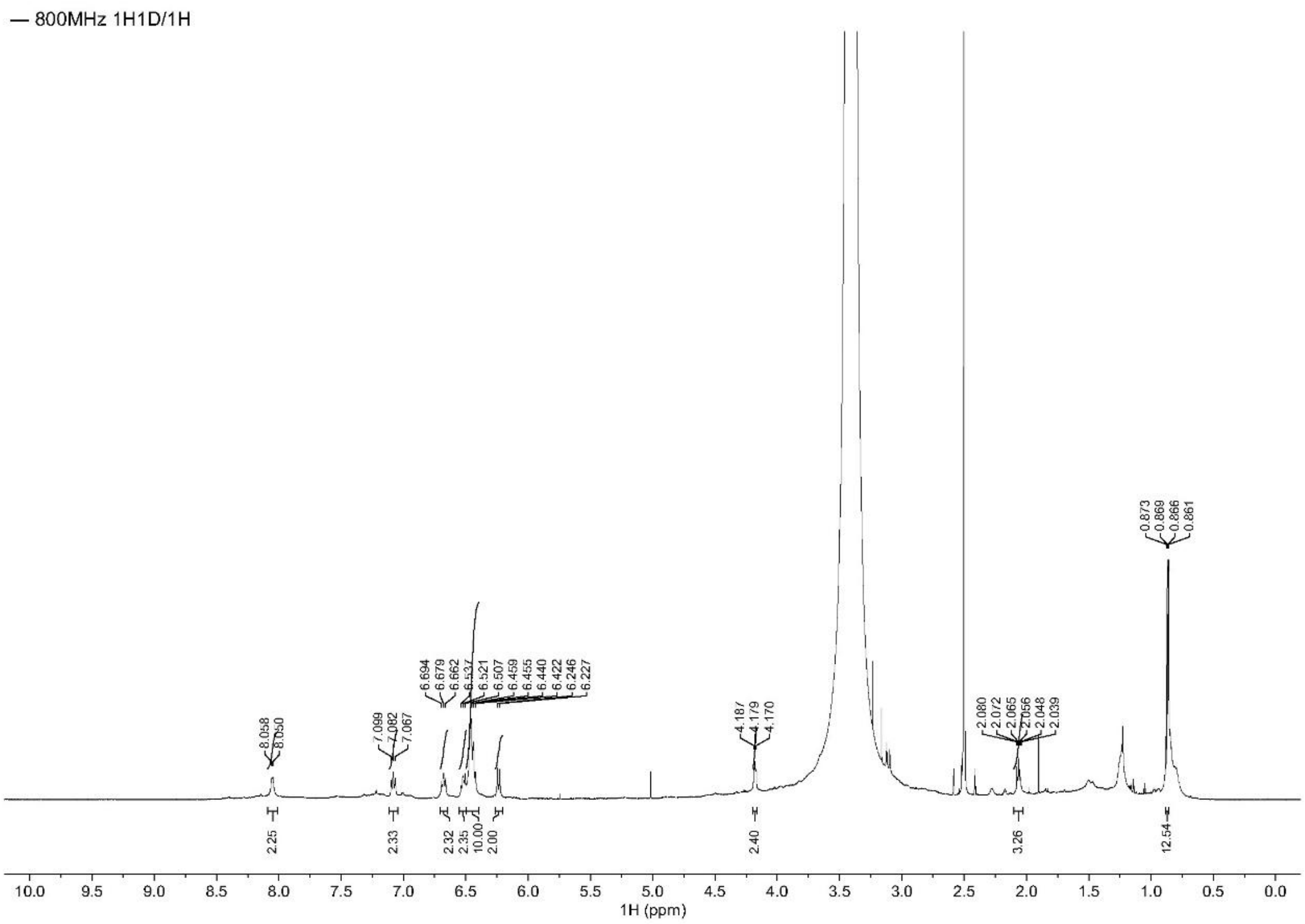

Figure S1. ^{1}H NMR (800 MHz, DMSO-d_6) spectrum of compound **1**.

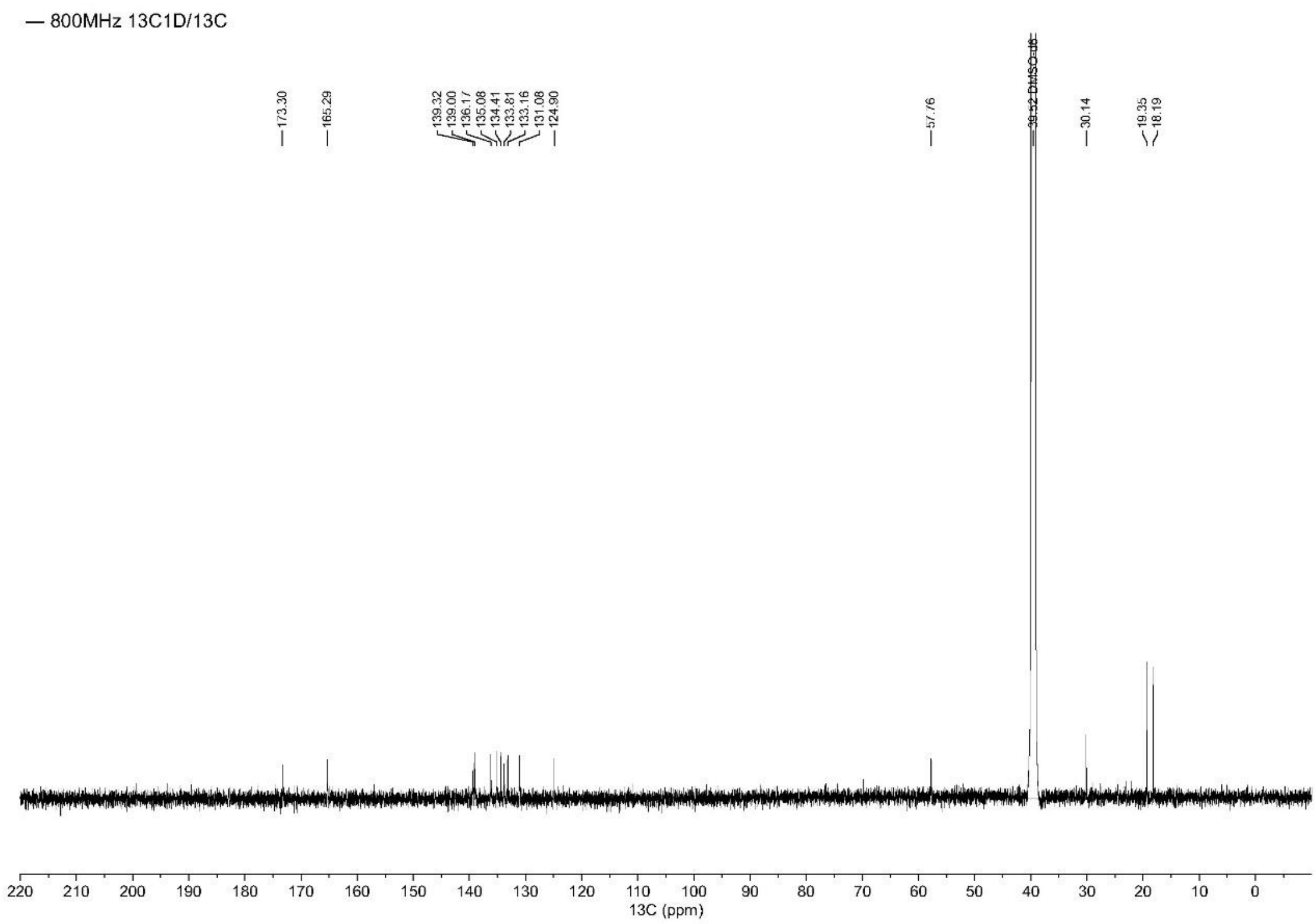

Figure S2. ^{13}C NMR (200 MHz, DMSO-d_6) spectrum of compound **1**.

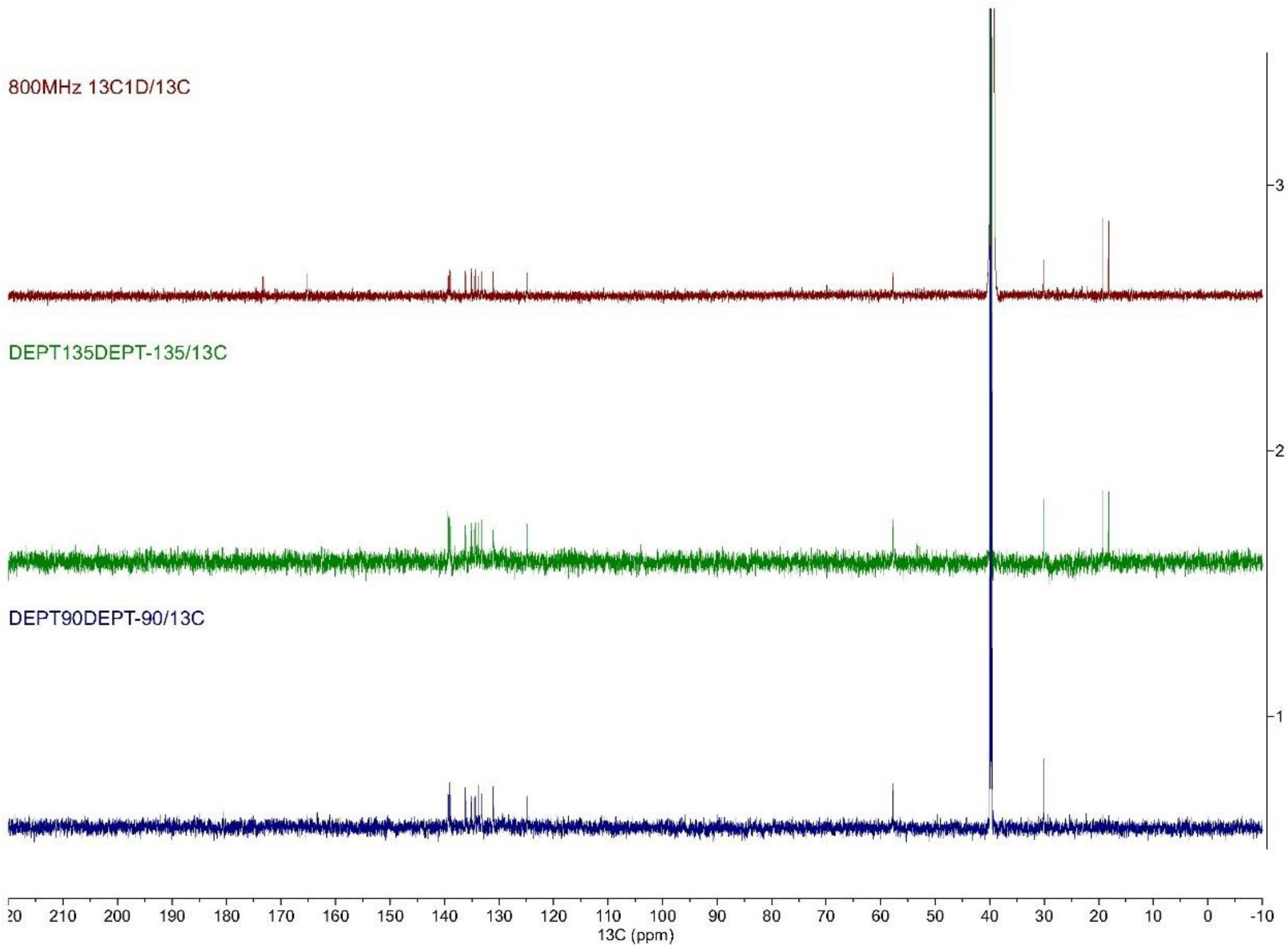

Figure S3. DEPT spectrum of compound **1**.

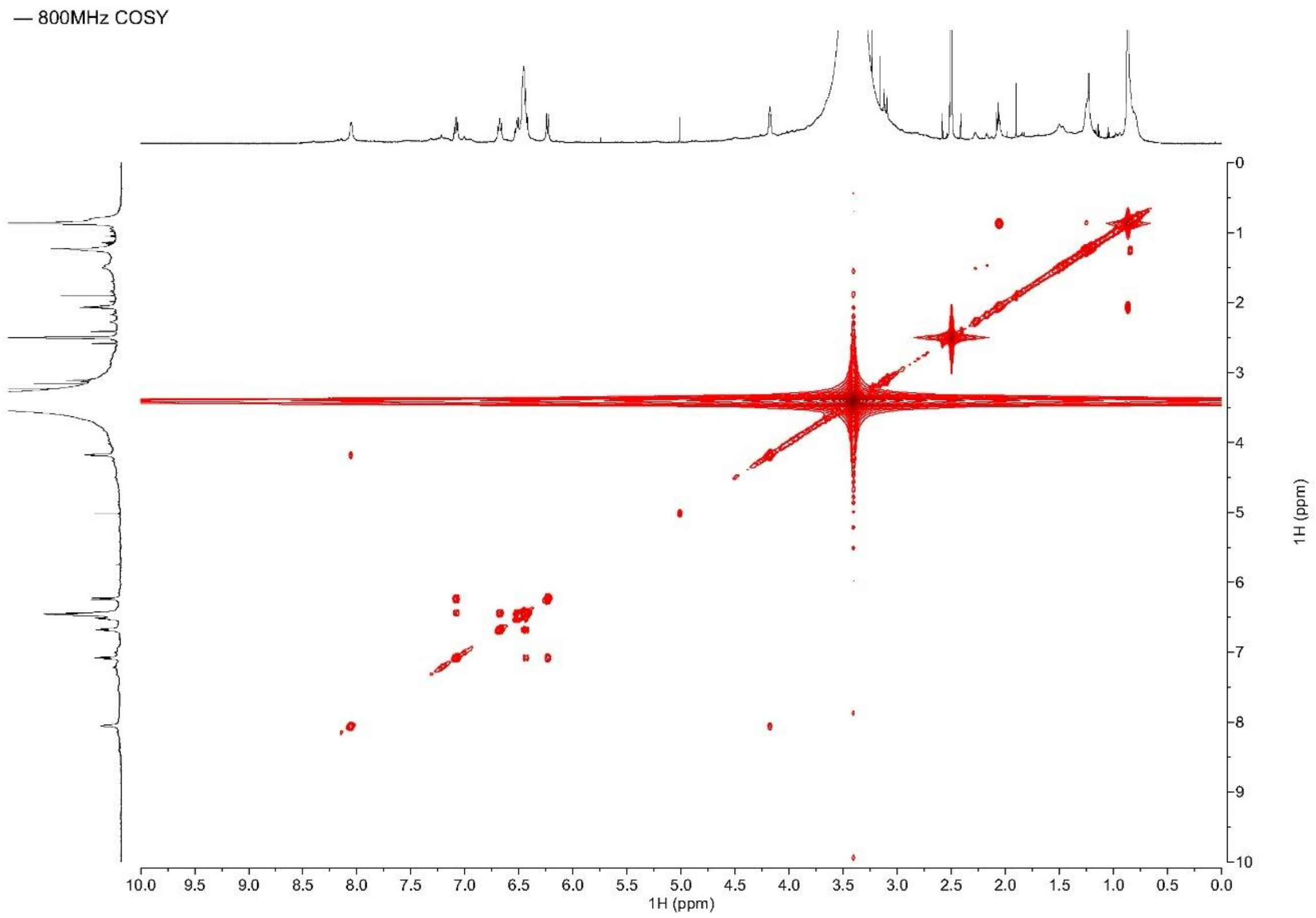

Figure S4. COSY spectrum of compound **1**.

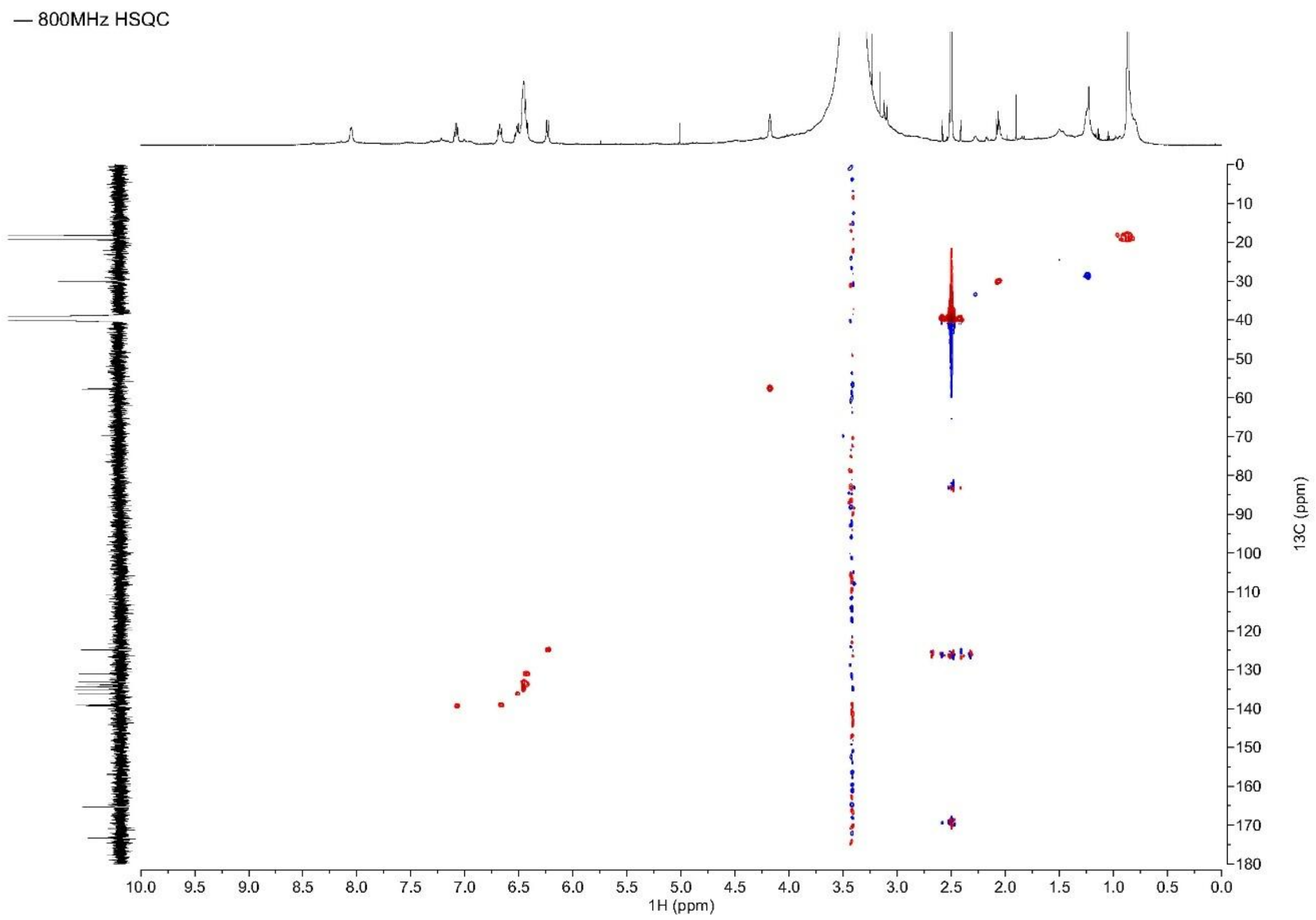

Figure S5. HSQC spectrum of compound **1**.

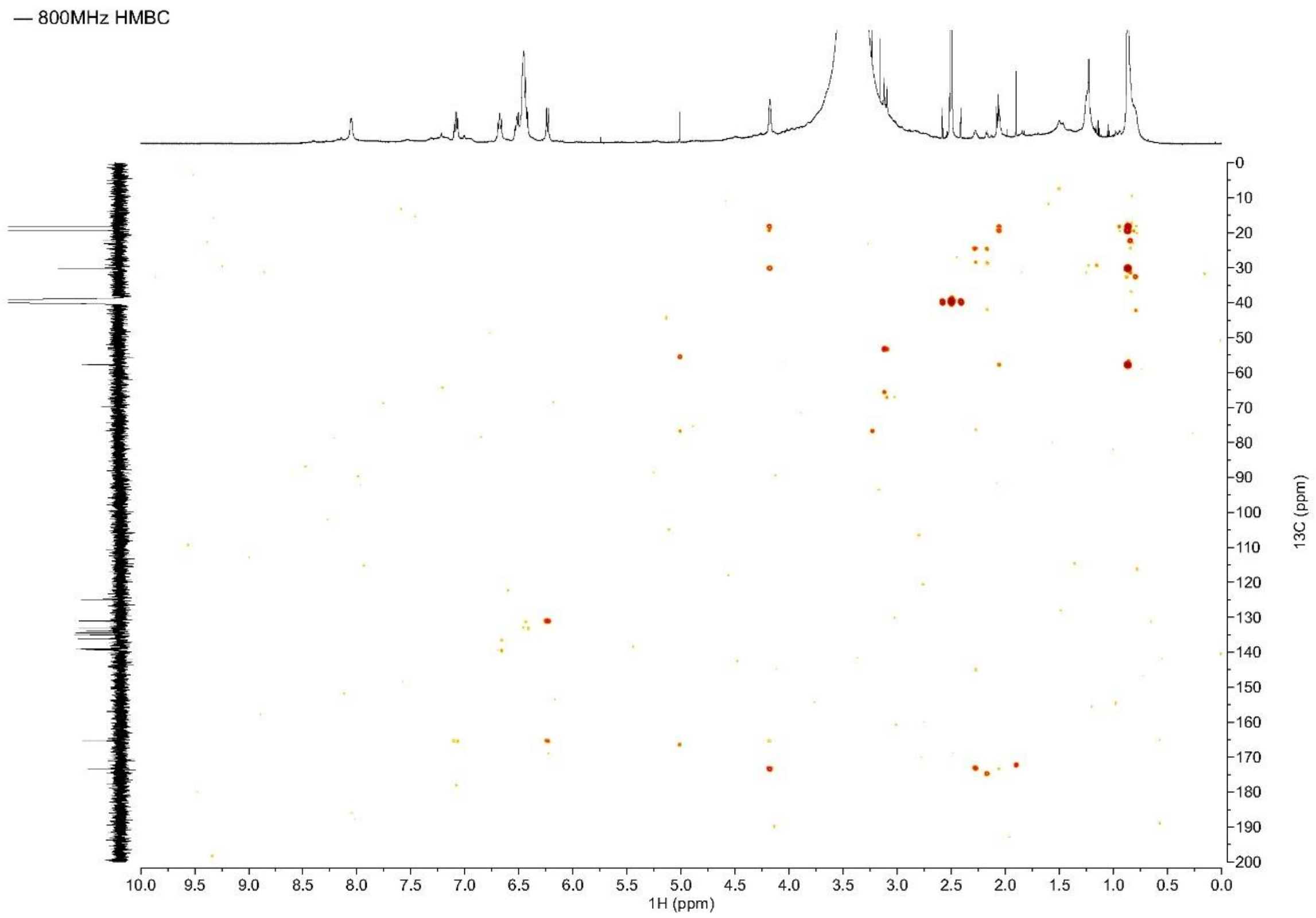

Figure S6. HMBC spectrum of compound **1**.

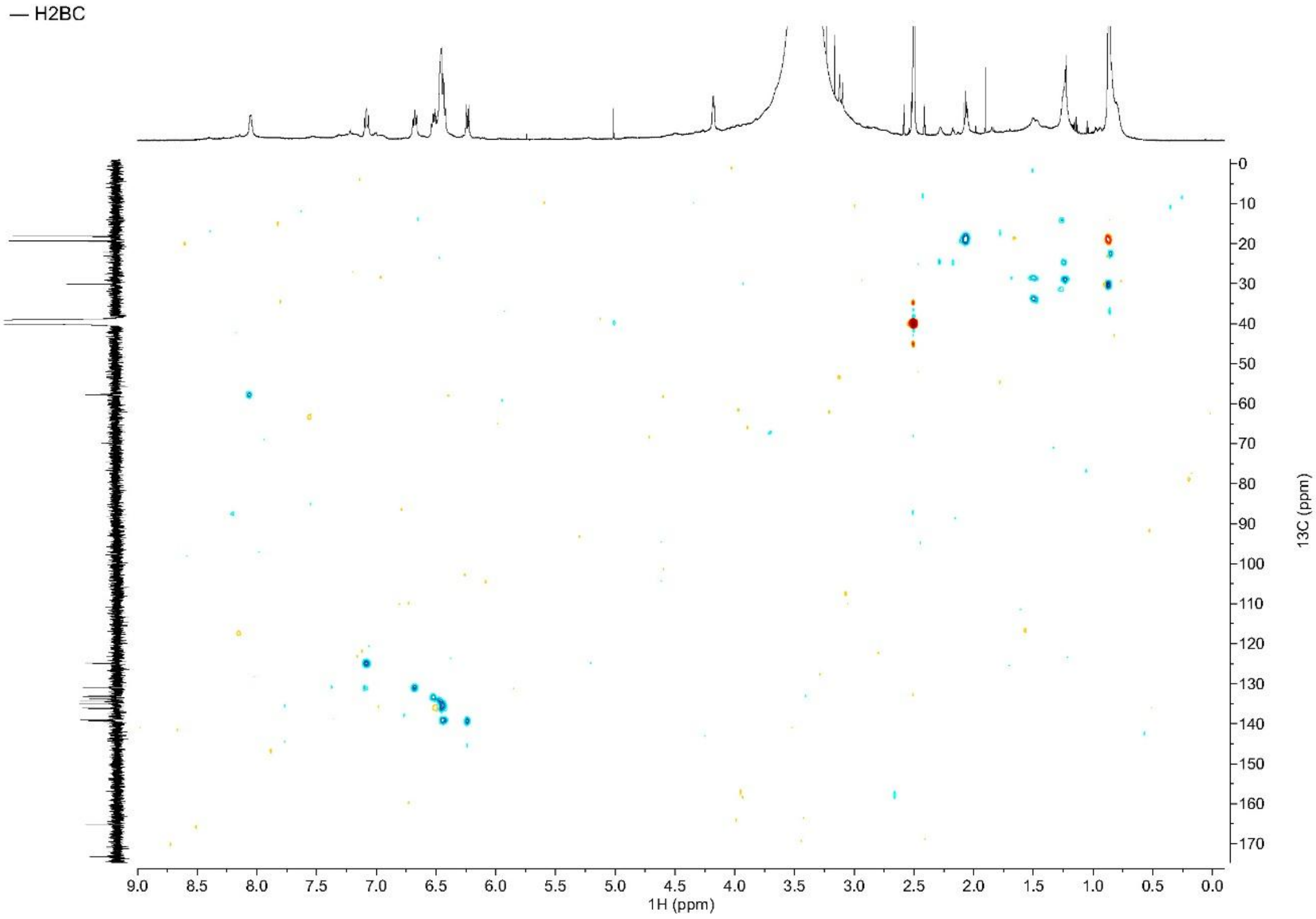

Figure S7. H2BC spectrum of compound **1**.

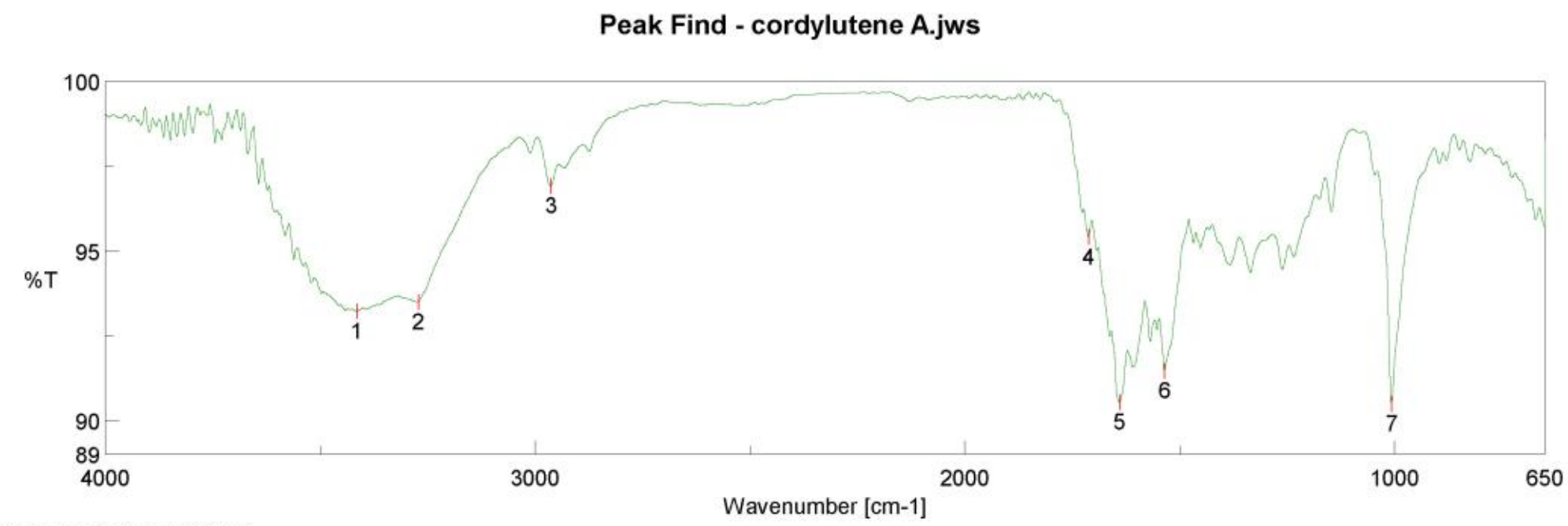

[Result of Peak Picking]

No.	Position	Intensity	No.	Position	Intensity	No.	Position	Intensity
1	3415.31	93.2145	2	3271.64	93.4931	3	2963.09	96.9189
4	1711.51	95.4103	5	1640.16	90.5275	6	1535.06	91.4504
7	1006.66	90.4881						

Figure S8. IR spectrum of compound **1**.

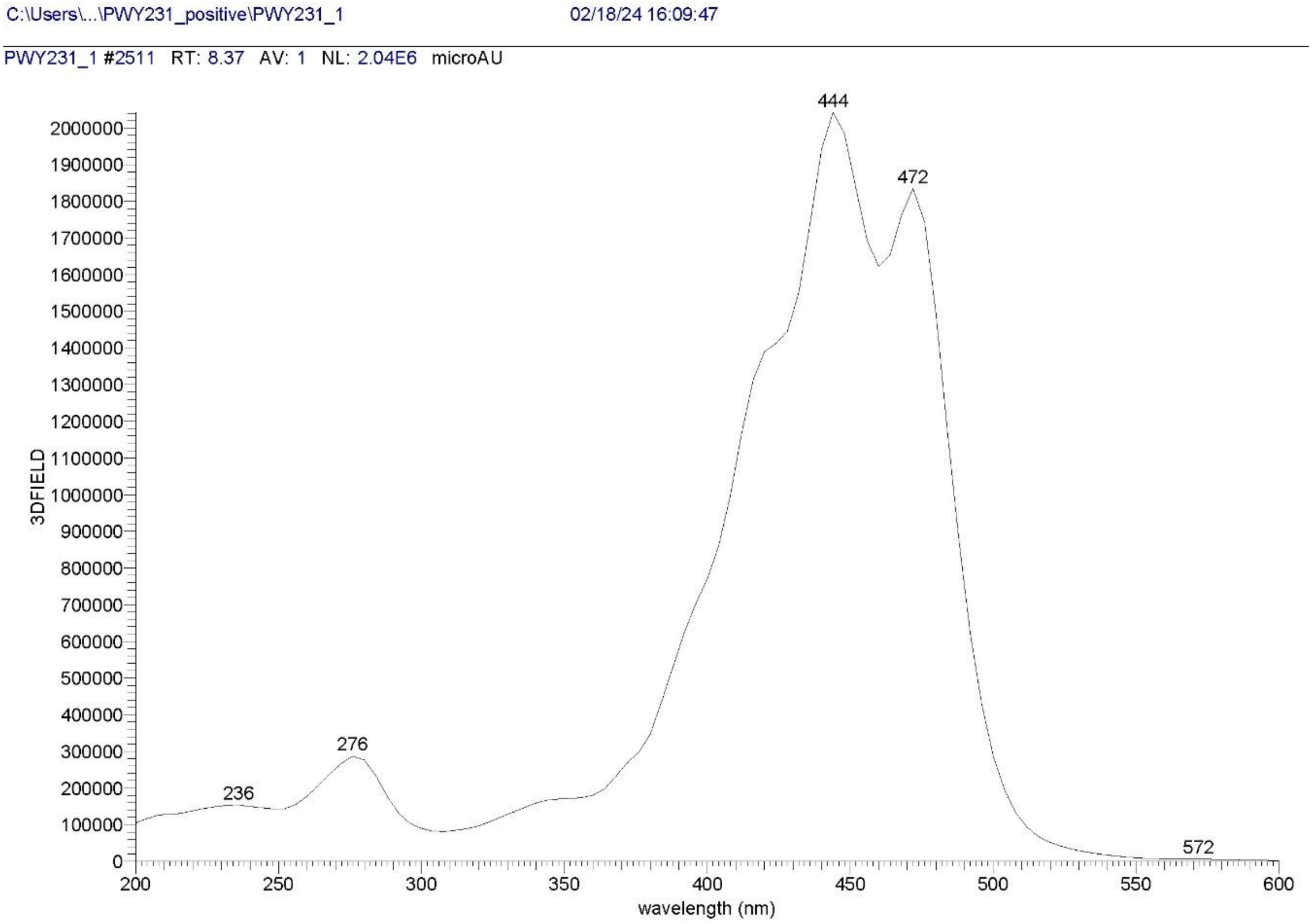

Figure S9. UV spectrum of compound **1**.

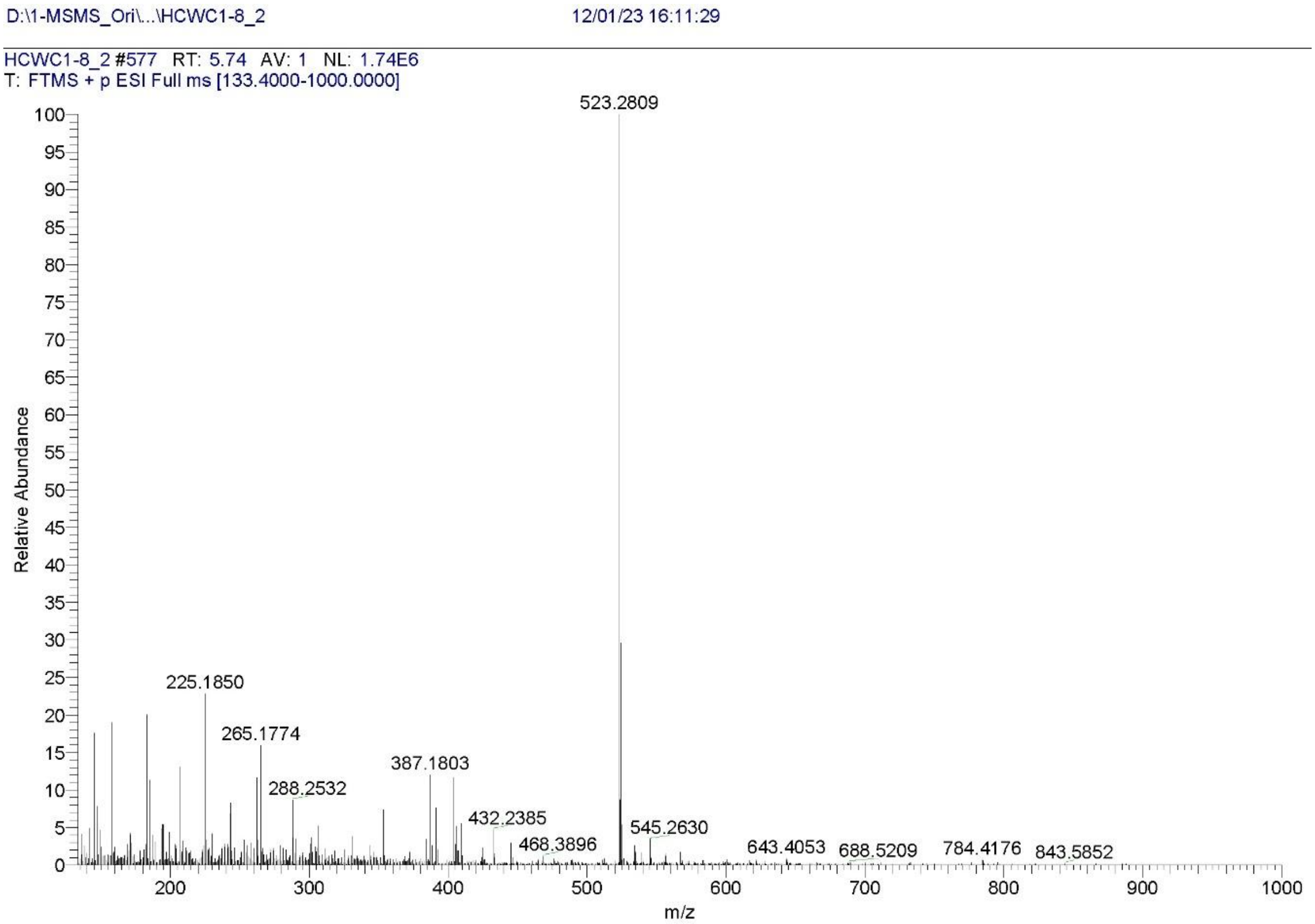

Figure S10. HRESIMS spectrum of compound **1**.

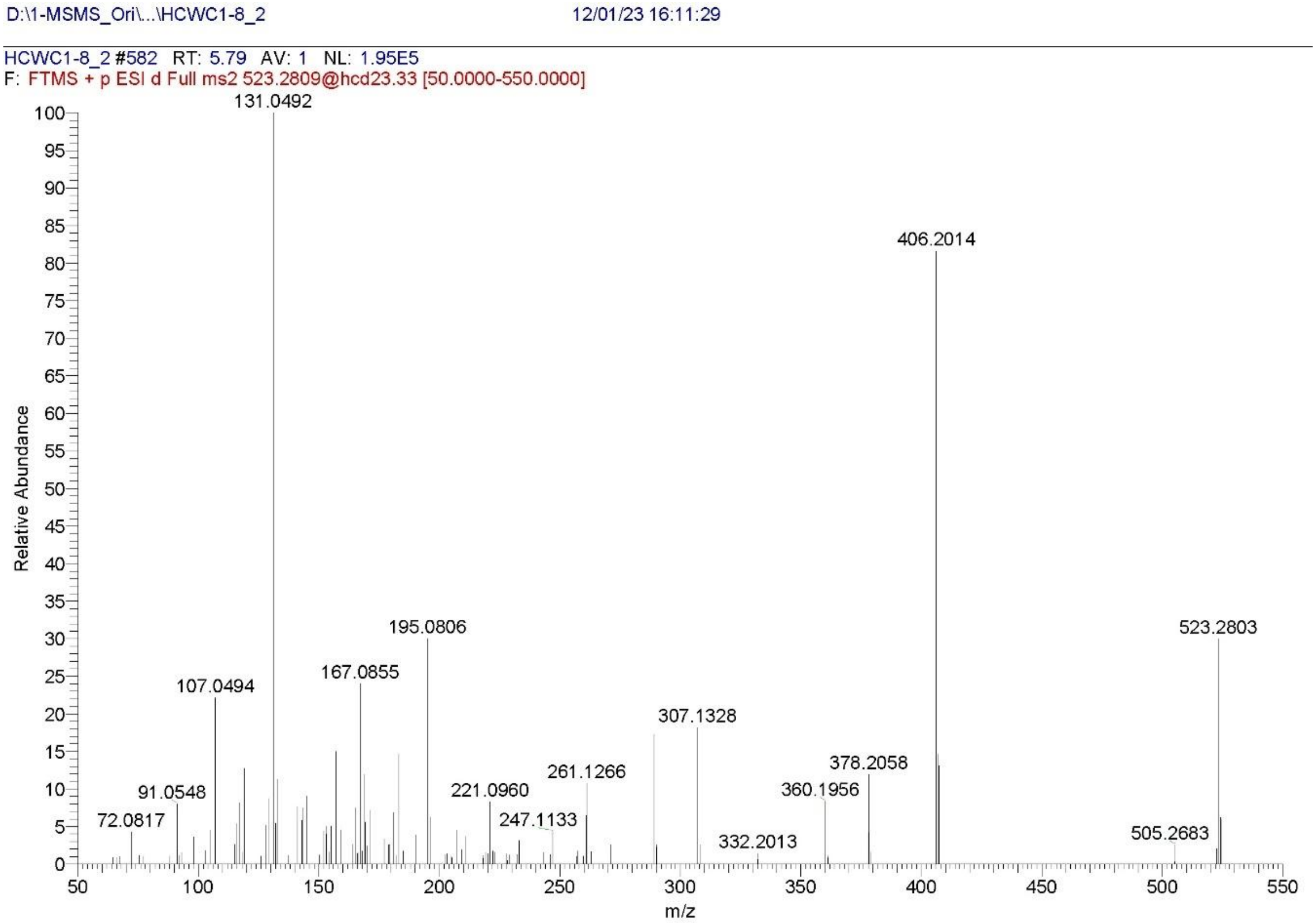

Figure S11. HRESIMS/MS spectrum of compound **1** (isolated).

Compound **1** Precursor *m/z*: 523.2799 Shared name: 1224

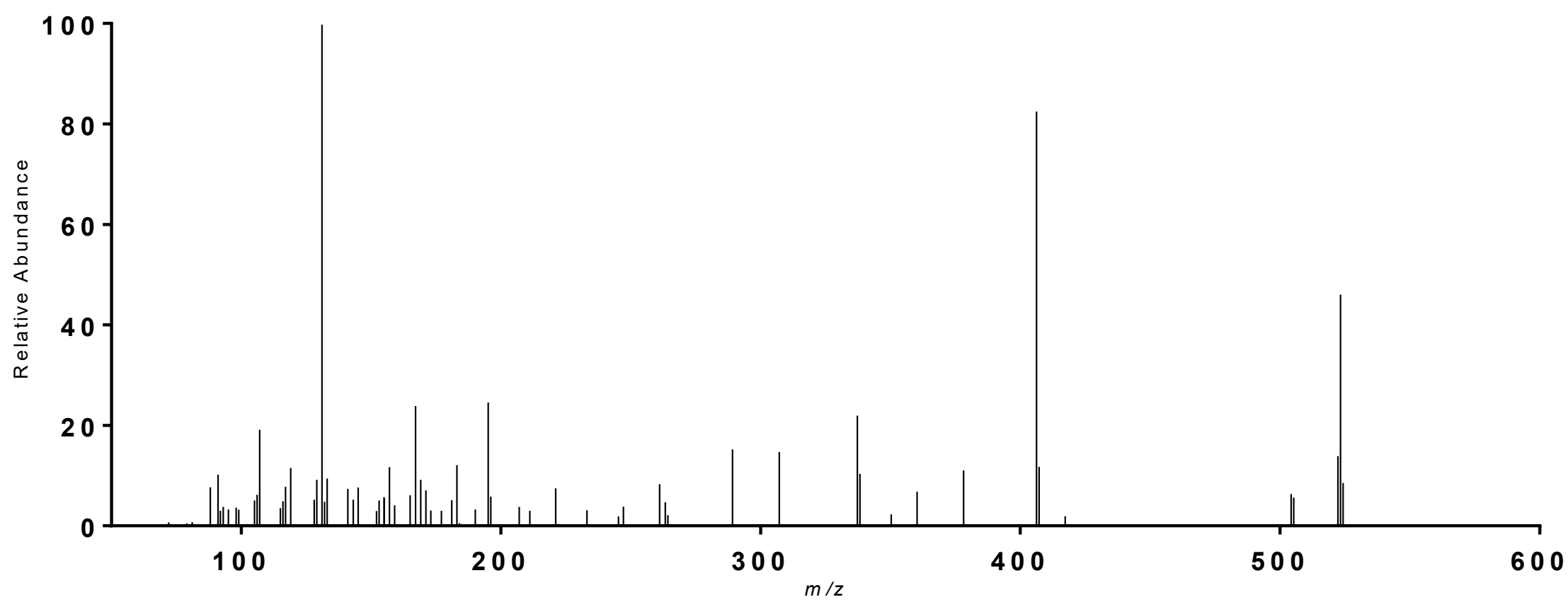

m/z	Relative Abundance	*m/z*	Relative Abundance	*m/z*	Relative Abundance	*m/z*	Relative Abundance	*m/z*	Relative Abundance
67.05501	0.310385	115.05460	3.512415	153.06990	5.031469	184.07350	0.561495	289.12250	15.242320
72.08157	0.683413	116.07100	4.915509	155.01050	5.702733	190.12330	3.273414	307.13330	14.725510
79.05465	0.505003	117.07030	7.790572	155.08610	5.437910	195.08090	24.579710	337.27440	21.926310
81.07044	0.740621	119.04960	11.499650	157.06510	11.681680	196.08790	5.833313	338.27790	10.318710
83.08585	0.355138	128.06250	5.188804	159.08060	4.056391	207.08100	3.755288	350.26950	2.304439
88.03988	7.691553	129.07030	9.168244	165.07000	6.078177	211.07510	3.027558	360.19640	6.784434
91.05479	10.169870	131.04950	100.000000	167.08590	23.856380	221.09660	7.457329	378.20700	11.063770
91.98635	3.001647	131.05990	0.321991	168.09160	0.323859	222.10390	0.364715	406.20210	82.463680
93.07008	3.786041	131.08560	0.413006	169.06510	9.181062	233.09680	3.109718	407.20540	11.742090
95.08591	3.263108	132.05270	4.792228	169.10130	0.419012	245.22690	1.860351	417.24180	1.947327
98.06034	3.617992	133.06520	9.445092	171.08050	7.058668	247.11240	3.833254	504.27320	6.358902
98.98440	3.201286	141.07000	7.358508	173.02130	3.078291	261.12810	8.313155	505.27380	5.624538
105.07030	5.066868	143.08570	5.215720	177.07020	2.981356	263.23730	4.719699	522.28240	13.904230
106.05040	6.204309	145.06510	7.602895	181.06520	5.114539	264.24110	2.155960	523.28240	46.046670
107.04960	19.130360	152.06170	2.949682	183.08080	12.112330	271.11150	0.323901	524.28400	8.530264

Figure S12. HRESIMS/MS spectrum of compound **1**.

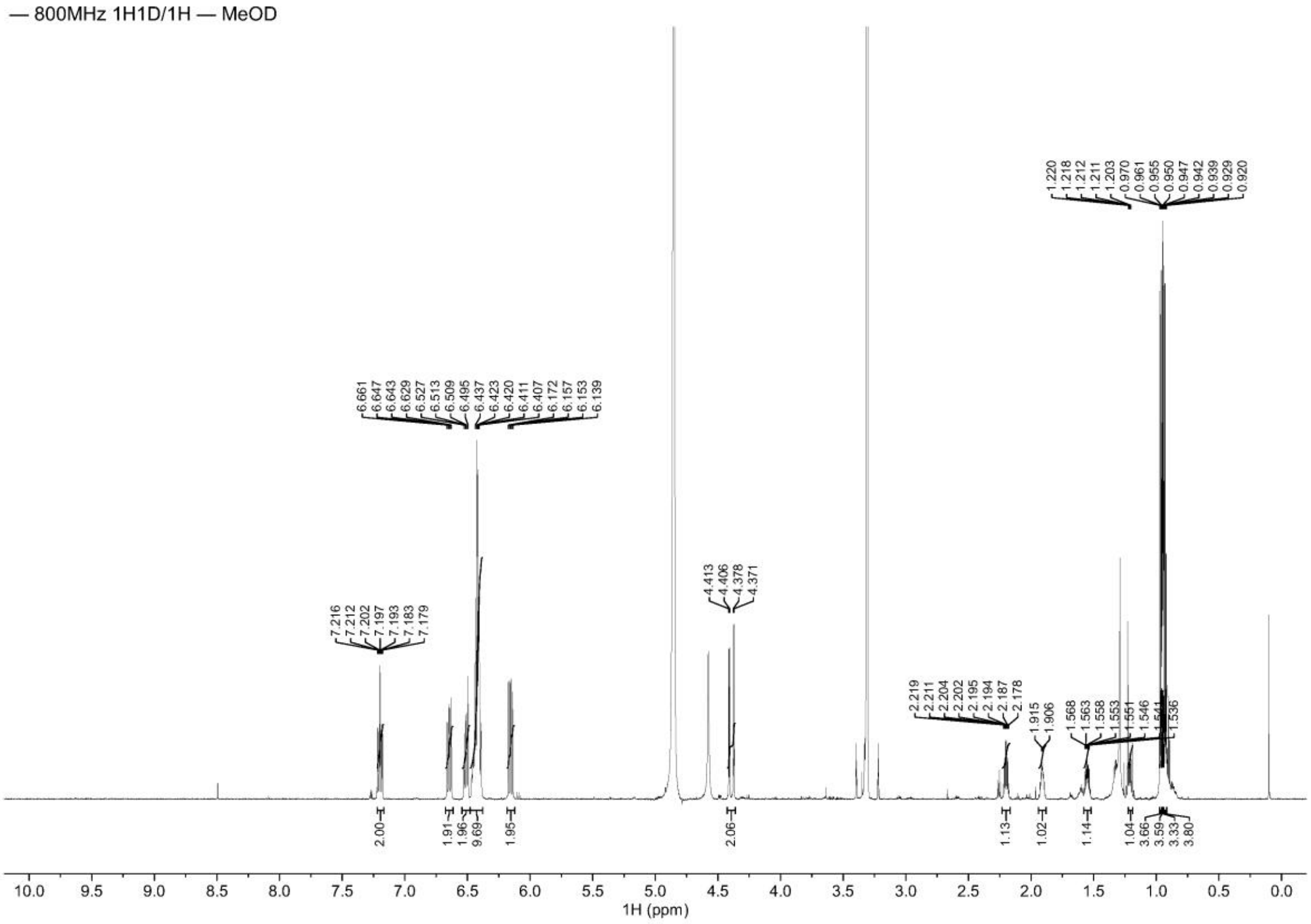

Figure S13. ^{1}H NMR (800 MHz, CD_3OD) spectrum of compound **2**.

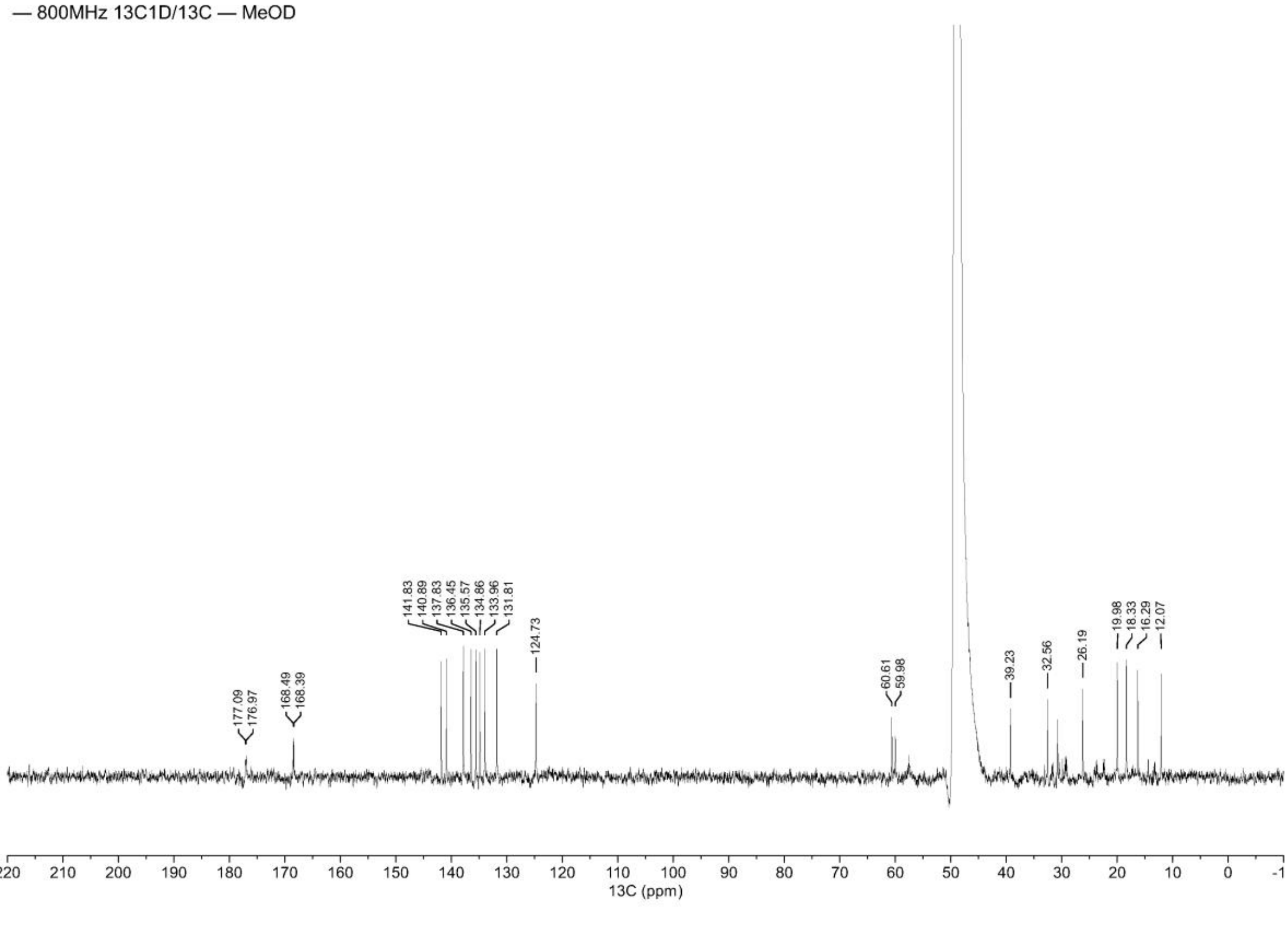

Figure S14. ^{13}C NMR (200 MHz, CD_3OD) spectrum of compound **2**.

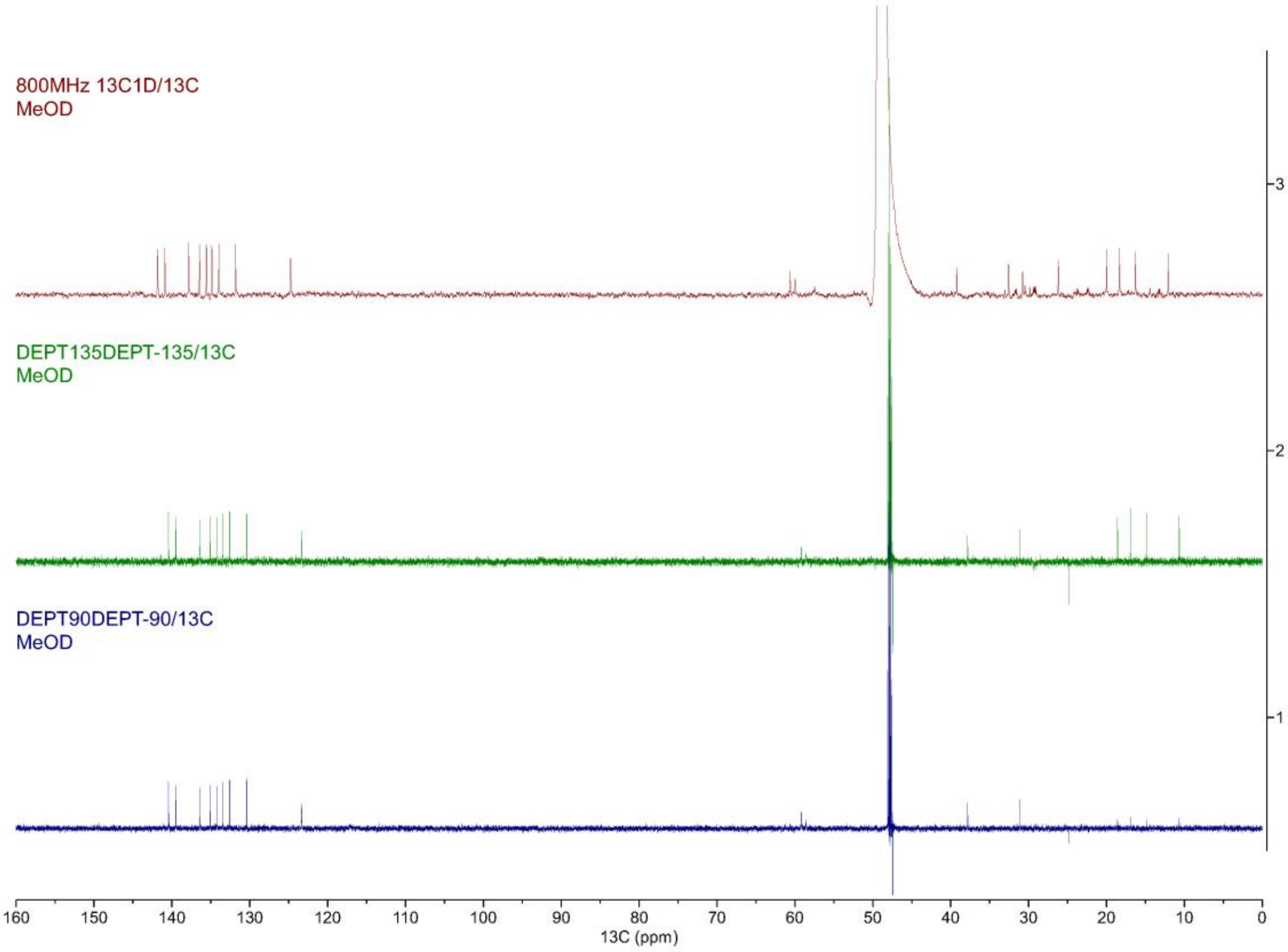

Figure S15. DEPT spectrum of compound **2**.

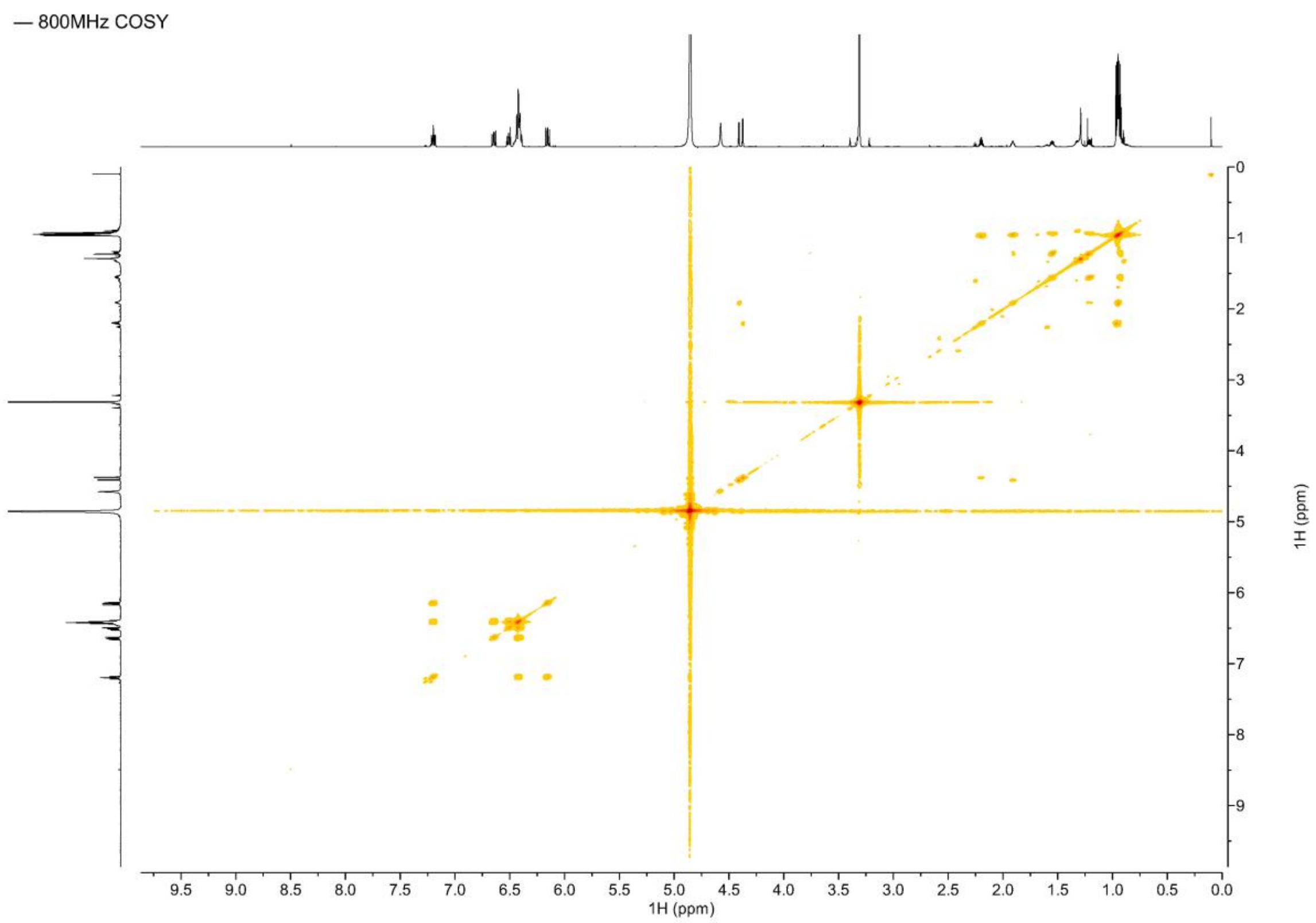

Figure S16. COSY spectrum of compound **2**.

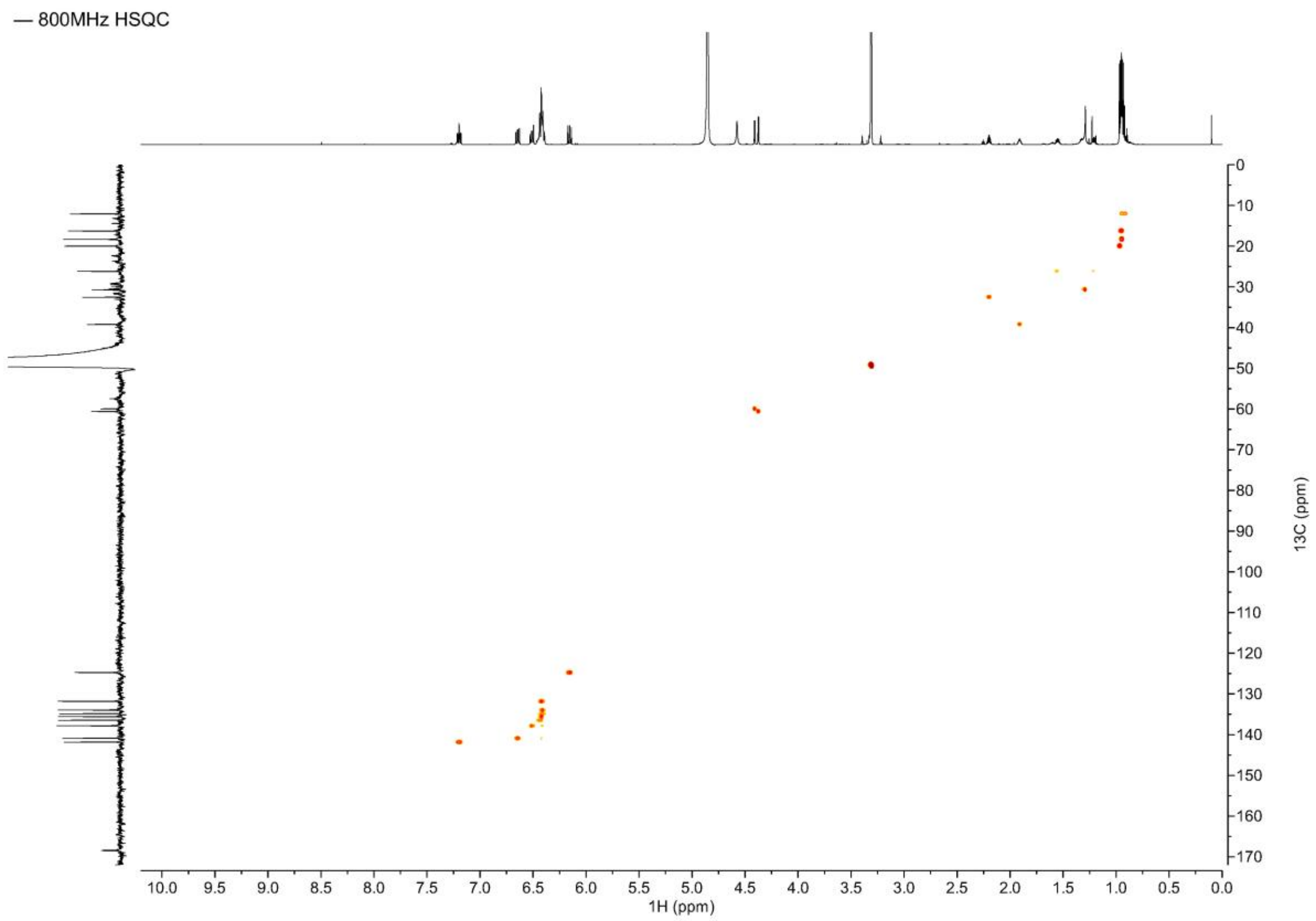

Figure S17. HSQC spectrum of compound **2**.

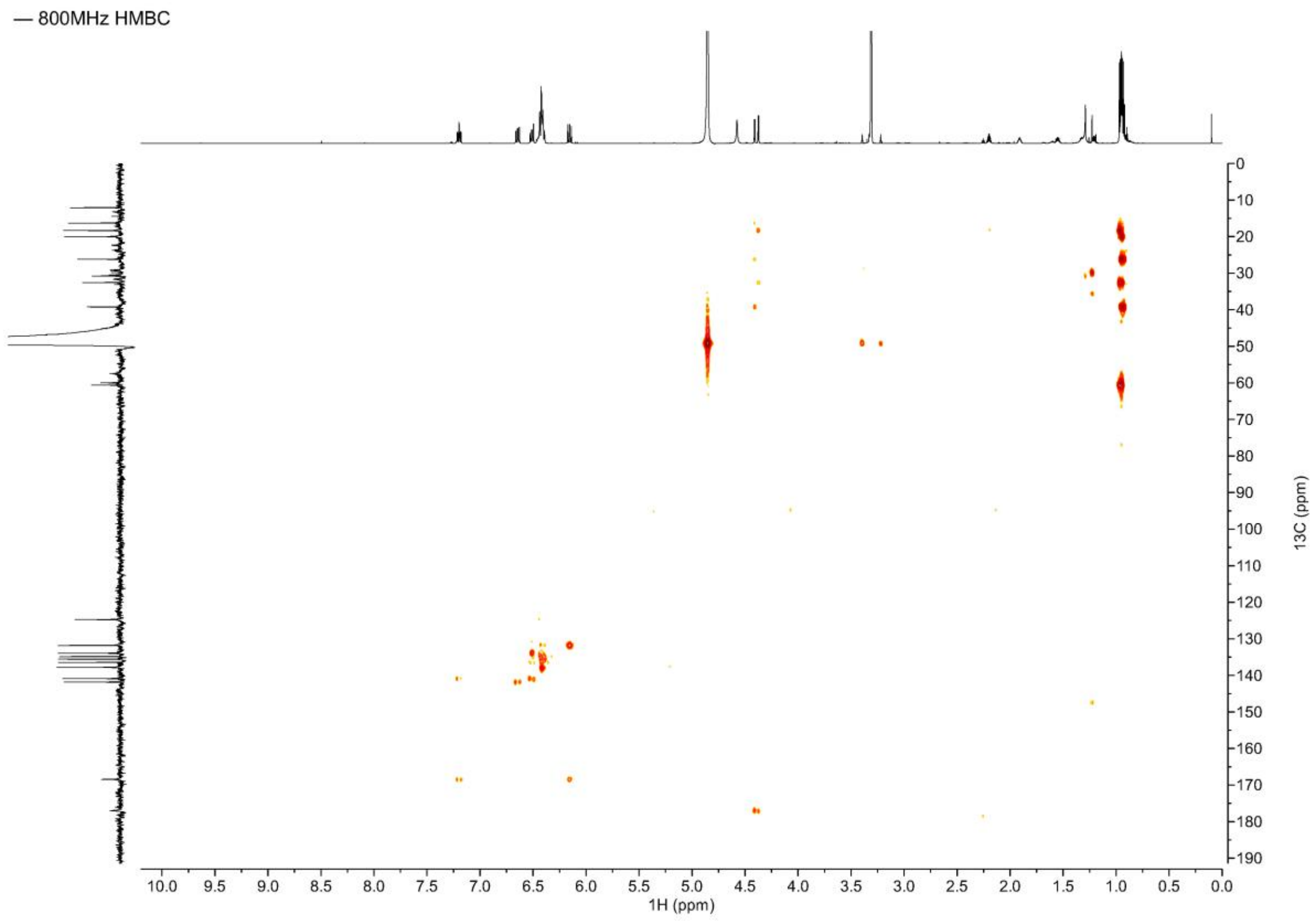

Figure S18. HMBC spectrum of compound **2**.

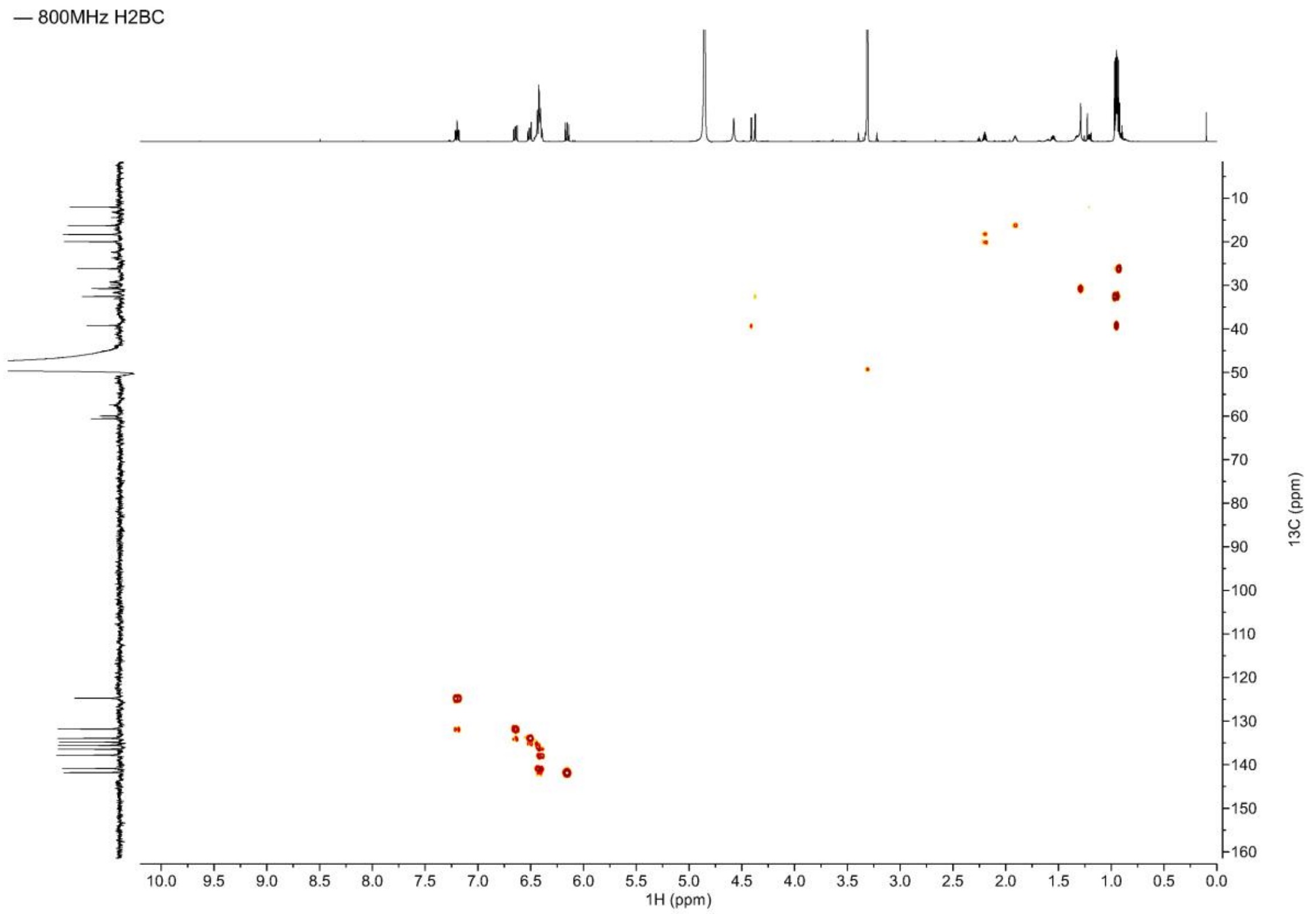

Figure S19. H2BC spectrum of compound **2**.

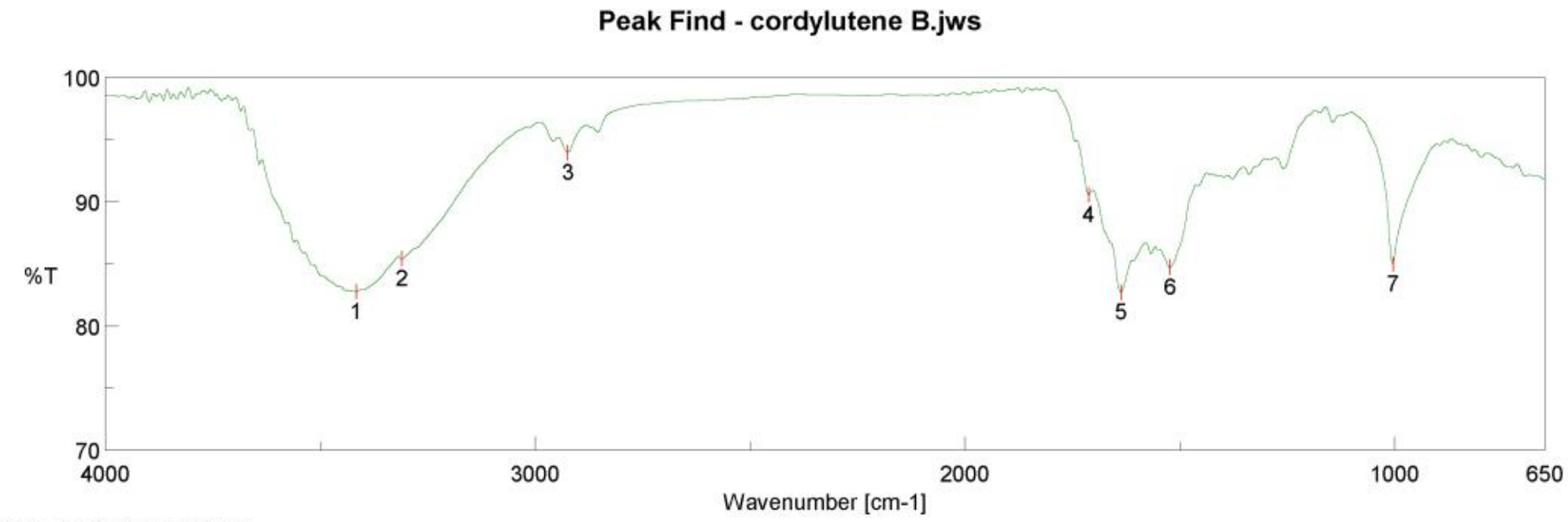

[Result of Peak Picking]

No.	Position	Intensity	No.	Position	Intensity	No.	Position	Intensity
1	3416.28	82.7599	2	3310.21	85.4007	3	2923.56	93.9677
4	1711.51	90.5752	5	1636.3	82.7112	6	1522.52	84.7051
7	1003.77	84.9654						

Figure S20. IR spectrum of compound **2**.

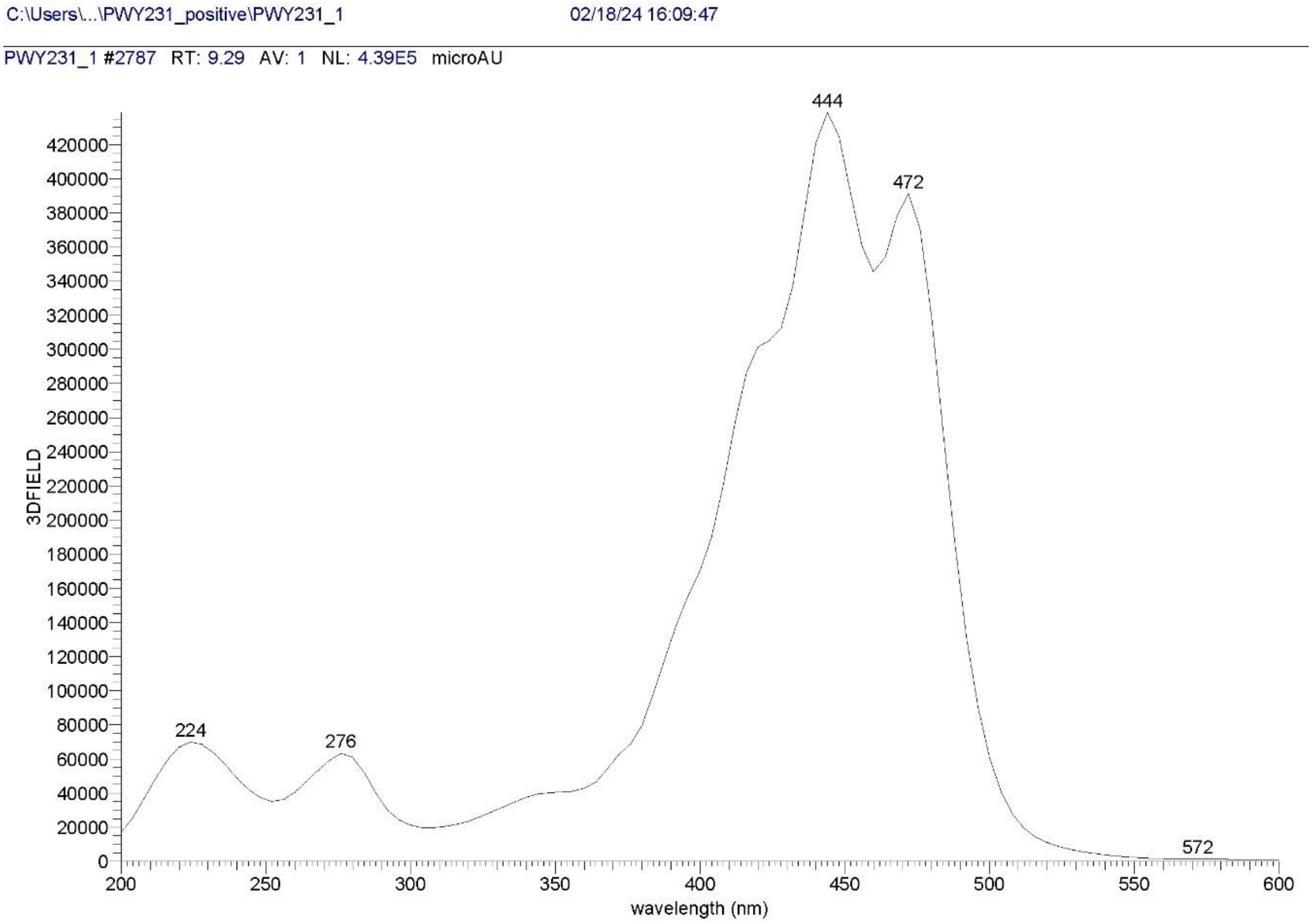

Figure S21. UV spectrum of compound **2**.

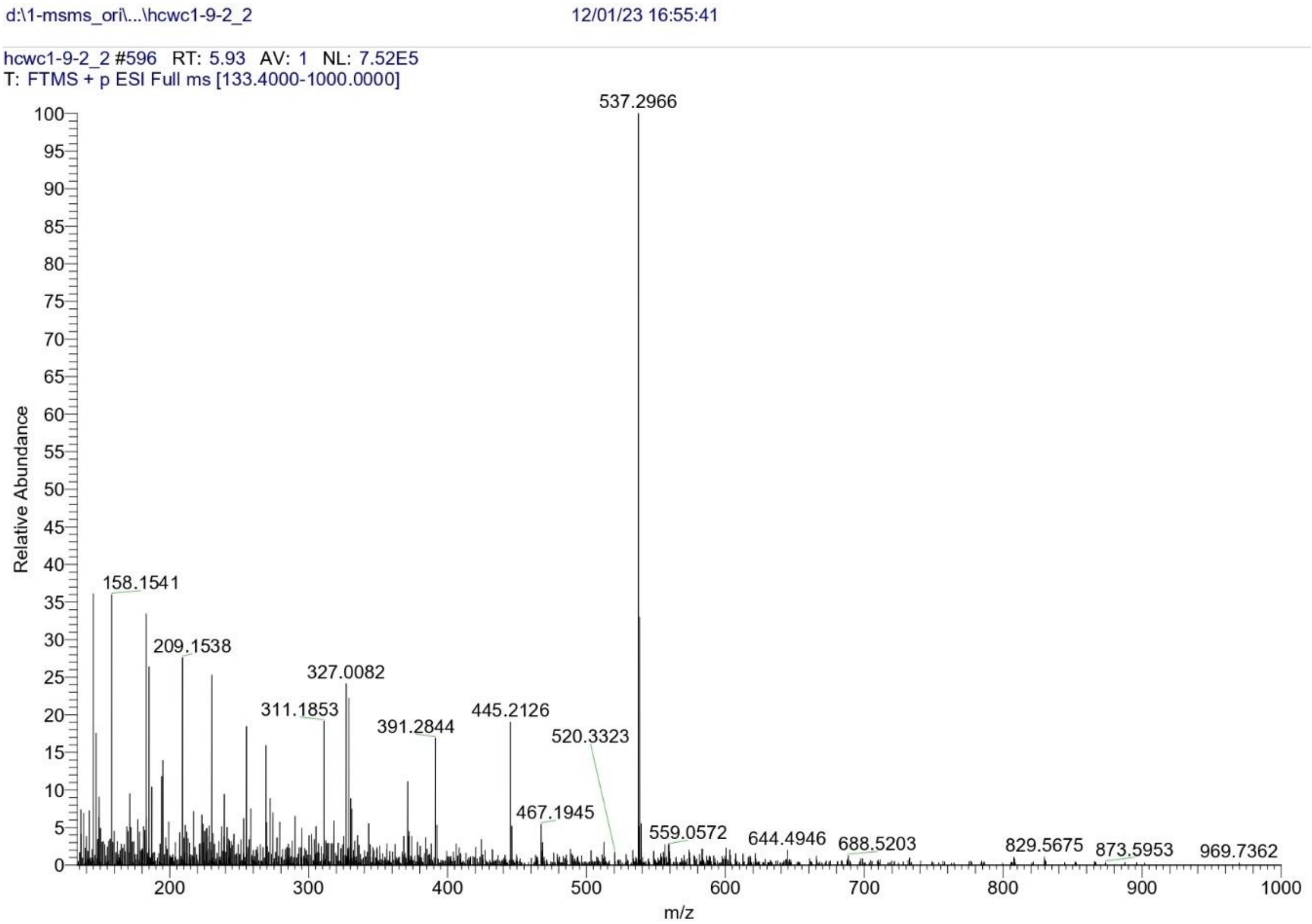

Figure S22. HRESIMS spectrum of compound **2**.

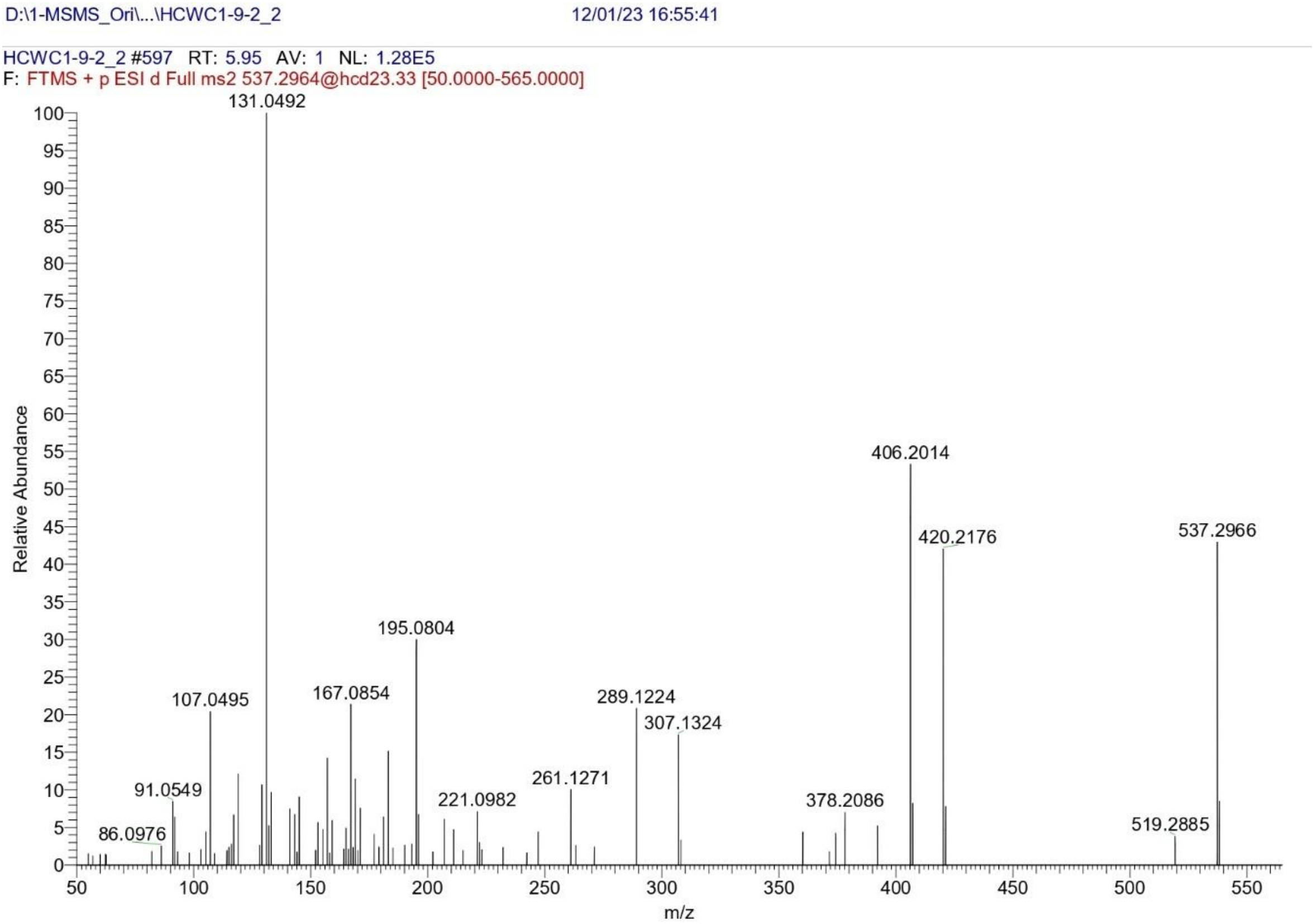

Figure S23. HRESIMS/MS spectrum of compound **2** (isolated).

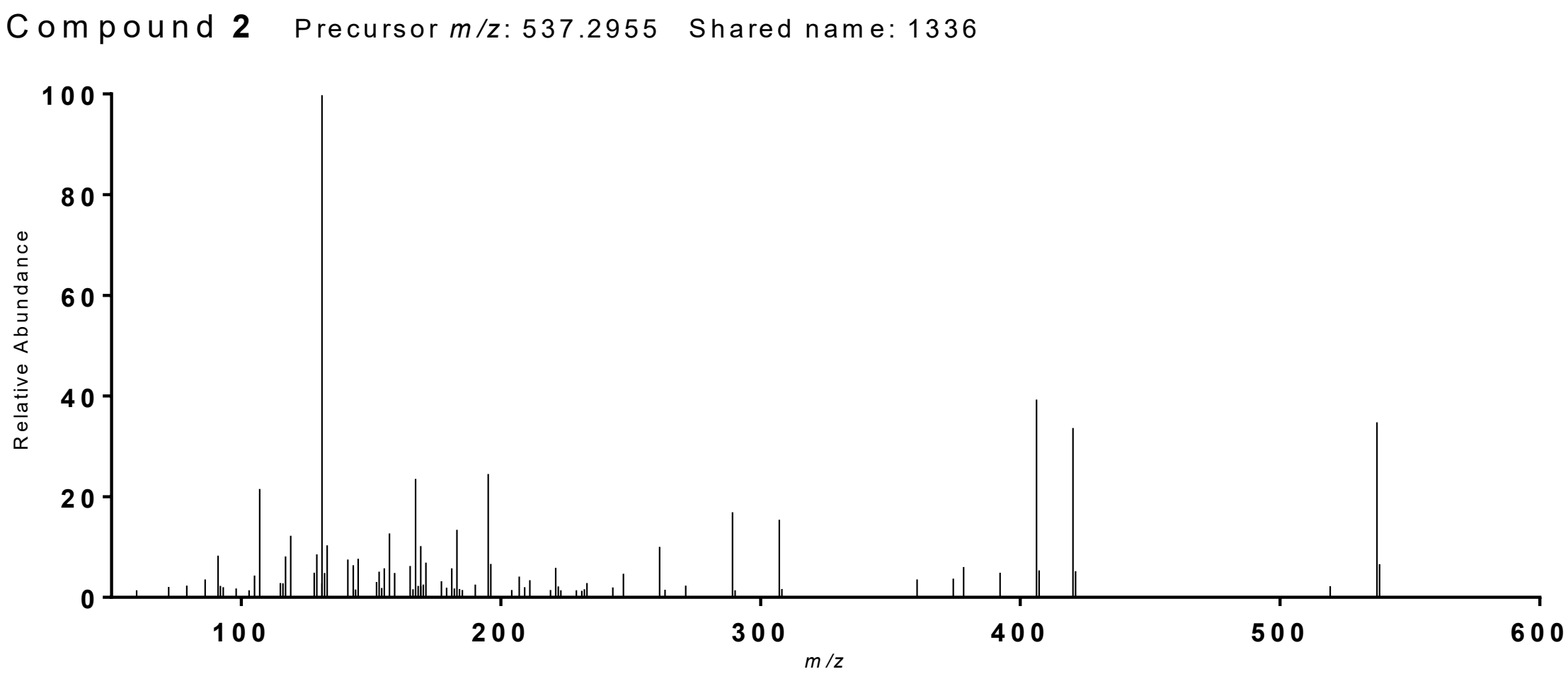

m/z	Relative Abundance	*m/z*	Relative Abundance	*m/z*	Relative Abundance	*m/z*	Relative Abundance	*m/z*	Relative Abundance	*m/z*	Relative Abundance
59.69989	1.427874	128.06240	4.885928	159.08070	4.844316	185.09640	1.452171	232.08860	1.615551	406.20200	39.288110
72.08142	2.062654	129.07030	8.562258	165.07010	6.211644	190.12300	2.521014	233.09620	2.849506	407.20510	5.351014
79.05483	2.349142	131.04940	100.000000	166.07750	1.620049	195.08080	24.494850	243.11740	1.977327	420.21780	33.653230
86.09695	3.554363	131.08580	2.778906	167.08590	23.531730	196.08770	6.633154	247.11210	4.680821	421.22220	5.200259
91.05473	8.312371	132.05280	4.832930	168.09200	2.293215	204.13920	1.451699	261.12770	10.021380	519.28750	2.258420
91.98430	2.259664	133.06510	10.363980	169.06520	10.197140	207.08070	4.133053	263.14290	1.509570	537.29680	34.799020
93.07031	2.022535	141.07020	7.522411	169.10150	2.227477	209.09670	2.044631	271.11260	2.313126	538.29730	6.587932
98.06068	1.783372	143.08580	6.402669	170.07240	2.557004	211.07560	3.393196	289.12270	16.906070		
103.05470	1.397815	144.05730	1.583442	171.08070	6.899018	219.08050	1.482622	290.12630	1.402141		
105.07020	4.333808	145.06510	7.693444	177.07030	3.189793	219.11720	1.265576	307.13340	15.436670		
107.04960	21.556500	152.06220	3.059322	179.08580	1.930415	221.09660	5.868309	308.13680	1.660697		
115.05450	2.848975	153.07020	5.098919	181.06500	5.792049	222.10370	2.156456	360.19680	3.546501		
116.07080	2.821137	154.07730	1.872616	182.07220	1.749920	223.07500	1.428078	374.21220	3.711244		
117.07020	8.158077	155.08560	5.798800	183.08100	13.401680	229.08540	1.432769	378.20730	6.051644		
119.04960	12.242210	157.06500	12.709360	184.07350	1.689836	231.08010	1.320400	392.22220	4.878211		

Figure S24. HRESIMS/MS spectrum of compound **2**.

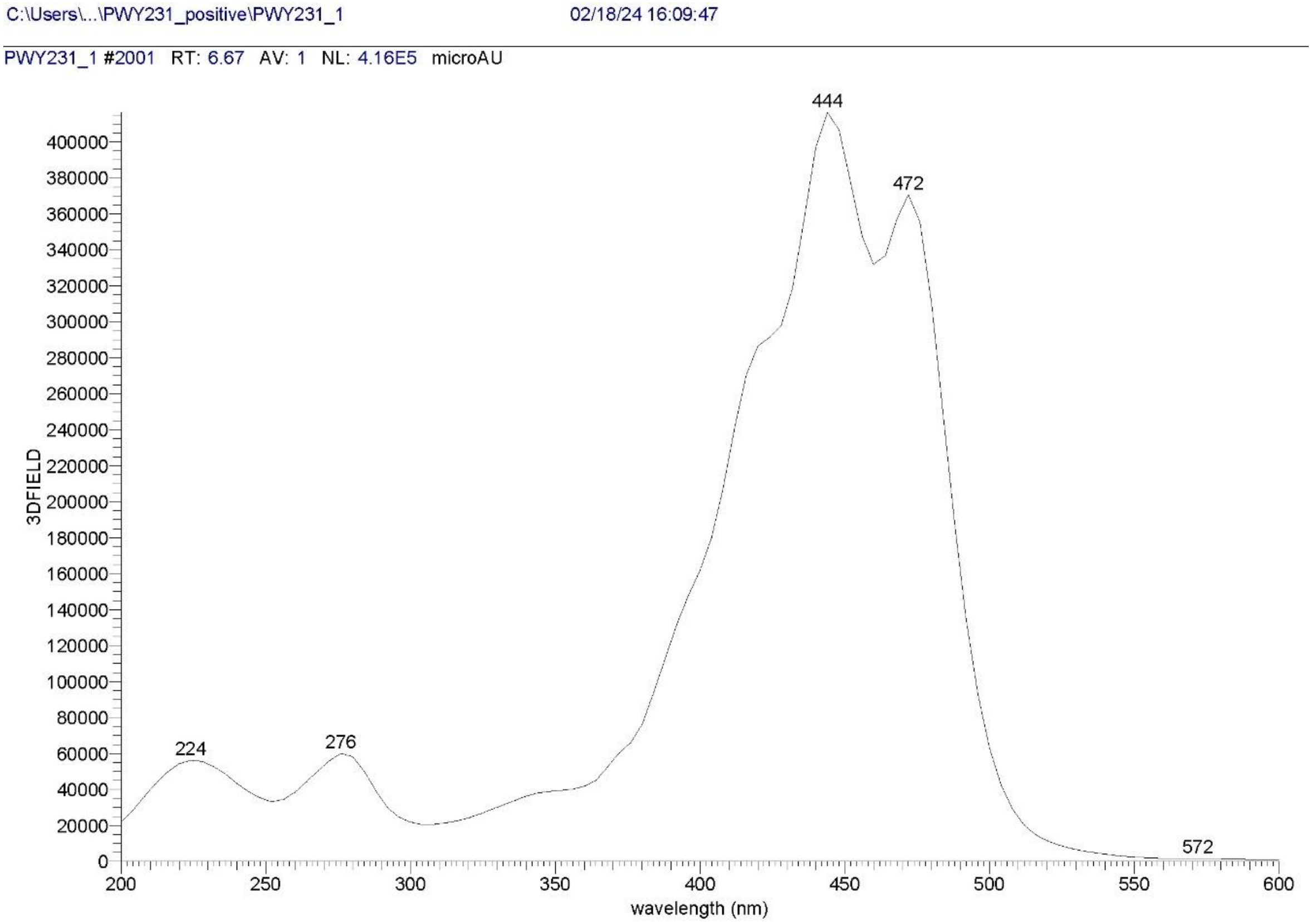

Figure S25. UV spectrum of compound **3**.

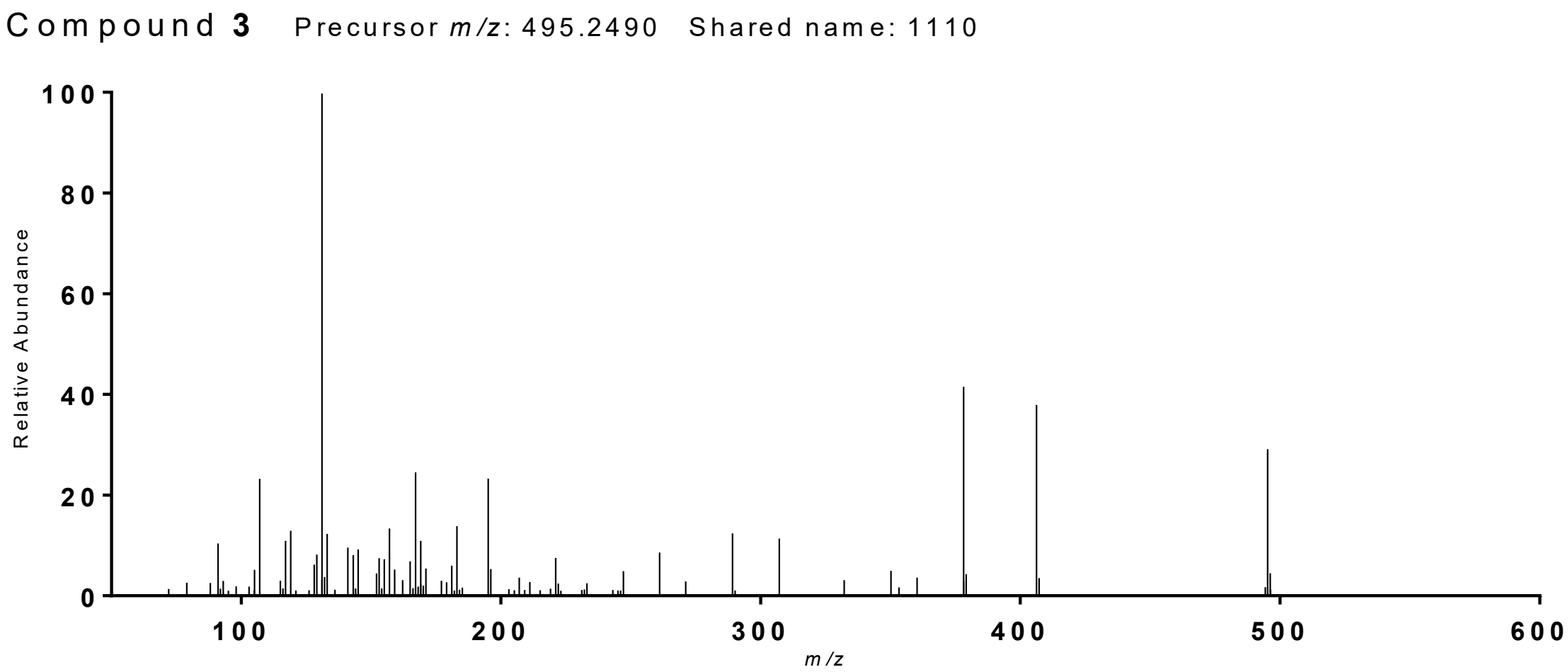

m/z	Relative Abundance	*m/z*	Relative Abundance	*m/z*	Relative Abundance	*m/z*	Relative Abundance	*m/z*	Relative Abundance	*m/z*	Relative Abundance
72.08146	1.352914	119.04960	12.921430	153.07010	7.447073	179.08570	2.684943	221.09640	7.499010	332.16410	3.099586
79.05477	2.610068	121.06510	1.046125	154.07760	1.469878	181.06490	5.964144	222.10350	2.441474	350.17540	4.937665
88.04000	2.523223	126.05530	1.125130	155.08580	7.251066	182.07140	1.069214	223.07610	0.977640	353.26750	1.682412
91.05481	10.412860	128.06250	6.165063	157.06510	13.384560	183.08080	13.847770	231.08090	1.160044	360.19600	3.635770
91.98522	1.425486	129.07030	8.176322	159.08070	5.206889	184.07360	1.186962	232.08860	1.255258	378.16960	41.503400
93.07027	2.957480	131.04940	100.000000	162.09140	3.129260	185.09620	1.616698	233.09650	2.507089	378.20640	2.912012
95.04944	1.014384	131.08570	3.174389	165.07010	6.855642	195.08090	23.275010	243.11650	1.138946	379.17320	4.268307
98.06057	1.867626	132.05250	3.721047	166.07760	1.491612	196.08760	5.320659	245.09650	1.053829	406.20200	37.904630
103.05470	1.797409	133.06510	12.319090	167.08580	24.508850	203.08620	1.323467	246.10370	1.043792	407.20530	3.533384
105.03380	1.105357	136.07600	1.181702	168.09190	1.847541	205.10190	1.116283	247.11200	4.926113	494.28570	1.710178
105.07040	5.176574	141.07010	9.590863	169.06500	10.893920	207.08100	3.636463	261.12770	8.592824	495.24950	29.134950
107.04960	23.216570	143.08570	8.107779	169.10160	2.223787	209.09620	1.161620	271.11160	2.829751	496.25110	4.471241
115.05440	2.999214	144.05710	1.442024	170.07290	2.046053	211.07560	2.720953	289.12290	12.377420	496.36380	1.236582
116.07120	1.482052	145.06500	9.194814	171.08080	5.435922	215.08660	1.126830	290.12590	1.039168		
117.07020	10.925030	152.06200	4.463755	177.07020	2.978212	219.08090	1.426401	307.13310	11.394020		

Figure S26. HRESIMS/MS spectrum of compound **3**.

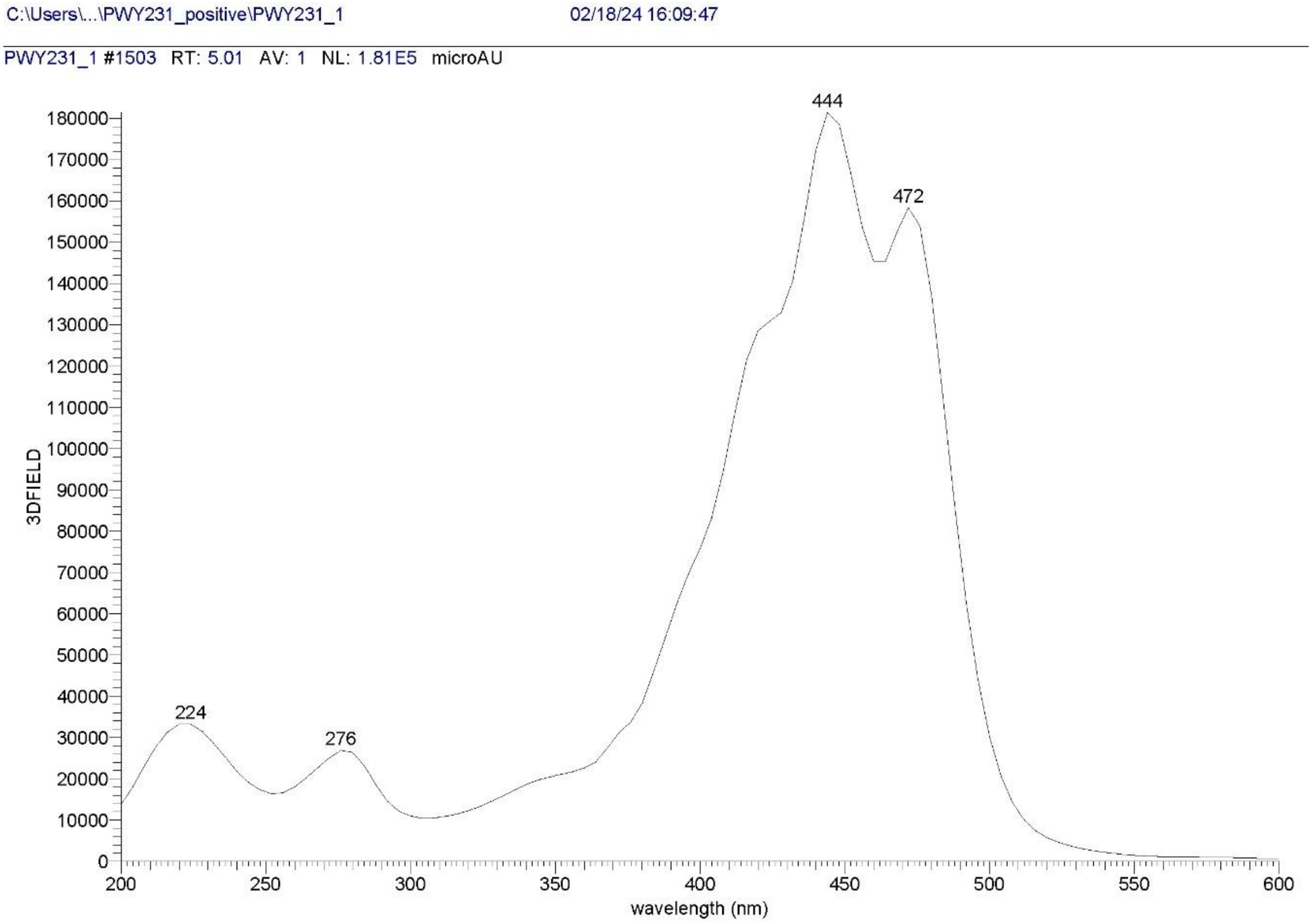

Figure S27. UV spectrum of compound **4**.

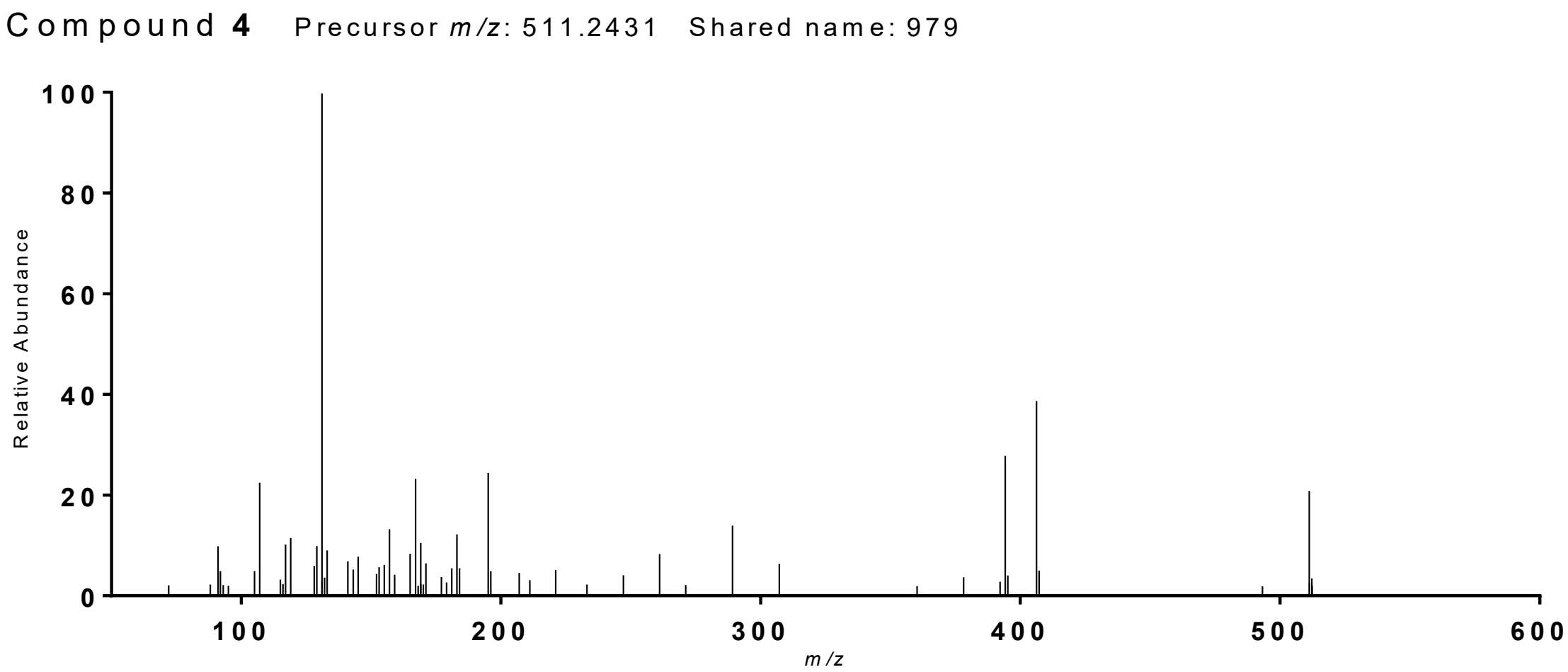

m/z	Relative Abundance	*m/z*	Relative Abundance	*m/z*	Relative Abundance	*m/z*	Relative Abundance	*m/z*	Relative Abundance
72.08135	2.062627	131.08530	2.868564	169.10120	2.281987	261.12750	8.310684	512.32190	1.975177
88.03951	2.228152	132.05250	3.611586	170.07250	2.219690	271.11200	2.144472		
91.05476	9.826426	133.06500	8.992427	171.08050	6.463217	289.12290	13.933310		
91.98266	4.918158	141.06980	6.841845	177.06960	3.744199	307.13320	6.341101		
93.07015	2.153837	143.08570	5.199682	179.08580	2.643152	360.19620	1.948330		
95.04958	1.998067	145.06500	7.770449	181.06490	5.445563	378.20660	3.655683		
105.07020	4.895688	152.06190	4.325942	183.08080	12.214200	392.18450	2.807971		
107.04960	22.445010	153.07000	5.690233	184.07320	5.467100	394.16530	27.774670		
115.05420	3.221757	155.08570	6.116551	195.08090	24.390120	395.16790	4.025911		
116.07090	2.347861	157.06510	13.235790	196.08790	4.894121	406.20220	38.664220		
117.07040	10.211730	159.08040	4.204641	207.08060	4.562708	407.20520	4.983949		
119.04950	11.492120	165.07000	8.333021	211.07500	3.119560	493.23150	1.881307		
128.06230	5.928873	167.08580	23.229290	221.09620	5.092709	511.24490	20.811250		
129.07020	9.881831	168.09100	1.978393	233.09580	2.234386	511.32620	2.558956		
131.04940	100.000000	169.06500	10.486740	247.11160	4.072213	512.24870	3.484049		

Figure S28. HRESIMS/MS spectrum of compound **4**.

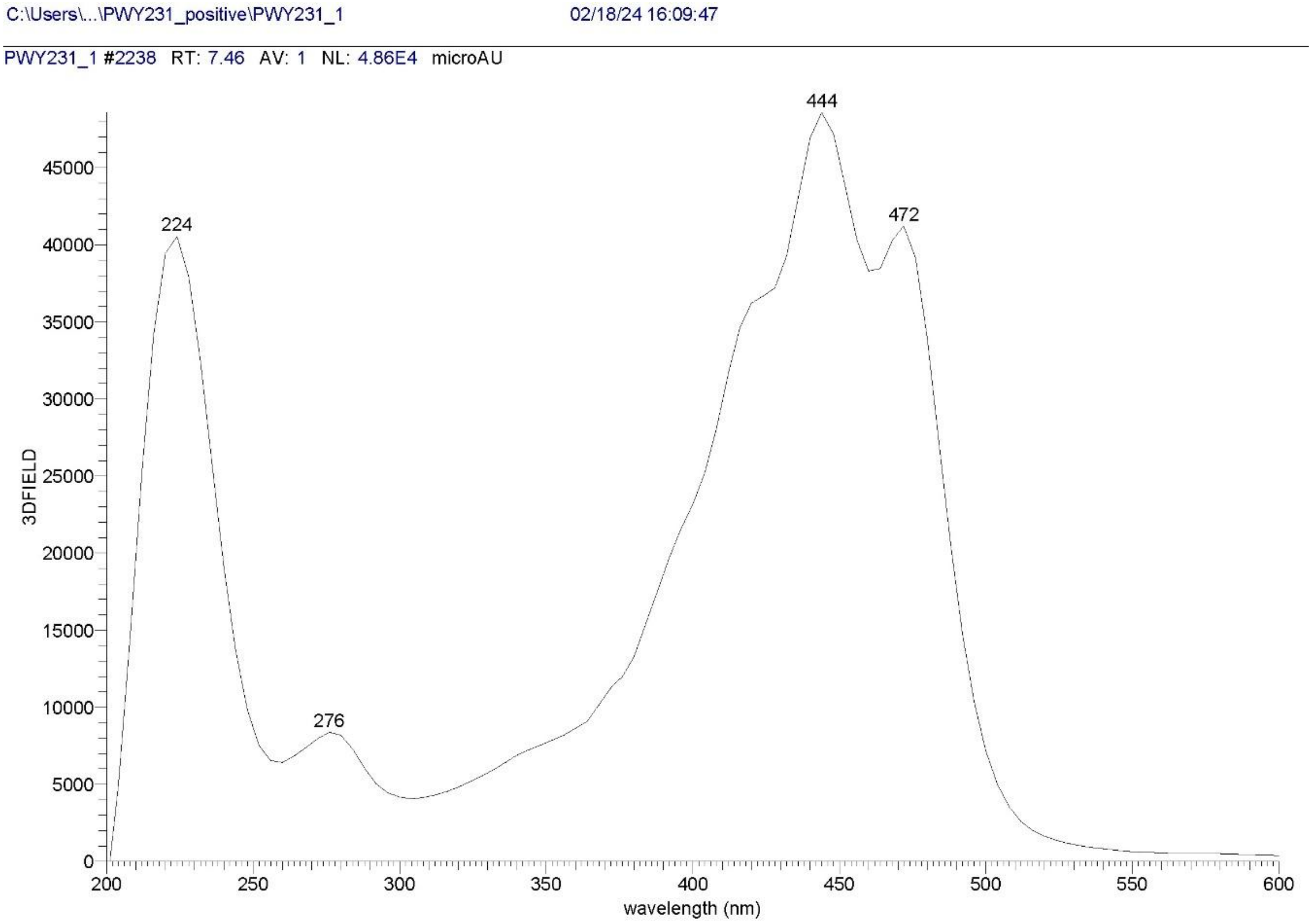

Figure S29. UV spectrum of compound **5**.

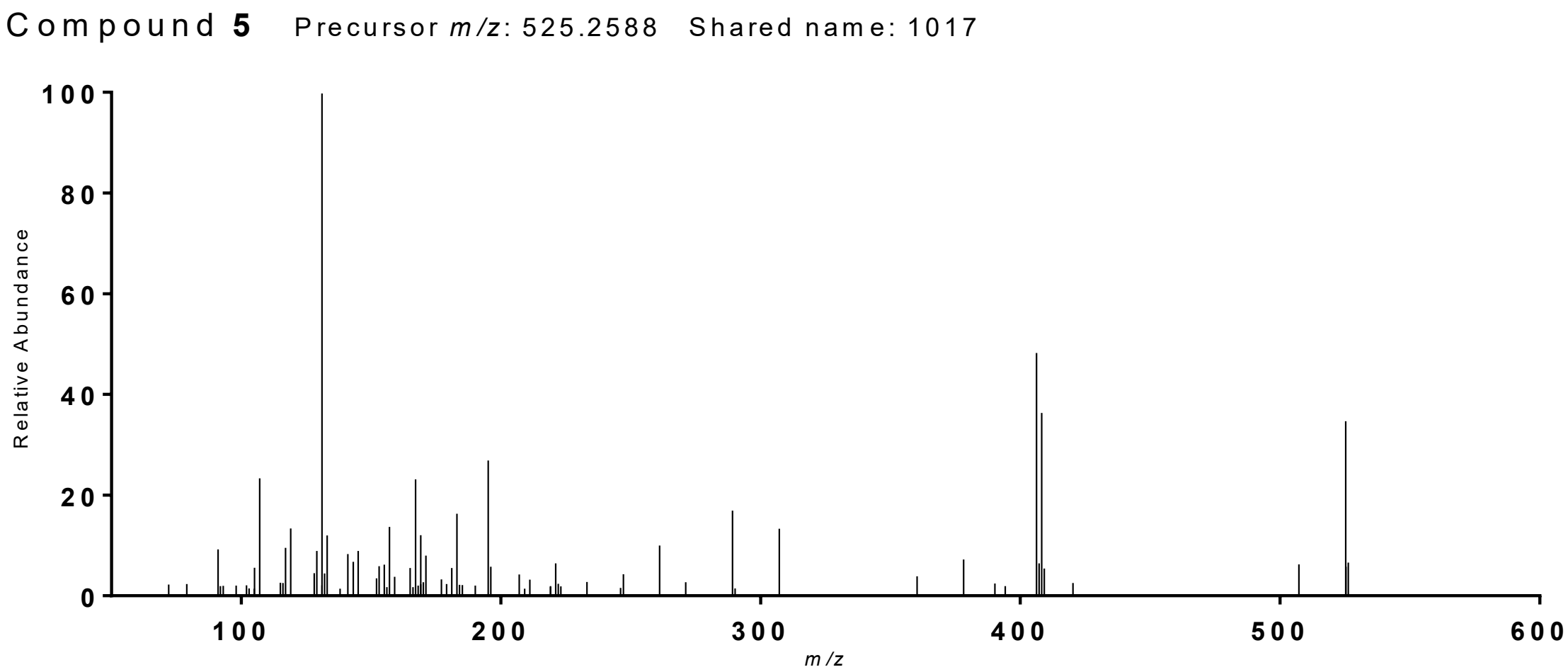

m/z	Relative Abundance	m/z	Relative Abundance	m/z	Relative Abundance	m/z	Relative Abundance	m/z	Relative Abundance	m/z	Relative Abundance
72.08140	2.224407	128.06220	4.493917	159.08080	3.768132	190.12320	2.020114	271.11270	2.696824	525.34300	5.774465
79.05471	2.345597	129.07020	8.890339	165.07020	5.534935	195.08080	26.898990	289.12270	16.935170	526.26380	6.599208
91.05475	9.211632	131.04940	100.000000	166.07770	1.707259	196.08790	5.786273	290.12550	1.466625		
91.98194	1.908325	131.08590	2.099853	167.08580	23.148700	207.08130	4.244658	307.13330	13.298630		
93.07022	1.998848	132.05270	4.416996	168.09150	2.031883	209.09680	1.423213	360.19570	3.900151		
98.06051	2.028024	133.06510	11.983800	169.06500	12.034010	211.07580	3.190216	378.20720	7.197044		
102.05540	2.090445	138.05530	1.416103	169.10150	2.295727	219.08150	1.944355	390.17160	2.427949		
103.05470	1.449388	141.07000	8.297449	170.07260	2.681281	219.11800	1.765045	394.16530	1.920919		
105.03420	1.394560	143.08580	6.767644	171.08070	7.985612	221.09650	6.439734	406.20220	48.215590		
105.07030	5.555639	145.06500	8.926471	177.07000	3.279053	222.10420	2.383513	407.20610	6.457408		
107.04960	23.310340	152.06240	3.479762	179.08600	2.358574	223.07570	1.887174	408.18130	36.311610		
115.05450	2.616921	153.07010	5.854262	181.06490	5.500799	233.09630	2.720801	409.18480	5.402836		
116.07090	2.543095	155.08570	6.169119	183.08080	16.290480	246.10470	1.564513	420.21800	2.521566		
117.07010	9.533107	156.06590	1.741675	184.07360	2.200960	247.11210	4.276390	507.24990	6.254280		
119.04950	13.377690	157.06510	13.685730	185.09670	2.107099	261.12760	9.984751	525.26110	34.700520		

Figure S30. HRESIMS/MS spectrum of compound **5**.

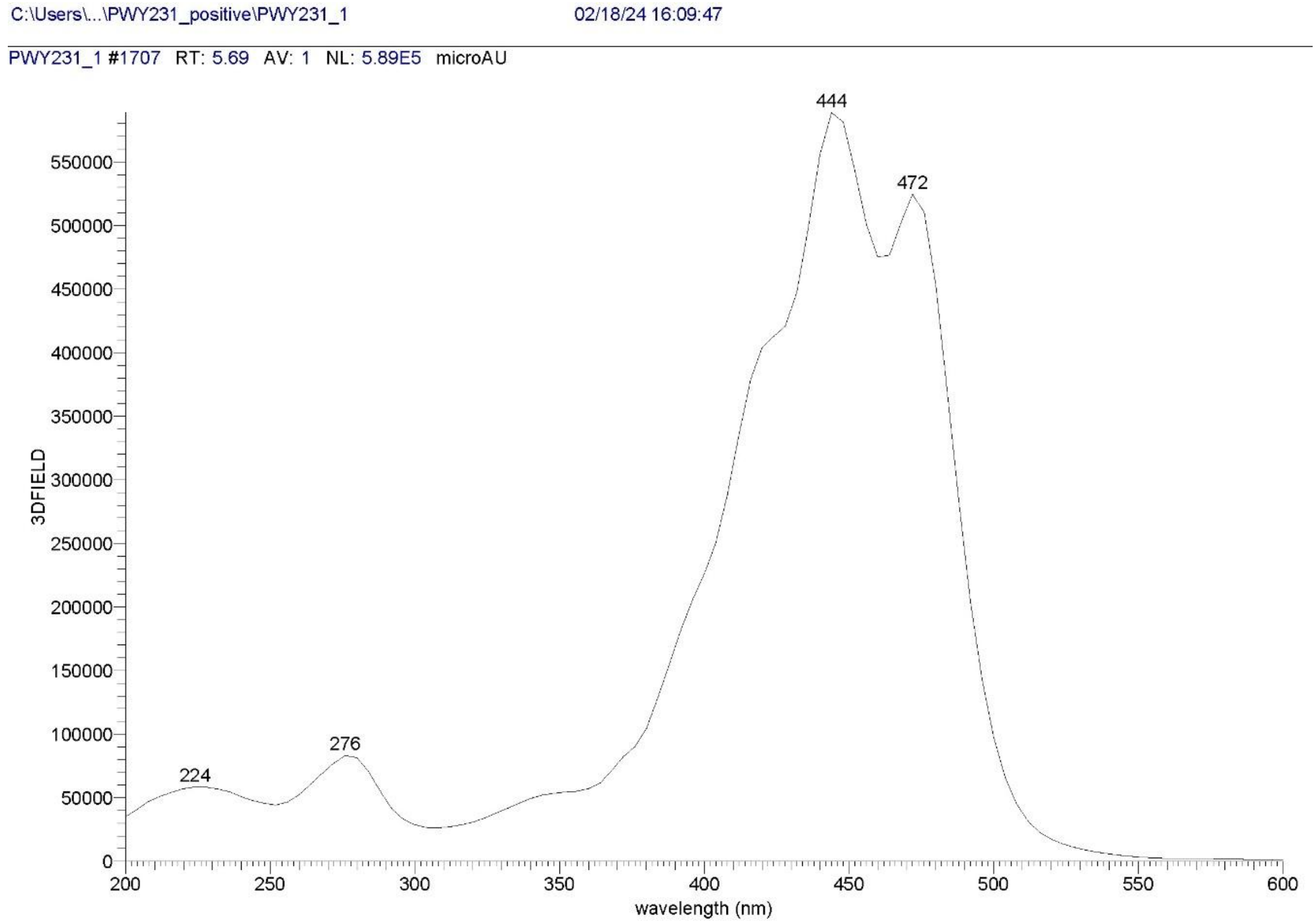

Figure S31. UV spectrum of compound **6**.

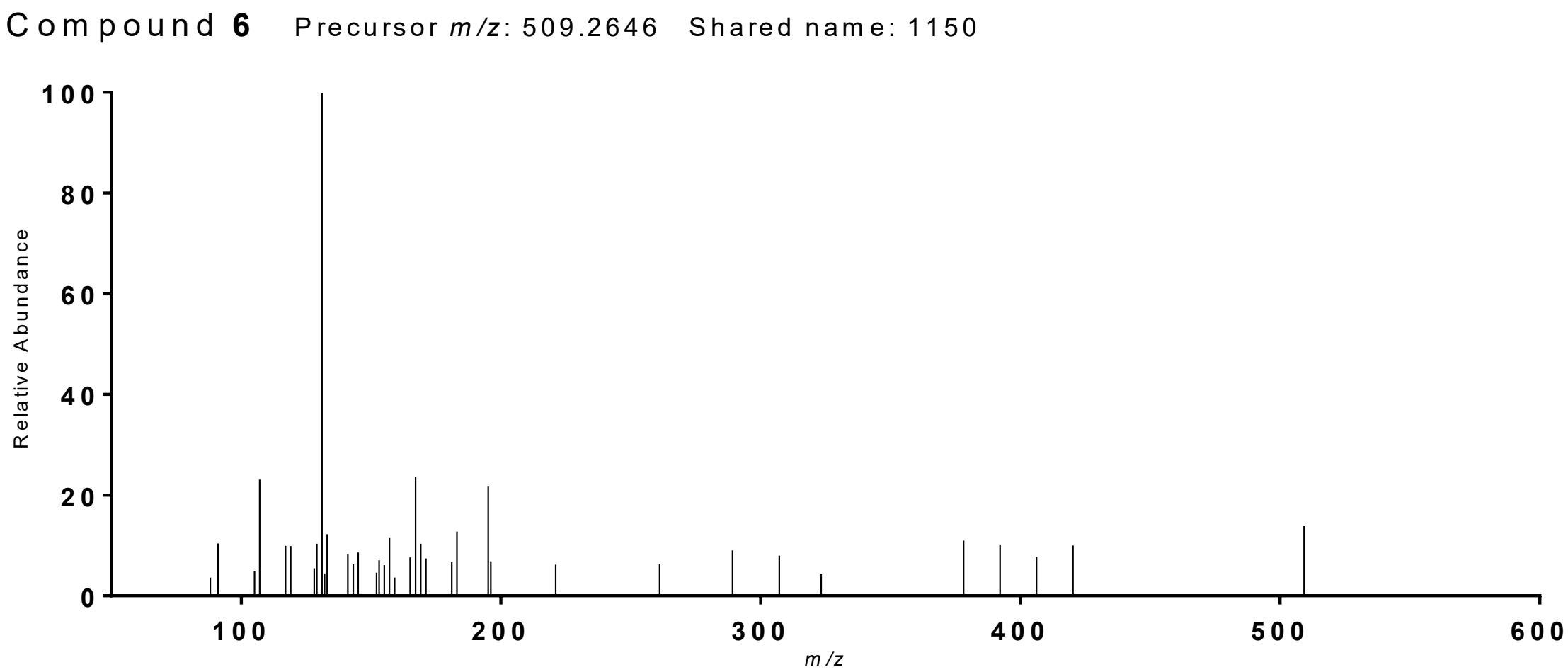

m/z	Relative Abundance	*m/z*	Relative Abundance	*m/z*	Relative Abundance
88.03970	3.604802	153.06990	7.046367	307.13300	7.958636
91.05483	10.382650	155.08510	6.100471	323.25860	4.412643
105.07010	4.836456	157.06530	11.498700	378.16960	10.980680
107.04960	23.098130	159.08060	3.632879	392.18440	10.169140
117.07000	9.933828	165.07060	7.642001	406.20070	7.748100
119.04970	9.869301	167.08600	23.628200	420.21830	9.959301
128.06210	5.489393	169.06520	10.333090	509.26640	13.821760
129.07040	10.341500	171.08100	7.406618		
131.04950	100.000000	181.06490	6.694406		
132.05240	4.423512	183.08090	12.757370		
133.06520	12.232480	195.08090	21.668030		
141.06940	8.293273	196.08760	6.839320		
143.08570	6.306434	221.09620	6.165498		
145.06480	8.590804	261.12740	6.255882		
152.06180	4.571348	289.12300	9.022536		

Figure S32. HRESIMS/MS spectrum of compound **6**.

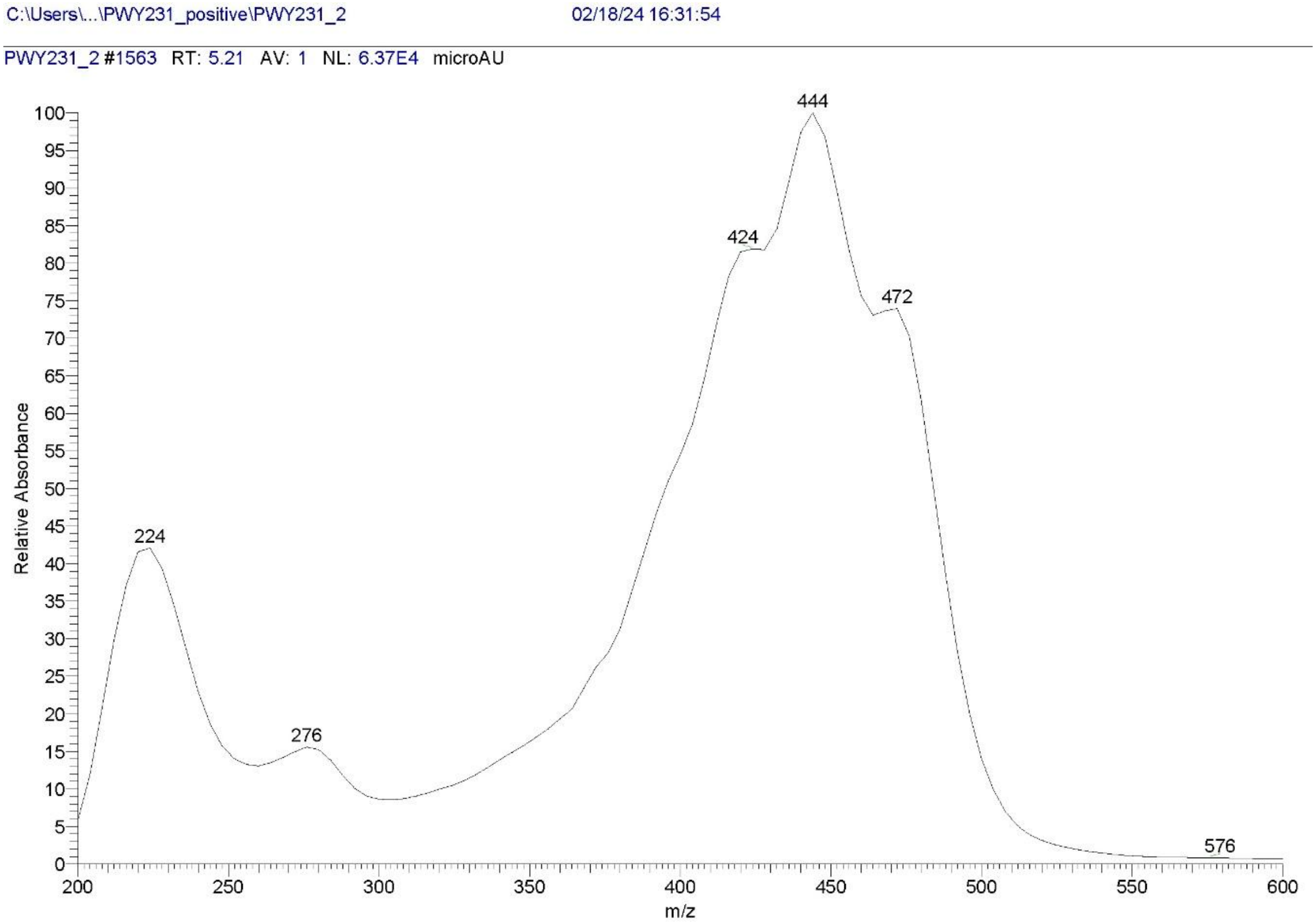

Figure S33. UV spectrum of compound **7**.

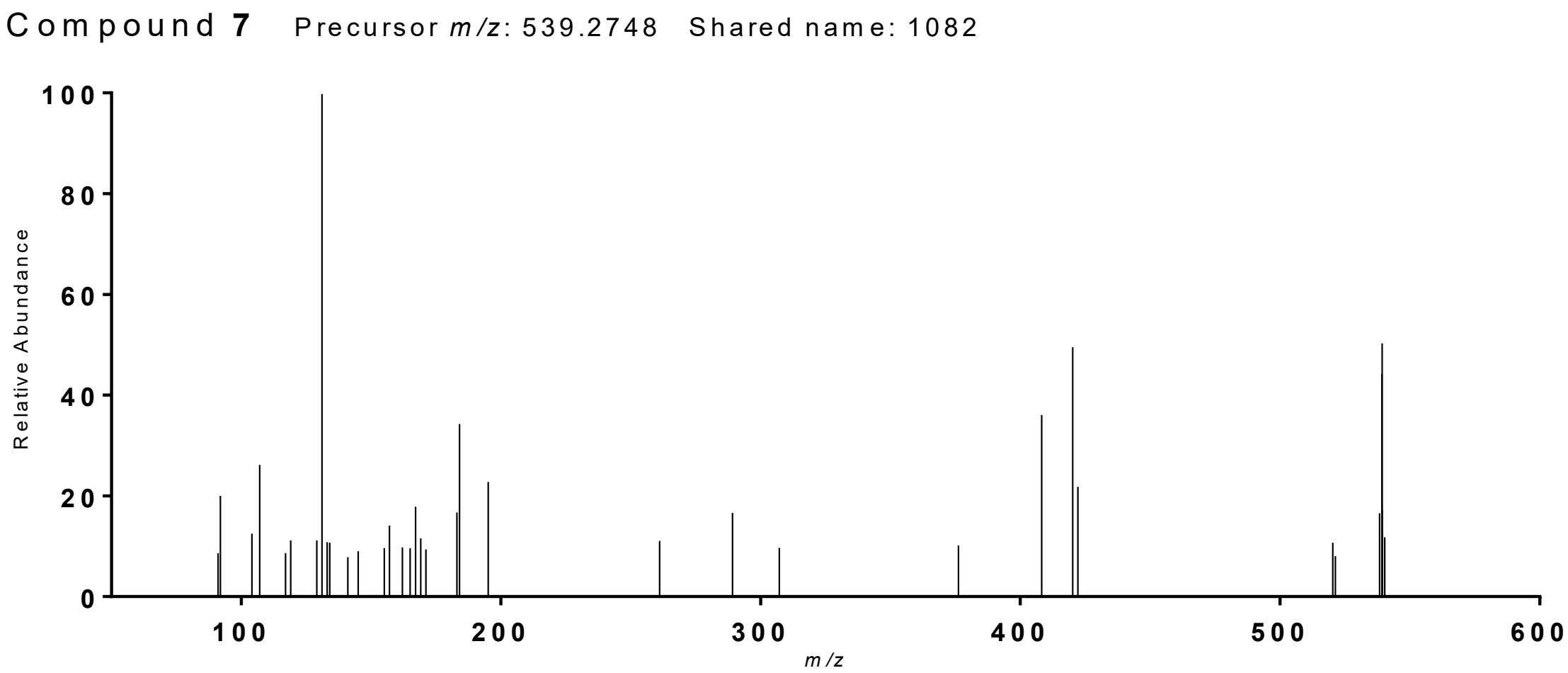

m/z	Relative Abundance	*m/z*	Relative Abundance	*m/z*	Relative Abundance
91.05466	8.587224	165.07050	9.628247	521.34480	8.058589
91.98466	20.010240	167.08570	17.815070	538.35240	16.576570
104.10710	12.521910	169.06480	11.570570	539.20880	44.149300
107.04960	26.137240	171.08070	9.348033	539.27640	50.267720
117.07010	8.656368	183.08050	16.717980	539.29020	17.238790
119.04940	11.146710	184.07340	34.249100	539.35970	12.541980
129.07010	11.165840	195.08080	22.785410	540.27940	11.768490
131.04940	100.000000	261.12700	11.081090		
133.06490	10.784420	289.12180	16.596880		
134.01860	10.712460	307.13290	9.689778		
141.06990	7.827214	376.18820	10.161340		
145.06470	9.028821	408.18110	36.045200		
155.08540	9.613296	420.21710	49.514940		
157.06510	14.069970	422.19560	21.779290		
162.04990	9.780322	520.33840	10.719270		

Figure S34. HRESIMS/MS spectrum of compound **7**.

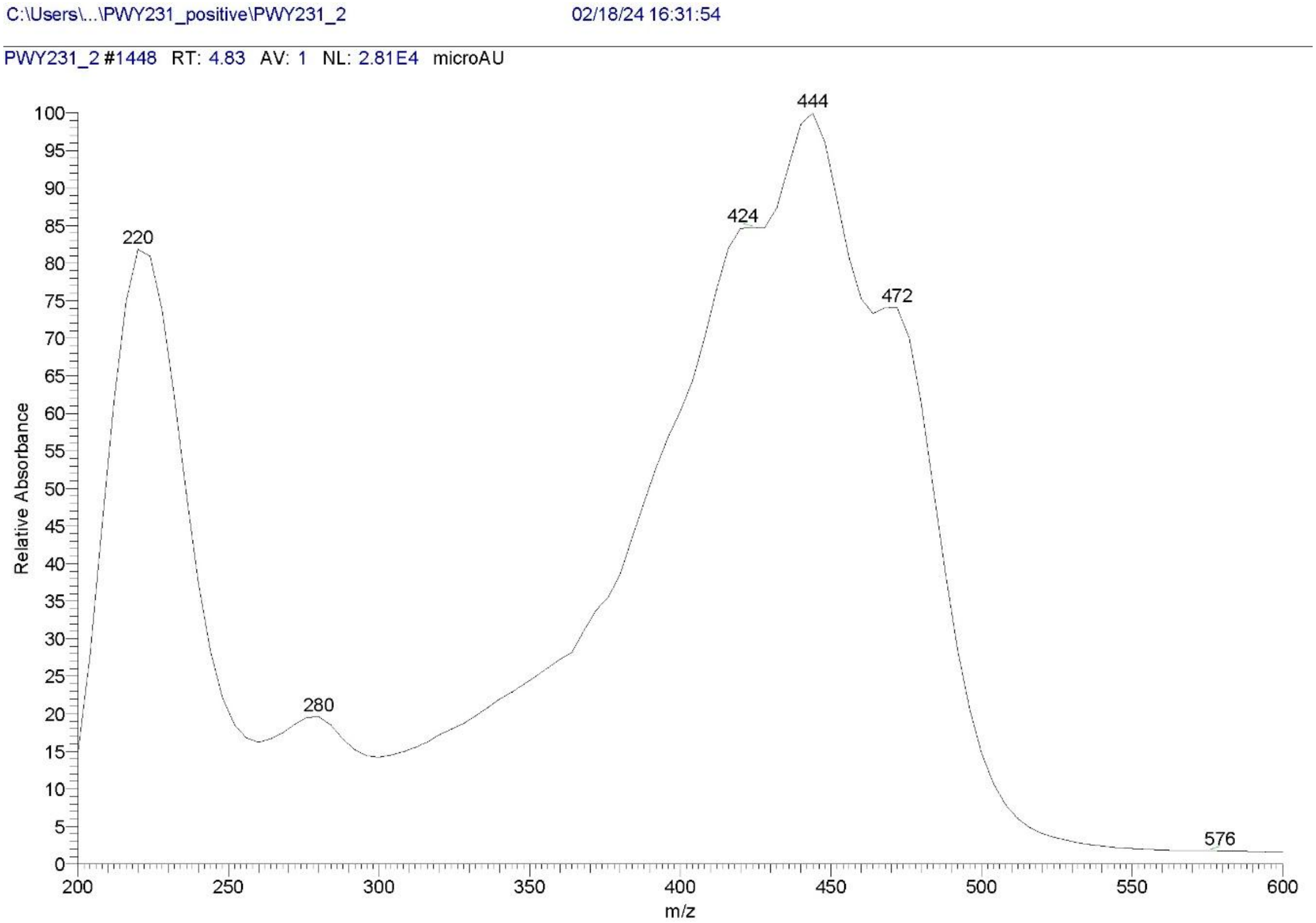

Figure S35. UV spectrum of compound **8**.

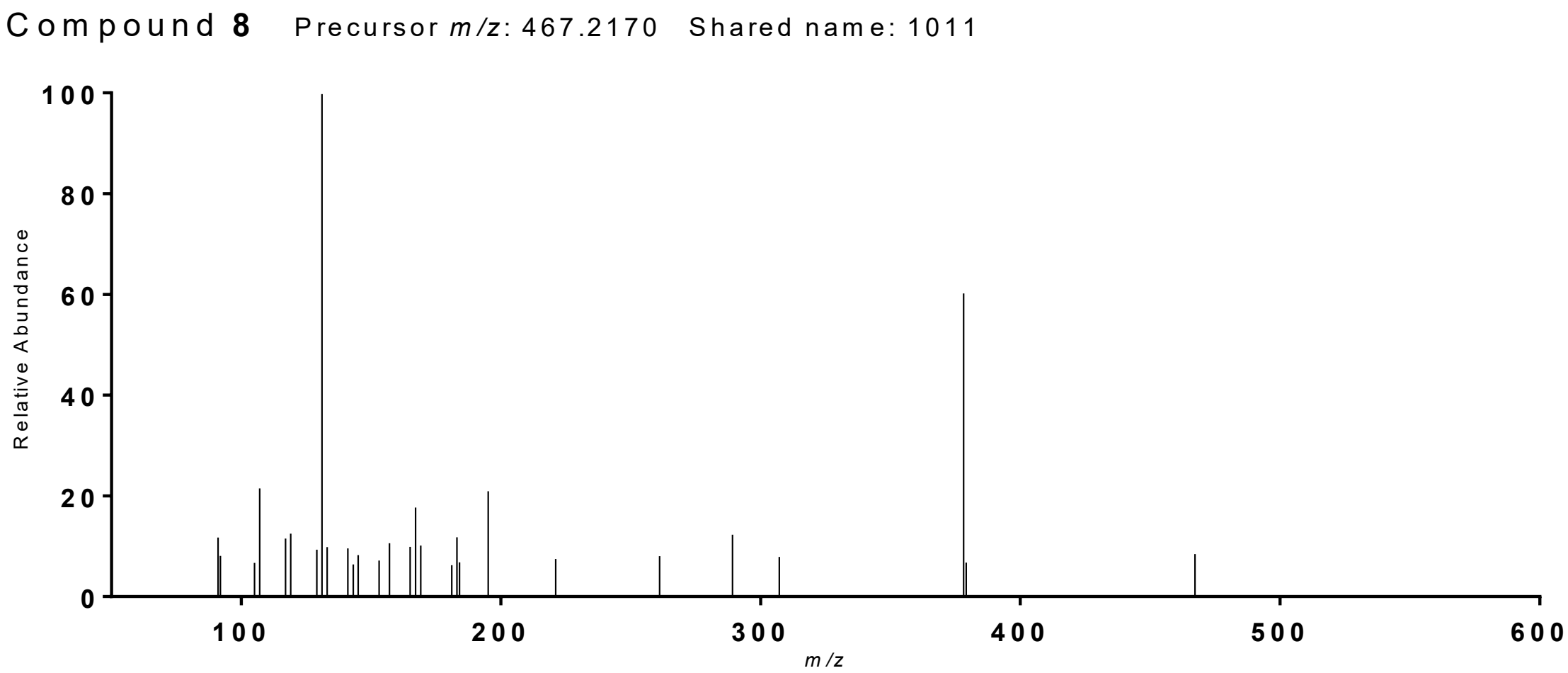

m/z	Relative Abundance	*m/z*	Relative Abundance
91.05474	11.704380	167.08620	17.709830
91.98615	8.095394	169.06470	10.137890
105.06970	6.721024	181.06460	6.255595
107.04960	21.466950	183.08100	11.780690
117.07010	11.549760	184.07390	6.815851
119.04930	12.478380	195.08100	20.917500
129.07000	9.321342	221.09620	7.463006
131.04950	100.000000	261.12780	8.049912
133.06500	9.806051	289.12300	12.319810
141.06990	9.570863	307.13320	7.893525
143.08580	6.387847	378.17050	60.179280
145.06500	8.255644	379.17360	6.739102
153.07010	7.152425	467.21730	8.447071
157.06490	10.608420		
165.07000	9.857458		

Figure S36. HRESIMS/MS spectrum of compound **8**.

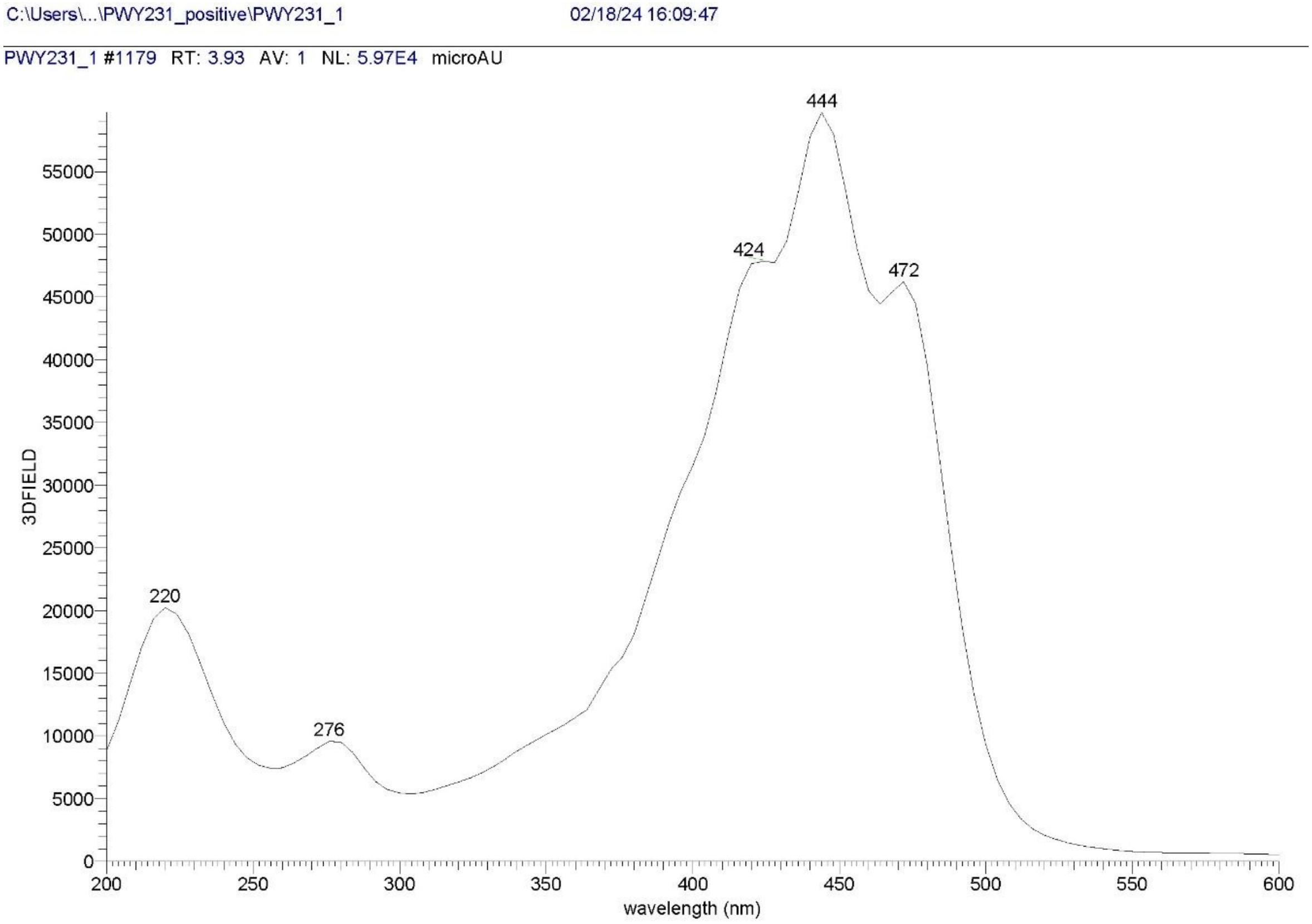

Figure S37. UV spectrum of compound **9**.

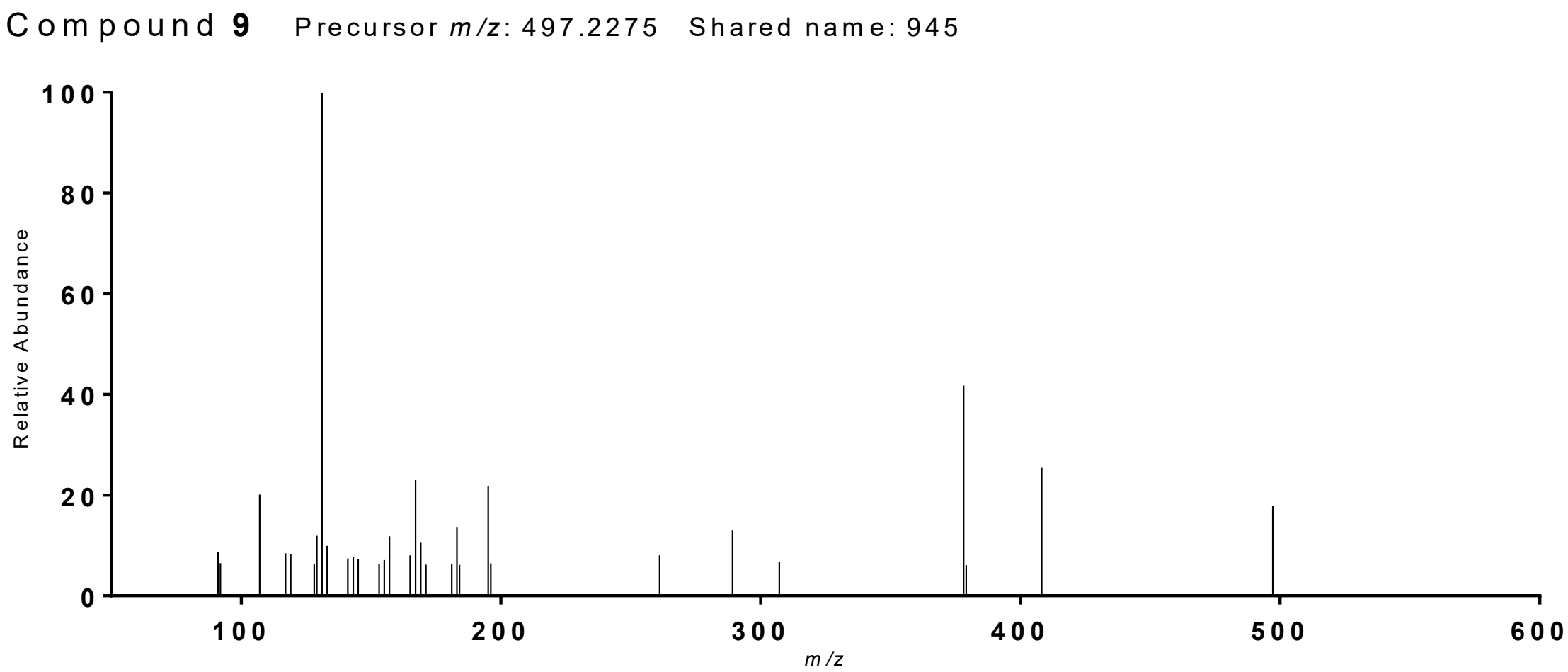

m/z	Relative Abundance	*m/z*	Relative Abundance	*m/z*	Relative Abundance
91.05455	8.647386	165.07020	8.014818	497.22600	17.789340
91.98464	6.492471	167.08580	22.949260		
107.04960	20.080380	169.06480	10.535030		
117.06990	8.427234	171.08070	6.199750		
119.04920	8.338371	181.06440	6.341685		
128.06200	6.365374	183.08080	13.692530		
129.06980	11.952110	184.07340	6.136555		
131.04950	100.000000	195.08110	21.802650		
133.06470	9.926787	196.08780	6.424553		
141.06980	7.401718	261.12670	8.035208		
143.08540	7.762813	289.12220	12.960160		
145.06490	7.368526	307.13370	6.776286		
153.06920	6.342158	378.17070	41.783600		
155.08540	7.134353	379.17350	6.063160		
157.06490	11.837270	408.18060	25.418820		

Figure S38. HRESIMS/MS spectrum of compound **9**.

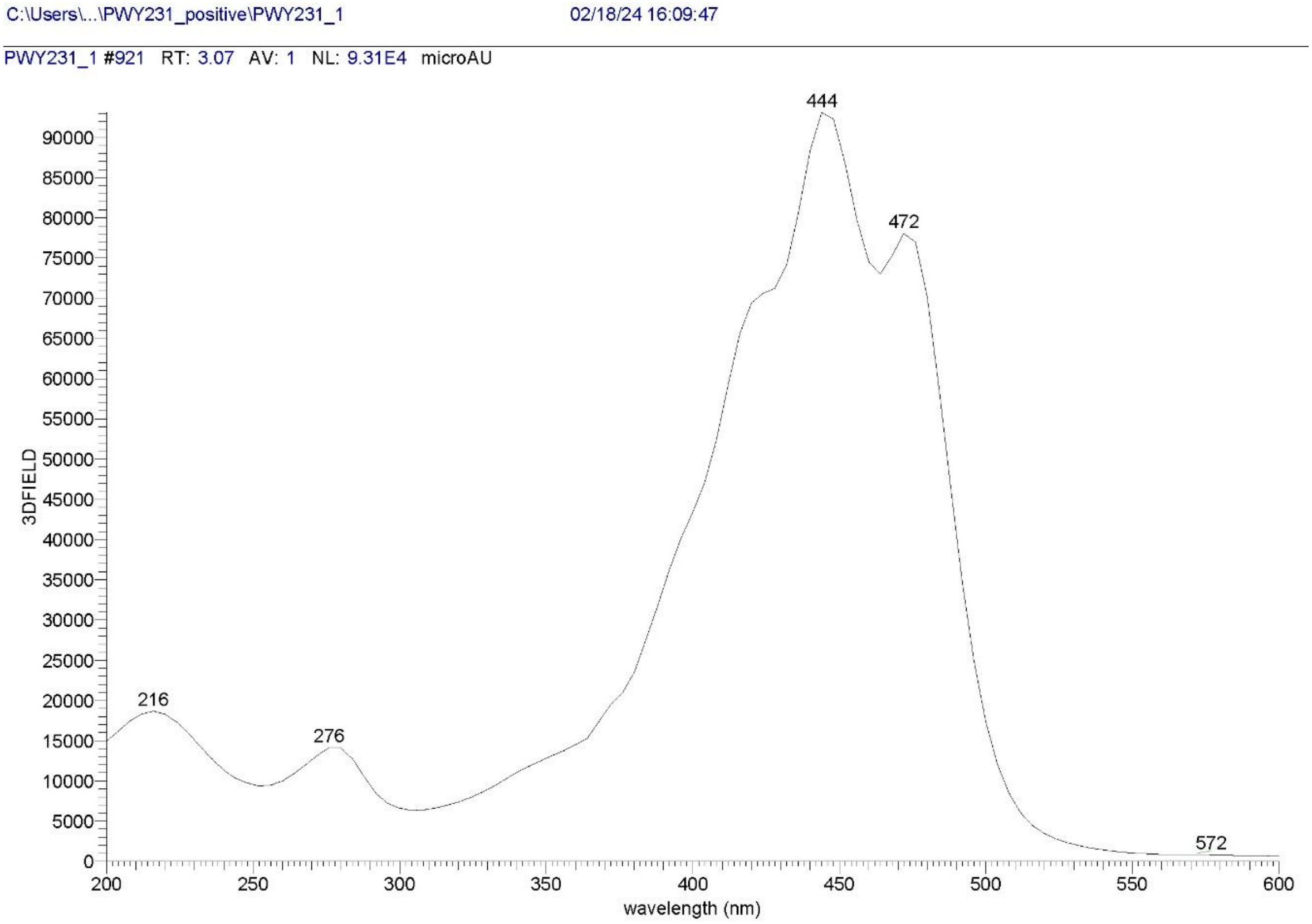

Figure S39. UV spectrum of compound **10**.

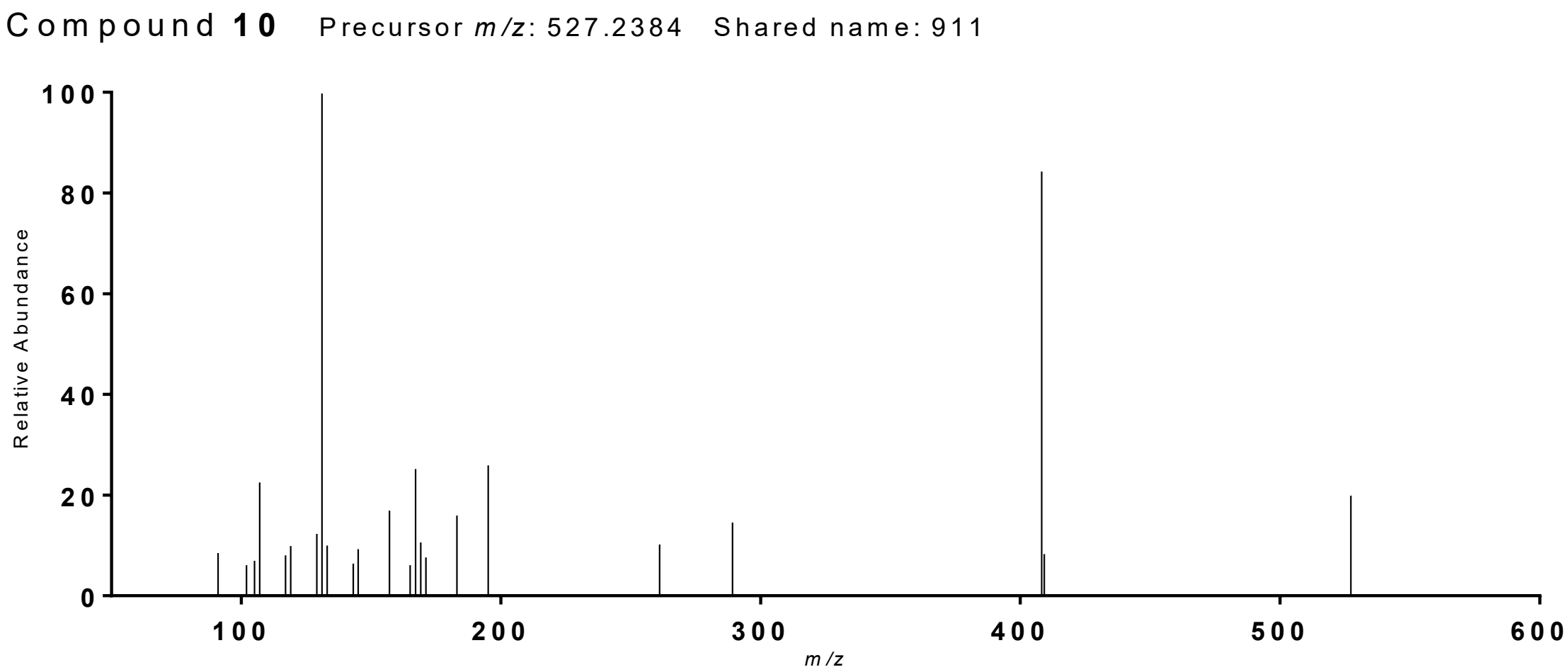

m/z	Relative Abundance	*m/z*	Relative Abundance
91.05459	8.479799	171.08110	7.638378
102.05540	6.102026	183.08100	15.920060
105.07020	6.958963	195.08070	25.876920
107.04950	22.487370	261.12760	10.176180
117.07010	8.019829	289.12300	14.556190
119.04930	9.859413	408.18140	84.289000
129.07030	12.299570	409.18500	8.299403
131.04950	100.000000	527.23620	19.894410
133.06470	9.997504		
143.08550	6.371120		
145.06470	9.273020		
157.06500	16.902730		
165.06990	6.107182		
167.08560	25.173950		
169.06480	10.611140		

Figure S40. HRESIMS/MS spectrum of compound **10**.

Table S1. Preliminary concentration screening of cordylutene A (**1**) in the A2E/blue light–challenged ARPE-19 model

Treatment		Concentration (μg/mL)	Cell Viability (%)
Control		-	100.0 ± 3.2
Model (A2E + blue light)	-	-	68.0 ± 3.5
	Lutein	17.1	73.8 ± 2.0
	Cordylutene A (**1**)	10	84.5 ± 0.9
		5	87.6 ± 2.2
		2.5	91.4 ± 4.3

Data are expressed as mean ± SD (n = 3). ARPE-19 cells were pretreated with cordylutene A or lutein for 24 h, followed by incubation with A2E (5 μM) and blue light irradiation for 5 min (preliminary screening conditions). Cell viability was determined by the MTT assay and is presented relative to the untreated control group, which was set at 100%. These preliminary data were used to support selection of the concentration range of cordylutene A for the formal photodamage assay.

Table S2. Preliminary optimization of blue light exposure time in A2E-loaded ARPE-19 cells

Group	A2E (μM)	Cell Reduction (%) at Blue Light Exposure Time (min)				
		0	5	10	15	20
**Control**		0.0	0.0	0.0	0.0	0.0
**A2E-treated**	**5**	4.5	5.5	19.8	31.0	65.4
**A2E-treated**	**10**	6.5	18.5	76.4	88.5	92.0
**A2E-treated**	**20**	4.3	87.1	90.0	91.4	92.9

Data are presented as percentage reduction in cell viability relative to the untreated control. ARPE-19 cells were loaded with A2E at the indicated concentrations and then exposed to blue light (420–439 nm) for the indicated durations. These preliminary data were used to select the irradiation time for the formal photodamage assay.
